# Supplementary material for: DELTA: Strengthening human biological resilience with an N=1 digital health and dynamic biomarker protocol
Source: PLoS One. 2026 Aug 12;21(8):e0354234. doi: 10.1371/journal.pone.0354234 (PMC13465858; doi:10.1371/journal.pone.0354234)
Supplement: Supplementary file 1 [file pone.0354234.s001.pdf]

DELTA: Strengthening Human Biological Resilience with an N-of-1 Digital Health and Dynamic Biomarker Protocol

Peter Wang#, Nigel Foo, Chengxun Su, Nicole Yong Ting Leung, Shang Wei Song, Gyula Seres, Yoann Sapanel, Lissa Hooi, Avelle Wong, Yoong Hun Ong, Poonam Rai, Hyunjin Park, Han Shi Jocelyn Chew, Laureen Yi Ting Wang, Jonathan Wei Jie Lee, Xavier Tadeo#, Dean Ho#

#Co-Corresponding Authors: D.H. (biedh@nus.edu.sg); X.T. (lsixtc@nus.edu.sg); P.W. (lsipww@nus.edu.sg)

This S1 File includes:

- Tables 1-5
- Figs. 1-18

Table 1. DELTA001's supplement regimen. The compounds and their dosing schedules are summarized below.

Compound	Dose/Unit	Regimen	Brand
Creatine Monohydrate	500mg/capsule	3g/day (6 capsules)	Life Extension
Omega-3	1000mg/softgel (540mg EPA/360mg DHA)	3000mg/day (3 Gels)	Now
Vitamin B Complex	Per 2 capsules. Thiamine (Vitamin B1) (as thiamine HCl) — 100 mg<br>Riboflavin (Vitamin B2) (as riboflavin and riboflavin 5'-phosphate) — 75 mg<br>Niacin (as niacinamide and niacin) — 100 mg<br>Vitamin B6 (as pyridoxine HCl and pyridoxal 5'-phosphate) — 100 mg<br>Folate (as L-5-methyltetrahydrofolate, calcium salt) — 680 mcg DFE<br>Vitamin B12 (as methylcobalamin) — 300 mcg<br>Biotin — 1000 mcg<br>Pantothenic acid (as D-calcium pantothenate) — 500 mg<br>Calcium (as D-calcium pantothenate, dicalcium phosphate) — 50 mg<br>Inositol — 100 mg<br>PABA (para-aminobenzoic acid) — 50 mg	2 capsules/day	Life Extension
Multivitamin (One a Day)	Vitamin A (100% as beta-carotene), 900 mcg<br>Vitamin C, 99 mg<br>Vitamin D, 25 mcg (1000 IU)<br>Vitamin E, 15 mg<br>Thiamin (B1), 1.32 mg<br>Riboflavin (B2), 1.43 mg<br>Niacin, 17.6 mg<br>Vitamin B6, 2.17 mg<br>Folate (240 mcg folic acid), 400 mcg DFE<br>Vitamin B12, 6.24 mcg<br>Biotin, 43 mcg<br>Pantothenic Acid, 15.5 mg<br>Calcium, 210 mg<br>Iron, 0 mg<br>Iodine, 150 mcg<br>Magnesium, 120 mg<br>Zinc, 11 mg<br>Selenium, 55 mcg<br>Copper, 0.9 mg<br>Manganese, 2.3 mg<br>Chromium, 35 mcg<br>Lycopene, 300 mcg	1 tablet/day	Bayer (One a Day)
Vitamin D3	1000IU/gel	2000 IU/day (2 gels)	Now
Plant Sterols	400mg/gel	2000 mg/day (5 gels)	Swansons

Curcumin C3 Complex	500mg/capsule	500 mg/day (1 capsule)	California Gold Nutrition - Strict adherence to recommended dose
Ubiquinol	200mg/gel	200 mg/day (1 gel)	Now
Quercetin/Bromelain	Per 2 capsules Quercetin 800mg<br>Bromeliad (2400 GDU/g). 165mg	4 capsules/day Quercetin 1600mg Bromeliad (2400 GDU/g). 330mg	Now
Vitamin K2 (MK-7)	100mcg/capsule	100mcg/day (1 capsule)	Now
EGCg	400mg/capsule	400 mg/day (1 capsule)	Now - Strict adherence to recommended dose
Probiotic	Per 1 capsule<br>Lactobacillus acidophilus (La-14)<br>Bifidobacterium lactis (Bl-04)<br>Lactobacillus plantarum (Lp-115)<br>Lactobacillus casei (Lc-11)<br>Lactobacillus rhamnosus (Lr-32)<br>Lactobacillus paracasei (Lpc-37)<br>Bifidobacterium breve (Bb-03)<br>Streptococcus thermophilus (St-21)<br>Lactobacillus salivarius (Ls-33)<br>Bifidobacterium longum (Bl-05)	100 billion CFU/day (1 capsule)	Now

Table 2. Summary of biomarkers measured in the DELTA protocol.

Biomarker	Purpose	Collection Method
Heart Rate Variability (HRV)	Stress resilience and recovery	Wearables
Resting Heart Rate (RHR)	Heart fitness and cardiovascular health	Wearables
Blood Pressure	Heart health and risks for cardiovascular diseases	Omron IntelliSense BP7350
Grip Strength	Physical health (e.g., muscle function, frailty) and longevity	Camry EH101
Blood glucose	Carbohydrate metabolism and blood sugar regulation	Finger stick (Abbott Optium Neo)
Blood ketones	Fat metabolism and ketosis	Finger stick (Abbott Optium Neo)
HbA1c	Indicator of diabetes risk	Abbott Libre
Apolipoprotein B (ApoB)	Cardiovascular risk, a better indicator of ‘bad’ cholesterol than LDL	Blood test
Apolipoprotein A (ApoA)	Cardiovascular protection, a better indicator of ‘good’ cholesterol than HDL	Blood test
High-sensitivity C-reactive protein (hs-CRP)	Systemic inflammation	Blood test
Homocysteine	Methylation and B-vitamin status	Blood test
Gut Microbiome	Composition of gut microbiome as indicator of overall health	16S rRNA gene sequencing (Amili Pte. Ltd.)

Table 3. Duration of sleep stages recorded with Garmin, Apple Watch, and WHOOP devices.

Devices	Deep Sleep (Mins)	Light Sleep (Mins)	REM Sleep (Mins)	Awake (Mins)
Garmin	79.98	287.7	104.3	20.03
Apple	40.92	342.3	99.91	16.34
WHOOP	93.55	222.5	120.8	53.88

Table 4. SPIRIT extension and elaboration of N-of-1 trials (SPENT) checklist for N-of-1 protocol reporting guidelines

SECTION / TOPIC	ITEM NO.	SPIRIT 2013	ITEM NO.	SPENT 2019	ON PAGE
**SECTION 1: ADMINISTRATIVE DATA**					
Title	1	Descriptive title identifying the study design, population, interventions, and, if applicable, trial acronym.	1a	Descriptive title, including "N-of-1 trial" and "protocol". ***For series:*** Descriptive title, including "a series of N-of-1 trials" and "protocol".	Pg.1
		—	1b	For specific guidance on abstracts, see SPENT Guidance for Abstracts. (Appendix table 2)	N/A
Trial Registration	2a	Trial identifier and registry name.<br>If not yet registered, name of intended registry.	2a	(no change)	Pg.20
	2b	All items from the World Health Organization Trial Registration Data Set. (WHOTRDS)	2b	(no change)	N/A
Protocol version	3	Date and version identifier.	3	(no change)	N/A
Funding	4	Sources and types of financial, material, and other support.	4	(no change)	Pg.24
Roles and responsibilities	5a	Names, affiliations, and roles of protocol contributors.	5a	(no change)	Pg.24-25
	5b	Name and contact information for the trial sponsor.	5b	(no change)	N/A
	5c	Role of study sponsor and funders, if any, in study design; collection, management, analysis, and interpretation of data; writing of the report; and the decision to submit the report for publication, including whether they will have ultimate authority over any of these activities.	5c	(no change)	N/A
	5d	Composition, roles, and responsibilities of the coordinating center, steering committee, end point adjudication committee, data management team, and other individuals or groups overseeing the trial, if applicable. (see Item 21a for DMC)	5d	(no change)	N/A
**SECTION 2: INTRODUCTION**					
Background and rationale	6a	Description of research question and justification for undertaking the trial, including summary of relevant studies (published and unpublished) examining benefits and harms for each intervention.	6a	Description of research question and justification for undertaking the trial, including summary of relevant studies (published and unpublished) examining benefits and harms for each intervention, and rationale for using N-of-1.	Pg.2-4

	6b	Explanation for choice of comparators.	6b	(no change, SPENT commentary)	
Objectives	7	Specific objectives or hypotheses.	7	(no change)	
Trial design	8	Description of trial design, including type of trial (eg, parallel group, crossover, factorial, single group), allocation ratio, and framework (eg, superiority, equivalence, non-inferiority, exploratory).	8	Description of the trial design, including N-of-1 trial or series of trials, and framework (eg, superiority, equivalence, non-inferiority, exploratory). ***In addition for series:*** Explanation of the series design including whether the design will be tailored to each participant.	
**SECTION 3: METHODS**					
Participants, interventions, and outcomes					
Study Setting	9	Description of study settings (eg, community clinic, academic hospital) and list of countries where data will be collected. Reference to where list of study sites can be obtained.	9	(no change)	
Eligibility criteria	10	Inclusion and exclusion criteria for participants. If applicable, eligibility criteria for study centers and individuals who will perform the interventions (eg, surgeons, psychotherapists).	10	(in addition) Diagnosis/disorder, diagnostic criteria, co-morbid conditions and concurrent therapies. ***For series:*** Same as SPIRIT item 10.	
Interventions	11a	Interventions for each group with sufficient detail to allow replication, including how and when they will be administered.	11a	Intervention(s) for each period with sufficient detail to allow replication, including how and when they will be administered, planned number of periods, and duration of each period (including run-in and washout, if applicable).<br>***In addition for series:*** How the design will be tailored to each participant, if applicable.	
	11b	Criteria for discontinuing or modifying allocated interventions for a given trial participant (eg, drug dose change in response to harms, participant request, or improving/ worsening disease).	11b	(no change, SPENT commentary)	
	11c	Strategies to improve adherence to intervention protocols, and any procedures for monitoring adherence (eg, drug tablet return, laboratory tests).	11c	(no change)	
	11d	Relevant concomitant care and interventions that are permitted or prohibited during the trial.	11d	(no change)	
Outcomes	12	Primary, secondary, and other outcomes, including the specific measurement variable (eg, systolic blood pressure), analysis metric (eg, change from baseline, final value, time to event),	12	(no change)	

		method of aggregation (eg, median, proportion), and time point for each outcome. Explanation of the clinical relevance of chosen efficacy and harm outcomes is strongly recommended.		
Participant timeline	13	Time schedule of enrollment, interventions (including any run-ins and washouts), assessments, and visits for participants. A schematic diagram is highly recommended (Figure).	13	(no change)
Sample size	14	Estimated number of participants needed to achieve study objectives and how it was determined, including clinical and statistical assumptions supporting any sample size calculations.	14	Estimated number of intervention periods and measurements/observations needed to achieve study objectives within an individual N-of-1 trial. ***In addition for series:*** Estimated number of participants needed to achieve study objectives. How these numbers were determined, including clinical and statistical assumptions supporting any sample size calculations.
Recruitment	15	Strategies for achieving adequate participant enrollment to reach target sample size.	15	***For series:*** Strategies for achieving adequate participant enrollment to reach target sample size.
Assignment of interventions (for controlled trials)				
Allocation sequence generation	16a	Method of generating the allocation sequence (eg, computer-generated random numbers), and list of any factors for stratification. To reduce predictability of a random sequence, details of any planned restriction (eg, blocking) should be provided in a separate document that is unavailable to those who enrol participants or assign interventions.	16a	Method of generating the allocation sequence (eg, computer-generated random numbers), and list of any factors for stratification. To reduce predictability, details of any restrictions (eg, pairs, blocking) should be provided in a separate document that is unavailable to those who enrol participants or assign interventions. ***In addition for series:*** List of any factors for stratification.
concealment mechanism	16b	Mechanism of implementing the allocation sequence (eg, central telephone; sequentially numbered, opaque, sealed envelopes), describing any steps to conceal the sequence until interventions are assigned.	16b	(no change, SPENT commentary)
implementation	16c	Who will generate the allocation sequence, who will enroll participants, and who will assign participants to interventions.	16c	(no change)
Blinding (masking)	17a	Who will be blinded after assignment to interventions (eg, trial participants, care providers, outcome assessors, data analysts), and how.	17a	(no change)

	17b	If blinded, circumstances under which unblinding is permissible, and procedure for revealing a participant's allocated intervention during the trial.	17b	(no change)	
Data collection, management, and analysis					
Data collection methods	18a	Plans for assessment and collection of outcome, baseline, and other trial data, including any related processes to promote data quality (eg, duplicate measurements, training of assessors) and a description of study instruments (eg, questionnaires, laboratory tests) along with their reliability and validity, if known. Reference to where data collection forms can be found, if not in the protocol.	18a	(no change)	
	18b	Plans to promote participant retention and complete follow-up, including list of any outcome data to be collected for participants who discontinue or deviate from intervention protocols.	18b	(no change)	
Data management	19	Plans for data entry, coding, security, and storage, including any related processes to promote data quality (eg, double data entry; range checks for data values). Reference to where details of data management procedures can be found, if not in the protocol.	19	(no change)	
Statistical methods	20a	Statistical methods for analyzing primary and secondary outcomes. Reference to where other details of the statistical analysis plan can be found, if not in the protocol.	20a1	Statistical methods for analyzing primary and secondary outcomes for each individual. Reference to where other details of the statistical analysis plan can be found, if not in the protocol. ***In addition for series:*** if planned, proposed methods of quantitative synthesis of individual trial data, and how heterogeneity between participants will be assessed	
			20a2	Statistical methods to account for correlation introduced by the repeated measures and crossover design of N-of-1 studies.	
	20b	Methods for any additional analyses (eg, subgroup and adjusted analyses).	20b	***For series:*** Methods for any additional analyses (eg, subgroup and adjusted analyses).	
	20c	Definition of analysis population relating to protocol non-adherence (eg, as-randomized	20c	Statistical methods to handle missing data (eg, multiple imputation, modelling). ***In addition for series:*** Definition of analysis population relating	

		analysis), and any statistical methods to handle missing data (eg, multiple imputation).		to protocol non-adherence (eg, as-randomized analysis).	
**Monitoring**					
Data monitoring	21a	Composition of Data Monitoring Committee (DMC); summary of its role and reporting structure; statement of whether it is independent from the sponsor and competing interests; and reference to where further details about its charter can be found, if not in the protocol. Alternatively, an explanation of why a DMC is not needed.	21a	(no change, SPENT commentary)	
	21b	Description of any interim analyses and stopping guidelines, including who will have access to these interim results and make the final decision to terminate the trial.	21b	(no change, SPENT commentary)	
Harms	22	Plans for collecting, assessing, reporting, and managing solicited and spontaneously reported adverse events and other unintended effects of trial interventions or trial conduct.	22	(no change, SPENT commentary)	
Auditing	23	Frequency and procedures for auditing trial conduct, if any, and whether the process will be independent from investigators and the sponsor.	23	(no change)	
**SECTION 4: ETHICS & DISSEMINATION**					
Research ethics approval	24	Plans for seeking REC/IRB approval.	24	(no change, SPENT commentary)	
Protocol amendments	25	Plans for communicating important protocol modifications (eg, changes to eligibility criteria, outcomes, analyses) to relevant parties (eg, investigators, RECs/IRBs, trial participants, trial registries, journals, regulators).	25	(no change)	
Consent or assent	26a	Who will obtain informed consent or assent from potential trial participants or authorized surrogates, and how (see item 32).	26a	(no change)	
	26b	Additional consent provisions for collection and use of participant data and biological specimens in ancillary studies, if applicable.	26b	(no change)	
Confidentiality	27	How personal information about potential and enrolled participants will be collected, shared,	27	(no change, SPENT commentary)	

		and maintained in order to protect confidentiality before, during, and after the trial.			
Declaration of interests	28	Financial and other competing interests for principal investigators for the overall trial and each study site.	28	(no change)	
Access to data	29	Statement of who will have access to the final trial data set, and disclosure of contractual agreements that limit such access for investigators.	29	(no change)	
Ancillary and post-trial care	30	Provision, if any, for ancillary and post-trial care, and for compensation to those who suffer harm from trial participation.	30	(no change)	
Dissemination policy	31a	Plans for investigators and sponsor to communicate trial results to participants, health care professionals, the public, and other relevant groups (eg, via publication, reporting in results databases, or other data-sharing arrangements), including any publication restrictions.	31a	Plans for investigators to communicate each individual's results to the participant. Plans for investigators and sponsor to communicate trial results to participants, health care professionals, the public, and other relevant groups (eg, via publication, reporting in results databases, or other data-sharing arrangements), including any publication restrictions.	
	31b	Authorship eligibility guidelines and any intended use of professional writers.	31b	(no change)	
	31c	Plans, if any, for granting public access to the full protocol, participant-level data set, and statistical code.	31c	(no change)	
**SECTION 5: APPENDICES**					
Informed consent materials	32	Model consent form and other related documentation given to participants and authorized surrogates.	32	(no change)	
Biological specimens	33	Plans for collection, laboratory evaluation, and storage of biological specimens for genetic or molecular analysis in the current trial and for future use in ancillary studies, if applicable.	33	(no change)	

Table S5. CENT 2015 checklist*; CONSORT 2010 checklist items with modifications or additions for individual or series of N-of-1 trials; empty items in the CENT 2015 column indicate no modification from the CONSORT 2010 item

Section/ Topic	CONSORT 2010 No	CONSORT 2010 Item	CENT 2015 No	CENT 2015 Item	On Page
**Title and abstract**					
	1a	Identification as a randomised trial in the title	1a	Identify as an "N-of-1 trial" in the title<br>***For series:*** Identify as "a series of N-of-1 trials" in the title	Pg. 1
	1b	Structured summary of trial design, methods, results, and conclusions (for specific guidance see CONSORT for abstracts)	1b	*For specific guidance, see CENT guidance for abstracts (table 2)*	N/A
**Introduction**					
Background and objectives	2a	Scientific background and explanation of rationale	2a.1		Pg. 2-4
			2a.2	Rationale for using N-of-1 approach	Pg. 3
	2b	Specific objectives or hypotheses	2b		Pg. 3-4
**Methods**					
Trial design	3a	Description of trial design (such as parallel, factorial) including allocation ratio	3a	Describe trial design, planned number of periods, and duration of each period (including run-in and wash out, if applicable)<br>***In addition for series:*** Whether and how the design was individualized to each participant, and explain the series design	Pg. 20-24
	3b	Important changes to methods after trial start (such as eligibility criteria), with reasons	3b		N/A
Participant(s)	4a	Eligibility criteria for participants	4a	Diagnosis or disorder, diagnostic criteria, comorbid conditions, and concurrent therapies.<br>***For series:*** Same as CONSORT item 4a	Pg. 20

	4b	Settings and locations where the data were collected	4b		Pg. 20
			4c	Whether the trial(s) represents a research study and if so, whether institutional ethics approval was obtained	Pg. 20
Interventions	5	The interventions for each group with sufficient details to allow replication, including how and when they were actually administered	5	The interventions for each period with sufficient details to allow replication, including how and when they were actually administered	Pg. 20-24
Outcomes	6a	Completely defined pre-specified primary and secondary outcome measures, including how and when they were assessed	6a.1		Pg. 2-4
			6a.2	Description and measurement properties (validity and reliability) of outcome assessment tools	Pg. 4
	6b	Any changes to trial outcomes after the trial commenced, with reasons	6b		N/A
Sample size	7a	How sample size was determined	7a		N/A
	7b	When applicable, explanation of any interim analyses and stopping guidelines	7b		N/A
Randomisation:					
Sequence generation	8a	Method used to generate the random allocation sequence	8a	Whether the order of treatment periods was randomised, with rationale, and method used to generate allocation sequence	N/A
	8b	Type of randomisation; details of any restriction (such as blocking and block size)	8b	When applicable, type of randomisation; details of any restrictions (such as pairs, blocking)	N/A
			8c	Full, intended sequence of periods	N/A
Allocation concealment mechanism	9	Mechanism used to implement the random allocation sequence (such as sequentially numbered containers), describing any steps taken to conceal the sequence until interventions were assigned	9		N/A
Implementation	10	Who generated the random allocation sequence, who enrolled participants, and who assigned participants to interventions	10		N/A

Blinding	11a	If done, who was blinded after assignment to interventions (for example, participants, care providers, those assessing outcomes) and how	11a		N/A
	11b	If relevant, description of the similarity of interventions	11b		N/A
Statistical methods	12a	Statistical methods used to compare groups for primary and secondary outcomes	12a	Methods used to summarize data and compare interventions for primary and secondary outcomes	Pg. 20-24
	12b	Methods for additional analyses, such as subgroup analyses and adjusted analyses	12b	***For series:*** If done, methods of quantitative synthesis of individual trial data, including subgroup analyses, adjusted analyses, and how heterogeneity between participants was assessed, (for specific guidance on reporting syntheses of multiple trials, please consult the PRISMA Statement)	N/A
			12c	Statistical methods used to account for carryover effect, period effects, and intra-subject correlation	N/A
**Results**					
Participant flow (a diagram is strongly recommended)	13a	For each group, the numbers of participants who were randomly assigned, received intended treatment, and were analysed for the primary outcome	13a.1	Number and sequence of periods completed, and any changes from original plan with reasons	N/A
			13a.2	***For series:*** The number of participants who were enrolled, assigned to interventions, and analysed for the primary outcome	N/A
	13b	For each group, losses and exclusions after randomisation, together with reasons	13c	***For series:*** losses or exclusions of participants after treatment assignment, with reasons, and period in which this occurred, if applicable	N/A
Recruitment	14a	Dates defining the periods of recruitment and follow-up	14a		N/A
	14b	Why the trial ended or was stopped	14b	Whether any periods were stopped early and/or whether trial was stopped early, with reason(s).	N/A
Baseline data	15	A table showing baseline demographic and clinical characteristics for each group	15†		N/A
Numbers analysed	16	For each group, number of participants (denominator) included in each analysis and	16	For each intervention, number of periods analysed.	N/A

		whether the analysis was by original assigned groups		***In addition for series:*** if quantitative synthesis was performed, number of trials for which data were synthesized	
Outcomes and estimation	17a	For each primary and secondary outcome, results for each group, and the estimated effect size and its precision (such as 95% confidence interval)	17a.1	For each primary and secondary outcome, results for each period; an accompanying figure displaying the trial data is recommended.	Pg. 4-11, Pg. 36-44 Pg. 46
			17a.2	For each primary and secondary outcome, the estimated effect size and its precision (such as 95% confidence interval)<br>***In addition for series:*** if quantitative synthesis was performed, group estimates of effect and precision for each primary and secondary outcome	N/A
	17b	For binary outcomes, presentation of both absolute and relative effect sizes is recommended	17b		N/A
Ancillary analyses	18	Results of any other analyses performed, including subgroup analyses and adjusted analyses, distinguishing pre-specified from exploratory	18	Results of any other analyses performed, including assessment of carryover effects, period effects, intra-subject correlation<br>***In addition for series:*** If done, results of subgroup or sensitivity analyses	N/A
Harms	19	All important harms or unintended effects in each group (for specific guidance see CONSORT for harms)	19	All harms or unintended effects for each intervention. *(for specific guidance see CONSORT for harms)*	N/A
**Discussion**					
Limitations	20	Trial limitations, addressing sources of potential bias, imprecision, and, if relevant, multiplicity of analyses	20		Pg. 18-19
Generalisability	21	Generalisability (external validity, applicability) of the trial findings	21		Pg. 18-19
Interpretation	22	Interpretation consistent with results, balancing benefits and harms, and considering other relevant evidence	22		Pg. 18-19
**Other information**					

Registration	23	Registration number and name of trial registry	23	Pg. 20
Protocol	24	Where the full trial protocol can be accessed, if available	24	N/A
Funding	25	Sources of funding and other support (such as supply of drugs), role of funders	25	Pg. 24

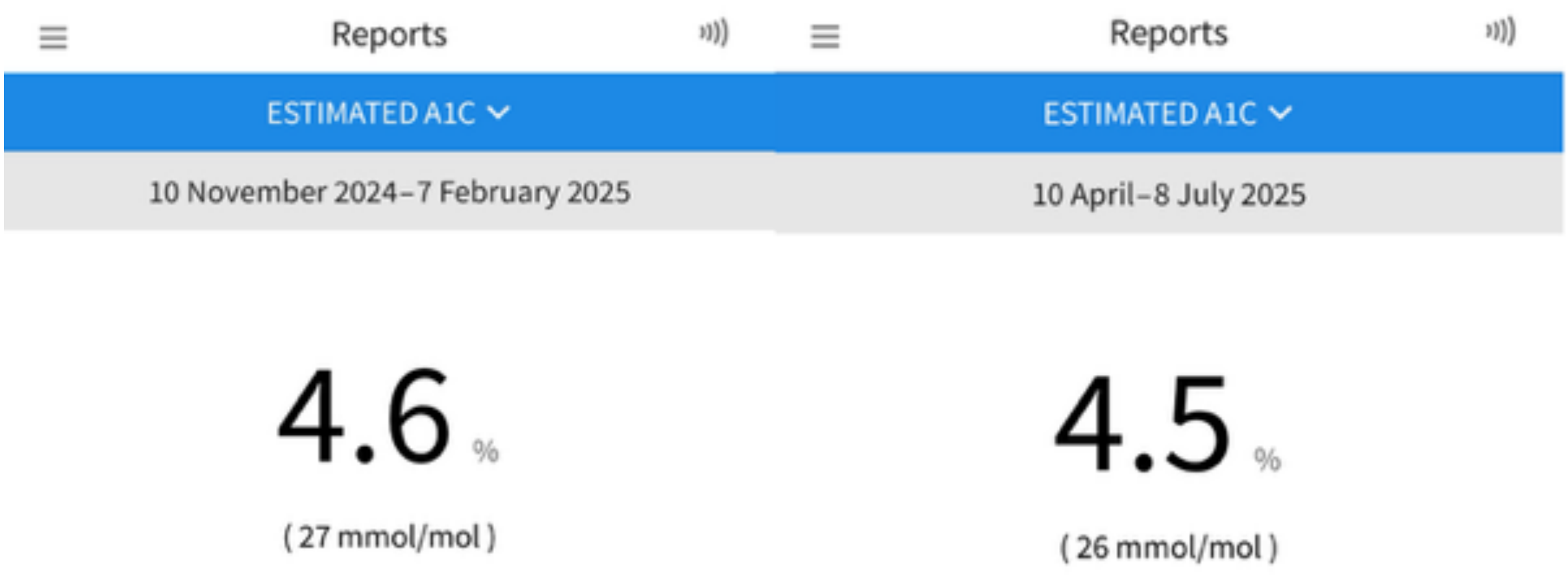

Fig. 1. Estimated HbA1C (mmol/mol) from CGM (Abbott Freestyle Libre).

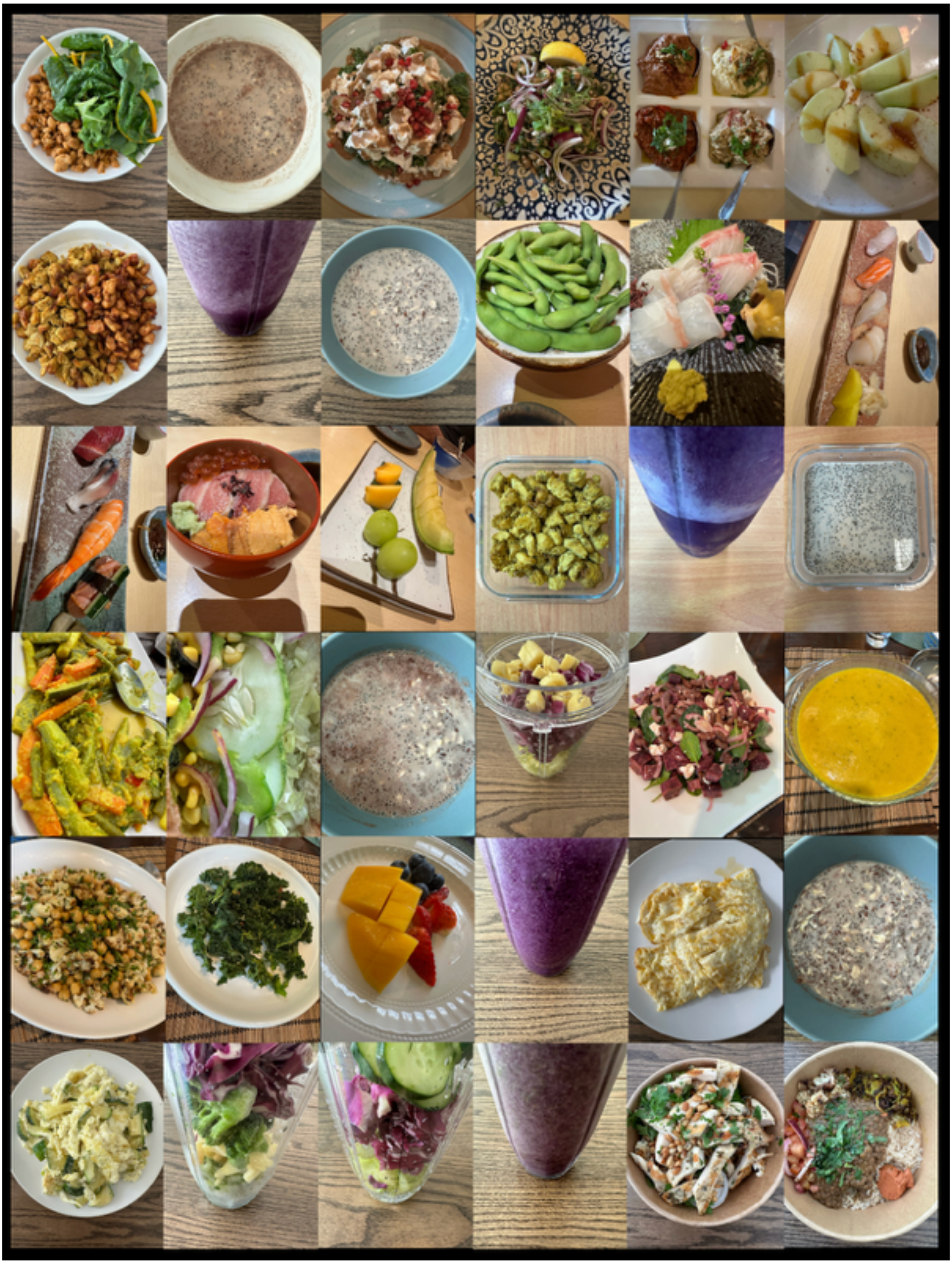

Fig. 2. DELTA001’s dietary regimen from Oct 1-7 2024.

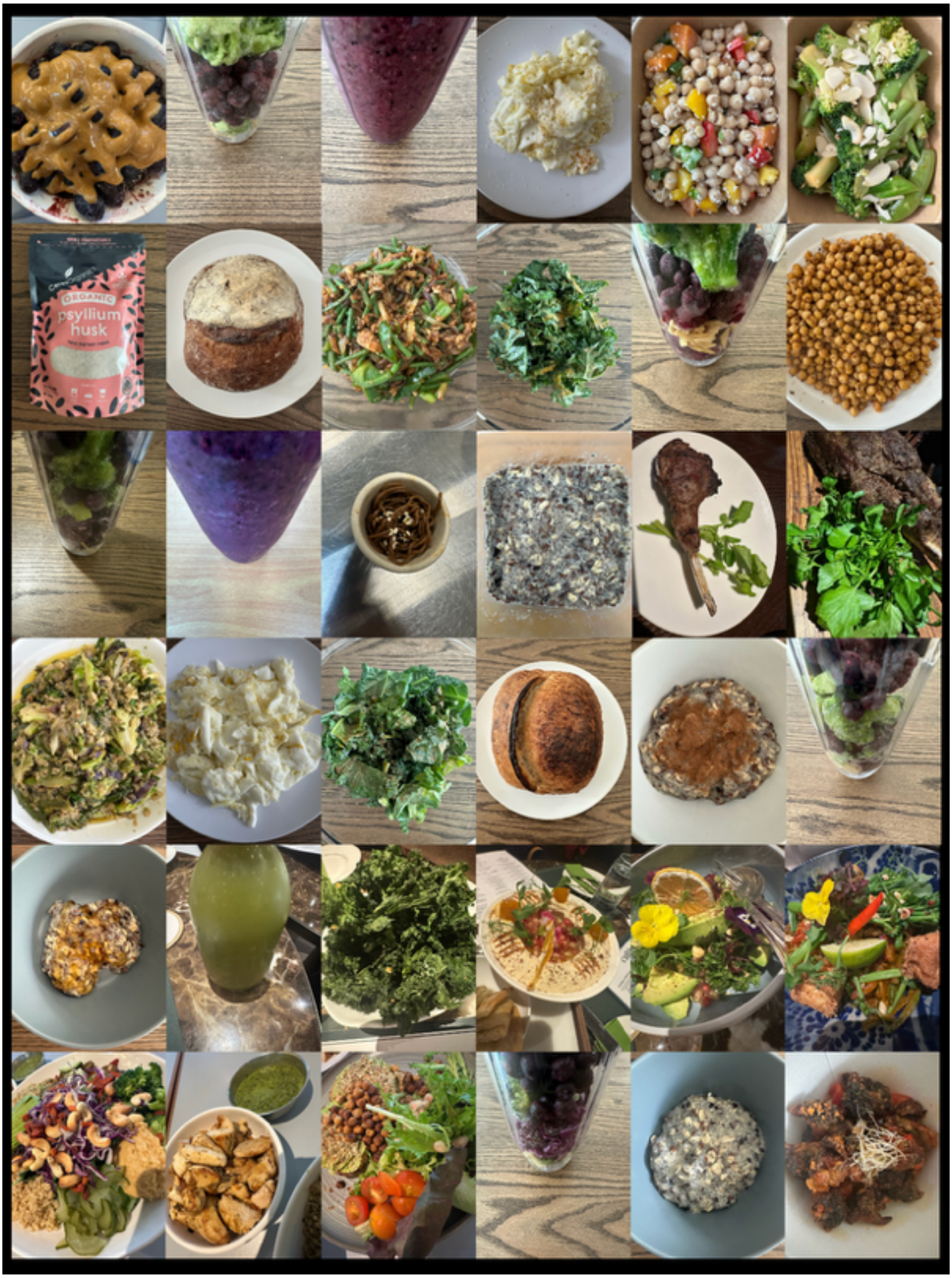

Fig. 3. DELTA001's dietary regimen from Dec 1-7 2024.

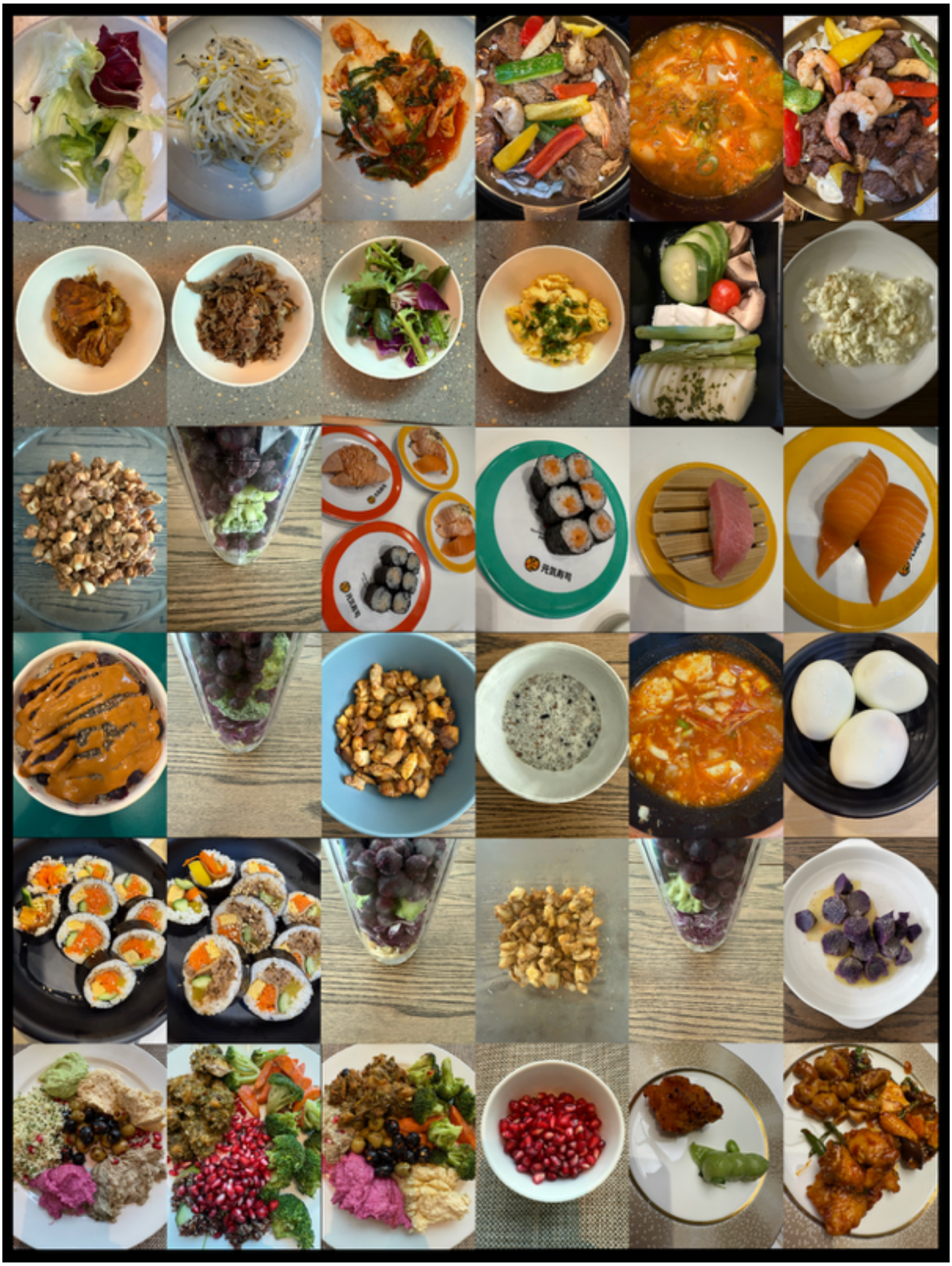

Fig. 4. DELTA001’s dietary regimen from Jan 1-7 2025.

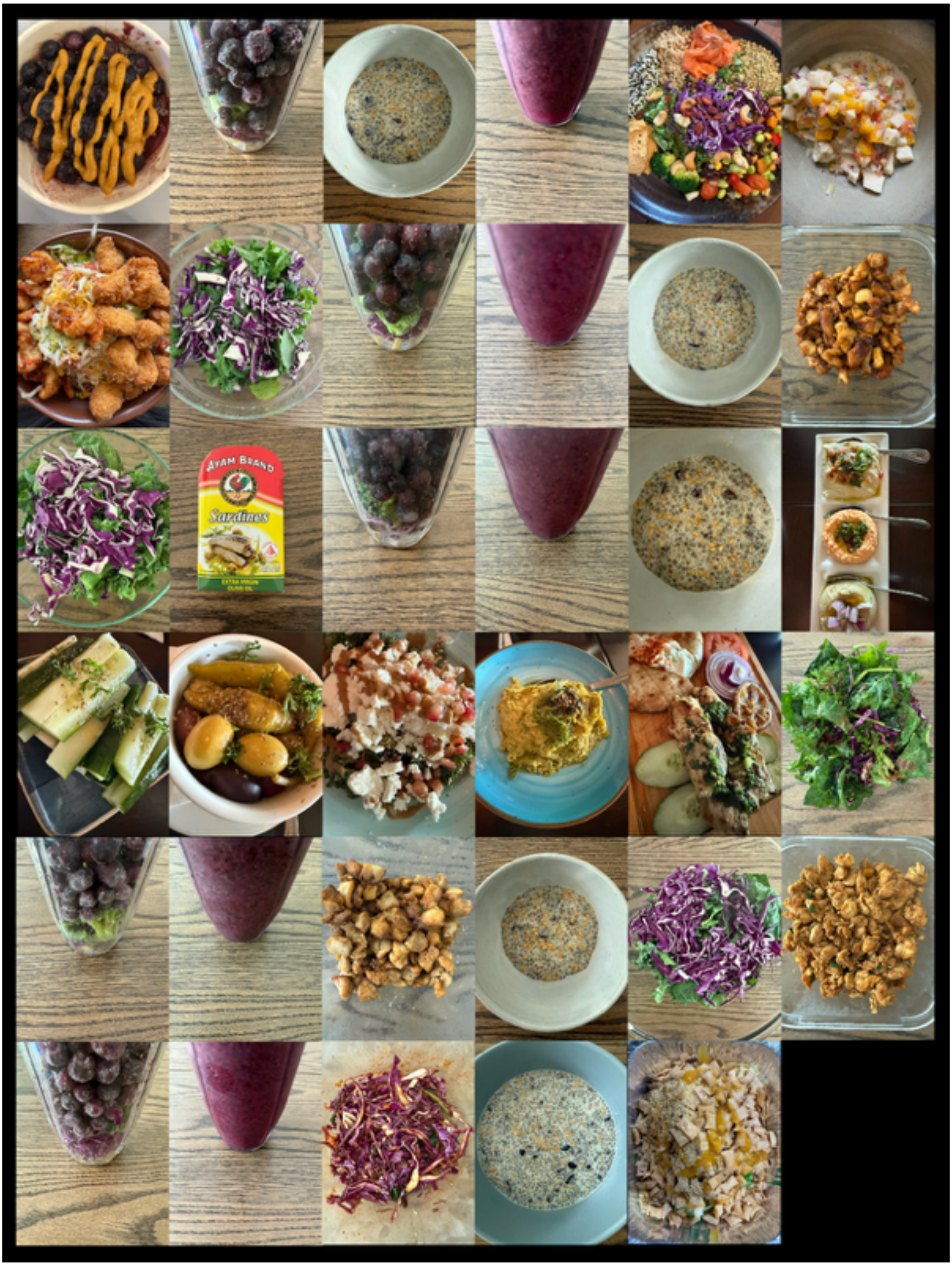

Fig. 5. DELTA001’s dietary regimen from Jan 1-7 2025.

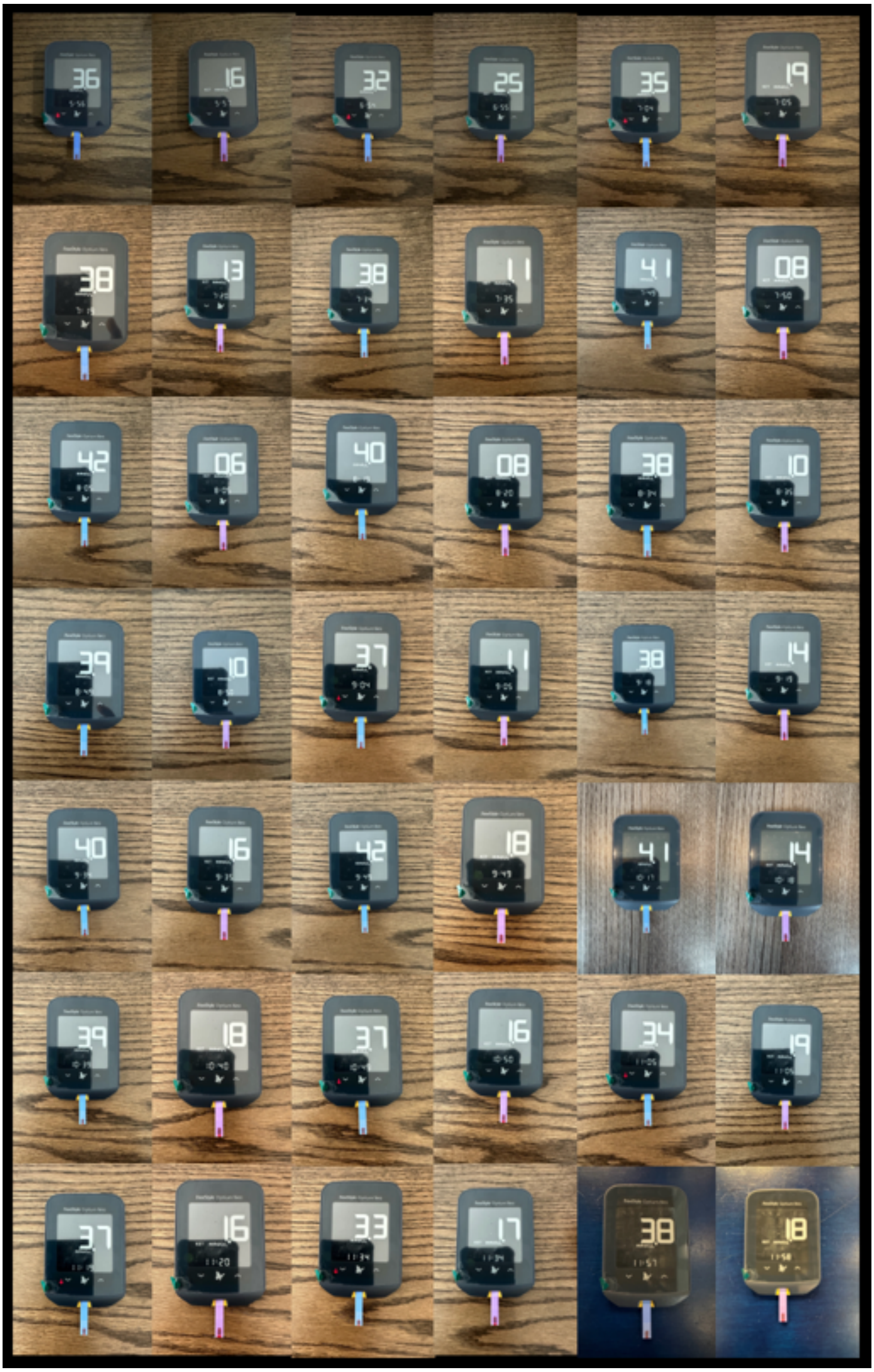

Fig. 6. Measured blood ketone and glucose levels for the 48-hour fasting strength training regimen on Sep 20 2024.

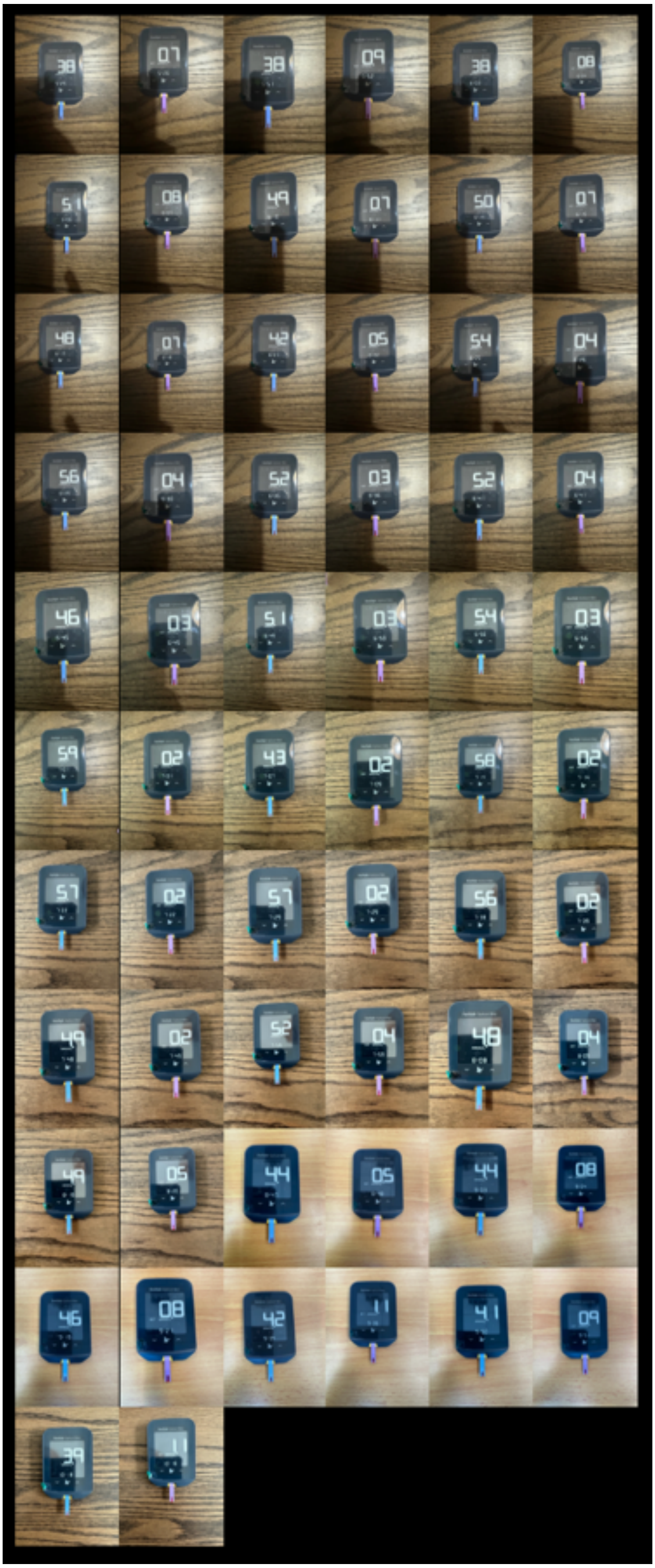

Fig. 7. Measured blood ketone and glucose levels for the 48-hour fasting strength training regimen on Oct 10 2024.

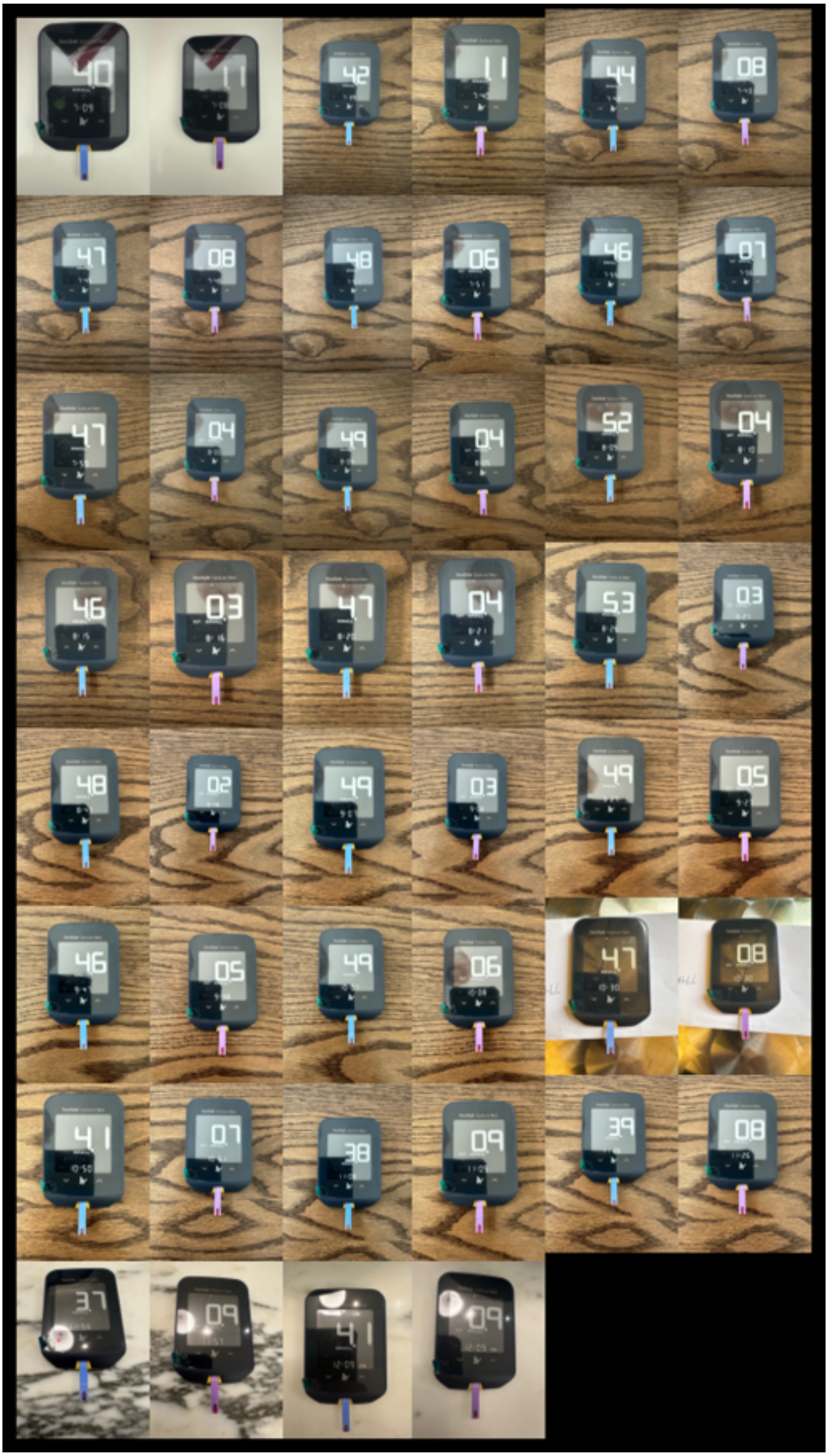

Fig. 8. Measured blood ketone and glucose levels for the 48-hour fasting strength training regimen on Oct 25 2024.

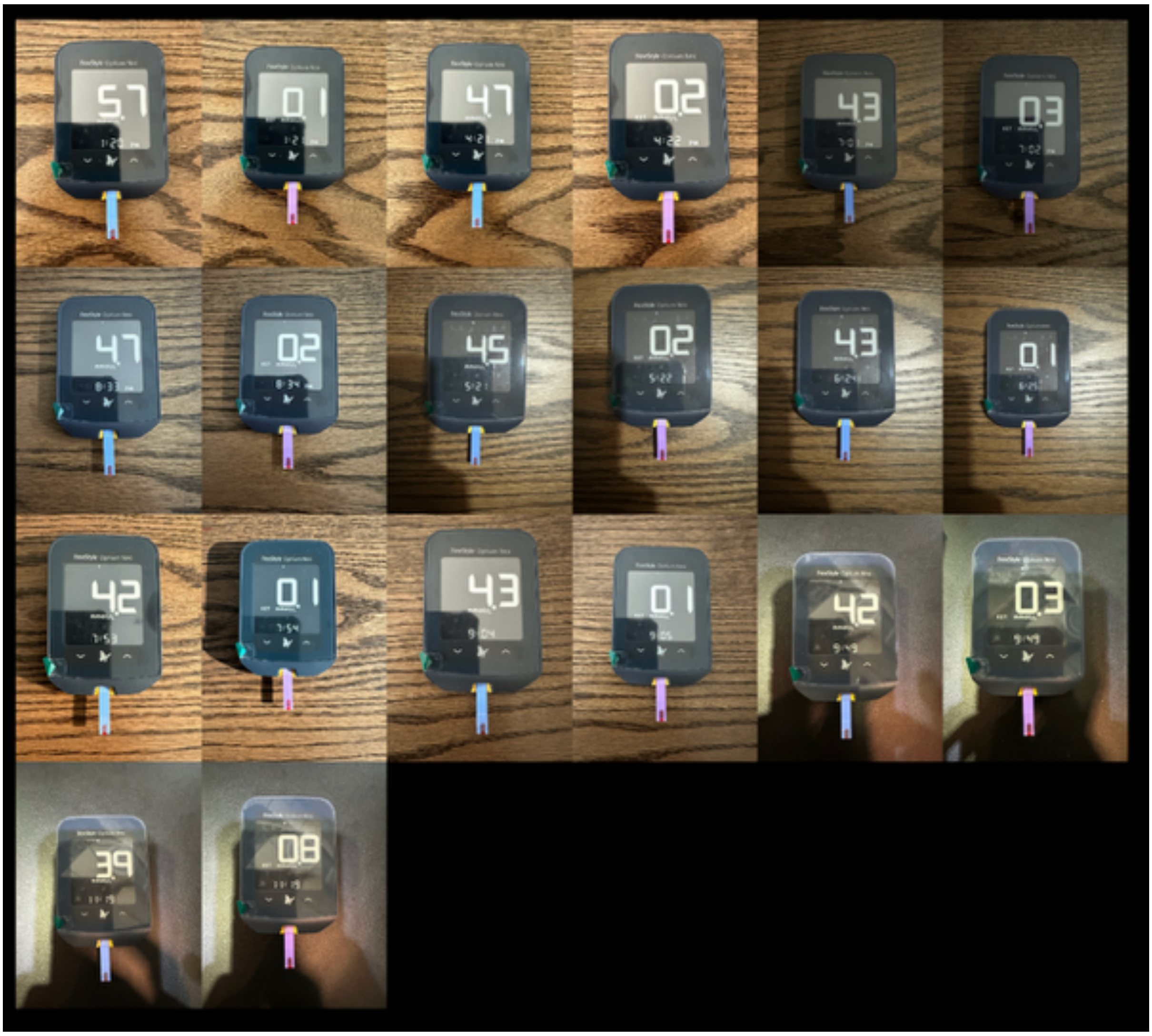

Fig. 9. Measured blood ketone and glucose levels for the OMAD ketosis entry regimen from Oct 27-28, 2024.

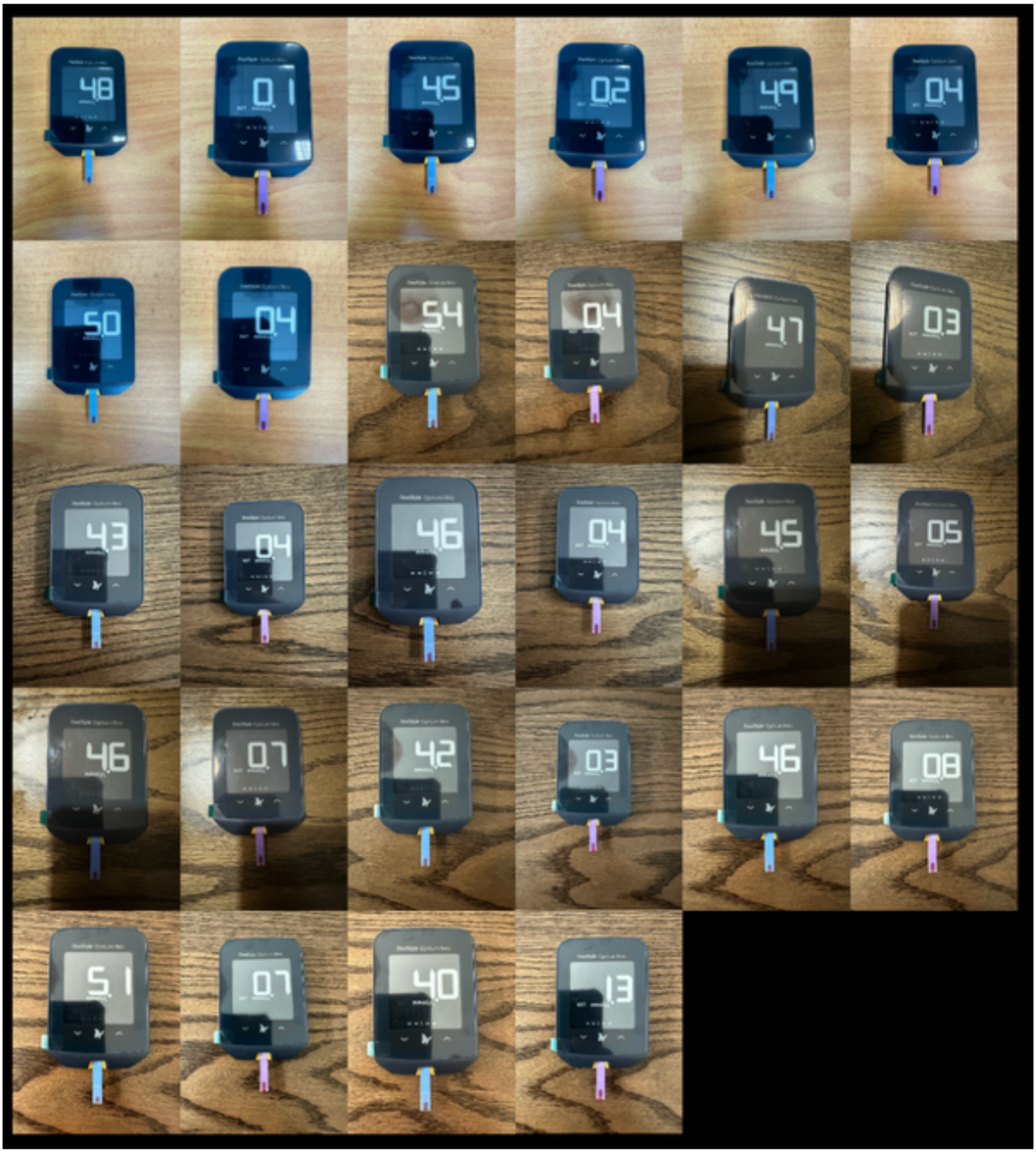

Fig. 10. Measured blood ketone and glucose levels for the OMAD ketosis entry regimen from Nov 19-20, 2024.

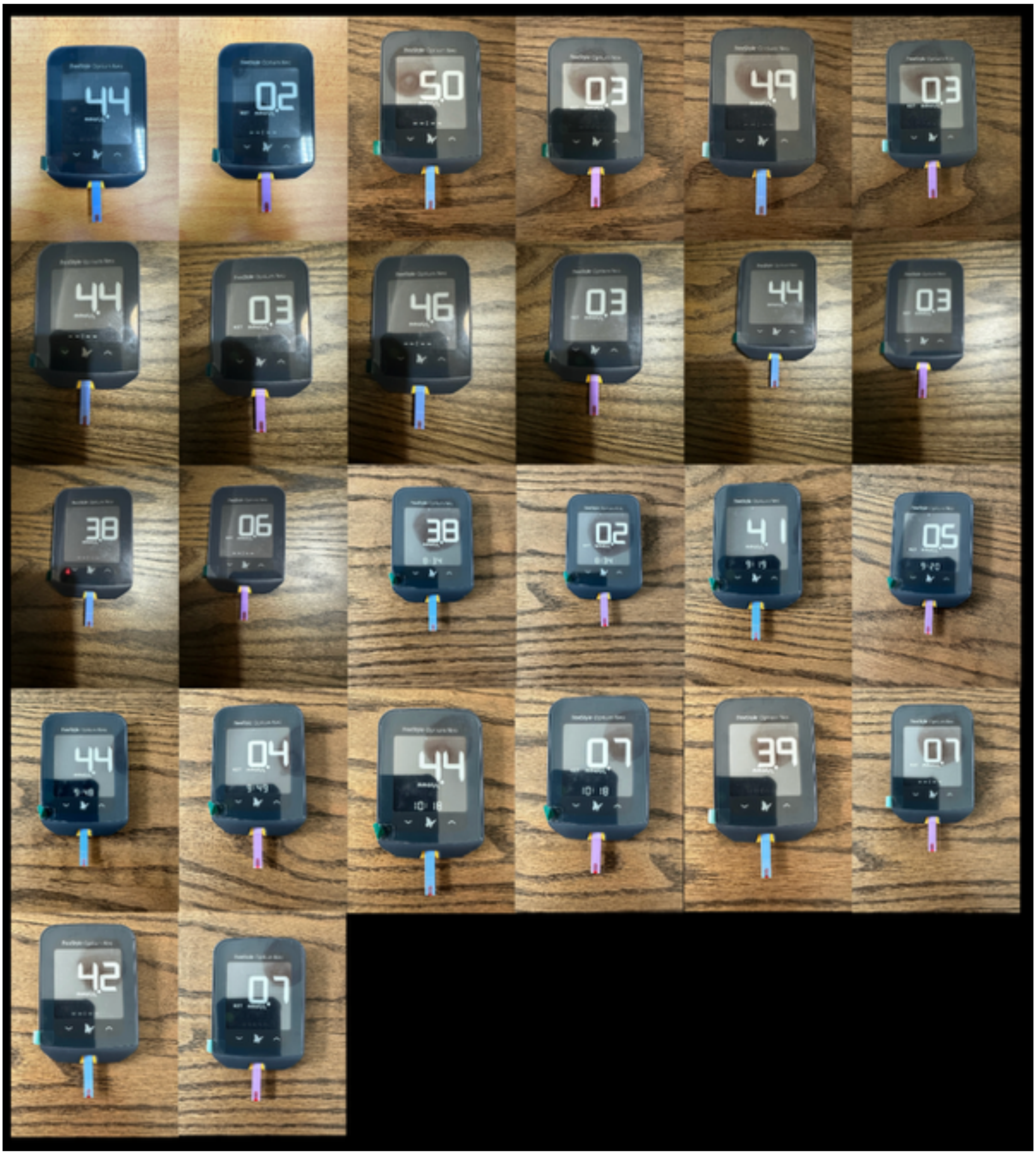

Fig. 11. Measured blood ketone and glucose levels for the OMAD ketosis entry regimen from Nov 26-27, 2024.

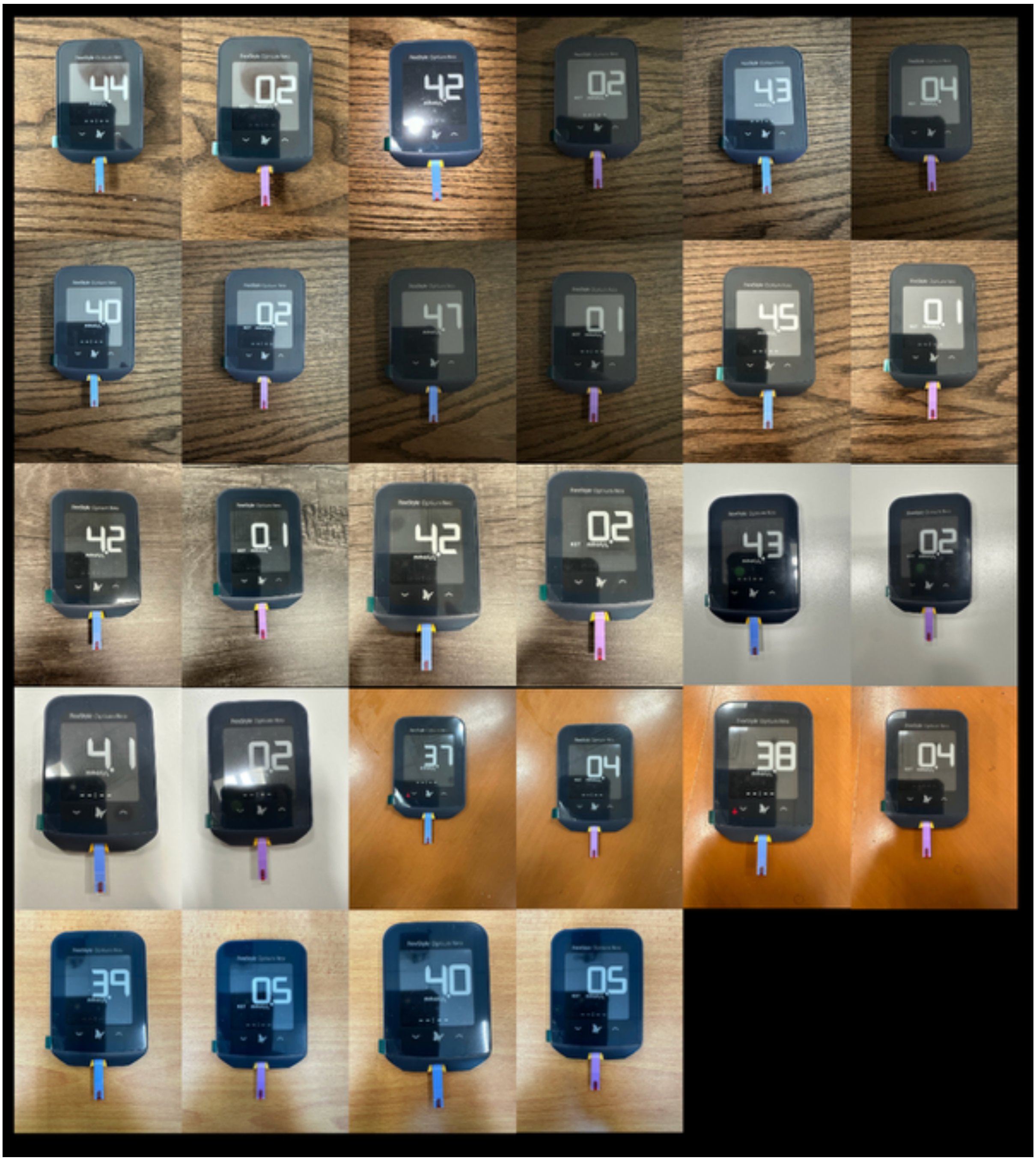

Fig. 12. Measured blood ketone and glucose levels for the OMAD ketosis entry regimen from Dec 9-10, 2024.

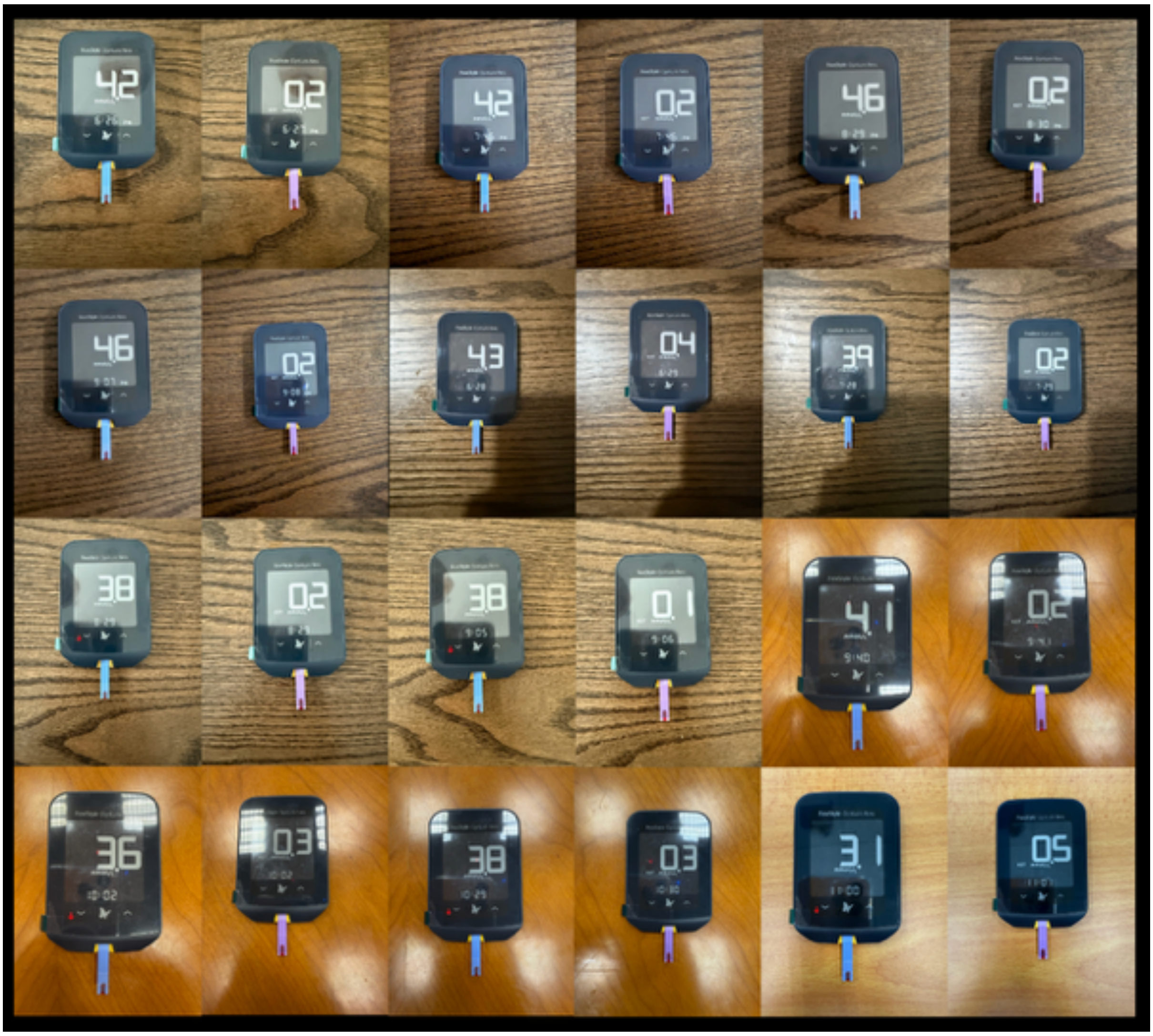

Fig. 13. Measured blood ketone and glucose levels for the OMAD ketosis entry regimen from Feb 3-4, 2025.

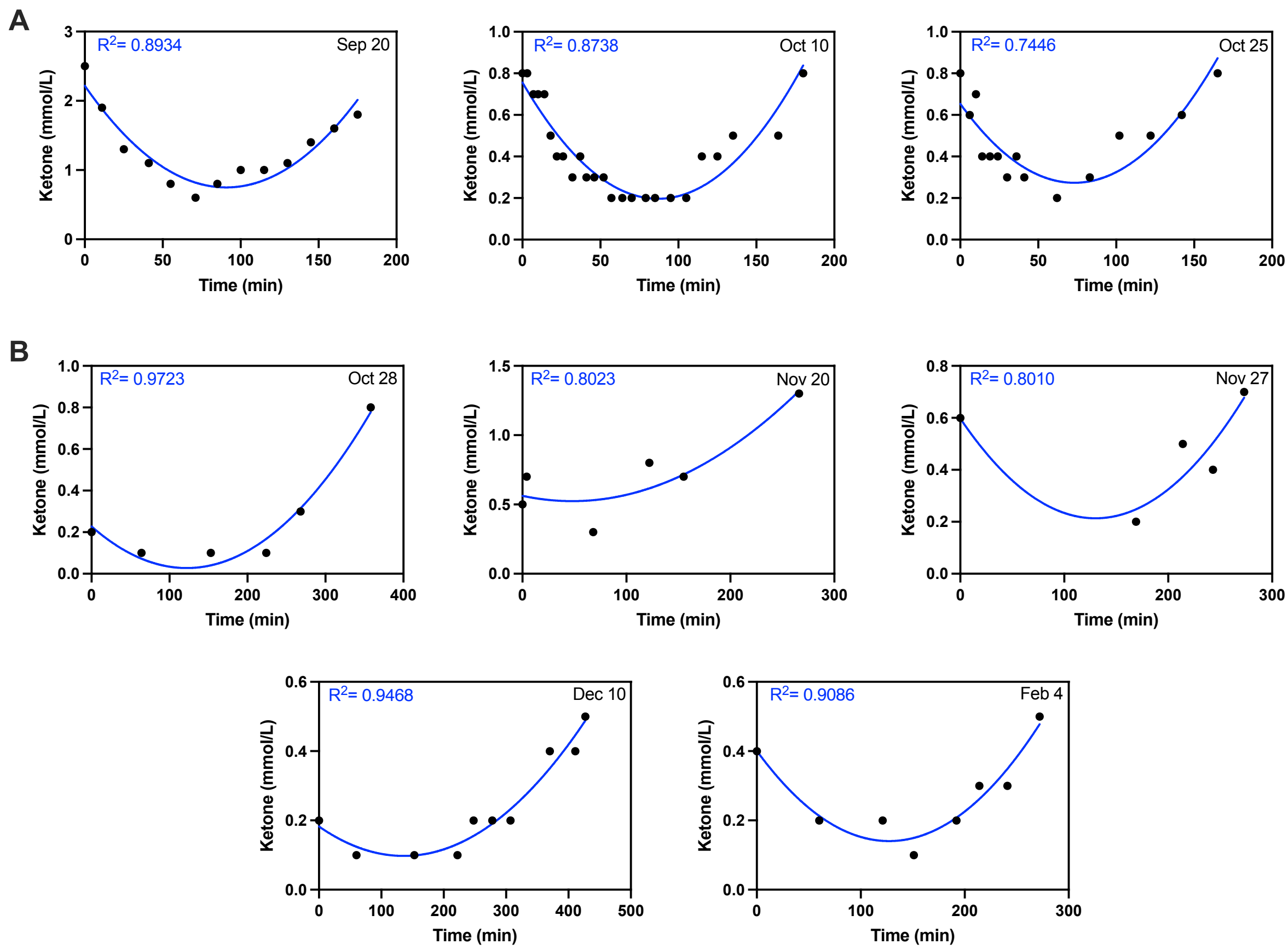

Fig. 14. Second-order regression analysis of ketone dynamics. **(A)** The 48-hour fasting strength training regimen. Measurements taken from exercise start to ketotic recovery were used for regression analysis. **(B)** The OMAD ketosis entry regimen. Measurements taken on day 2 of the regimen were used for regression analysis.

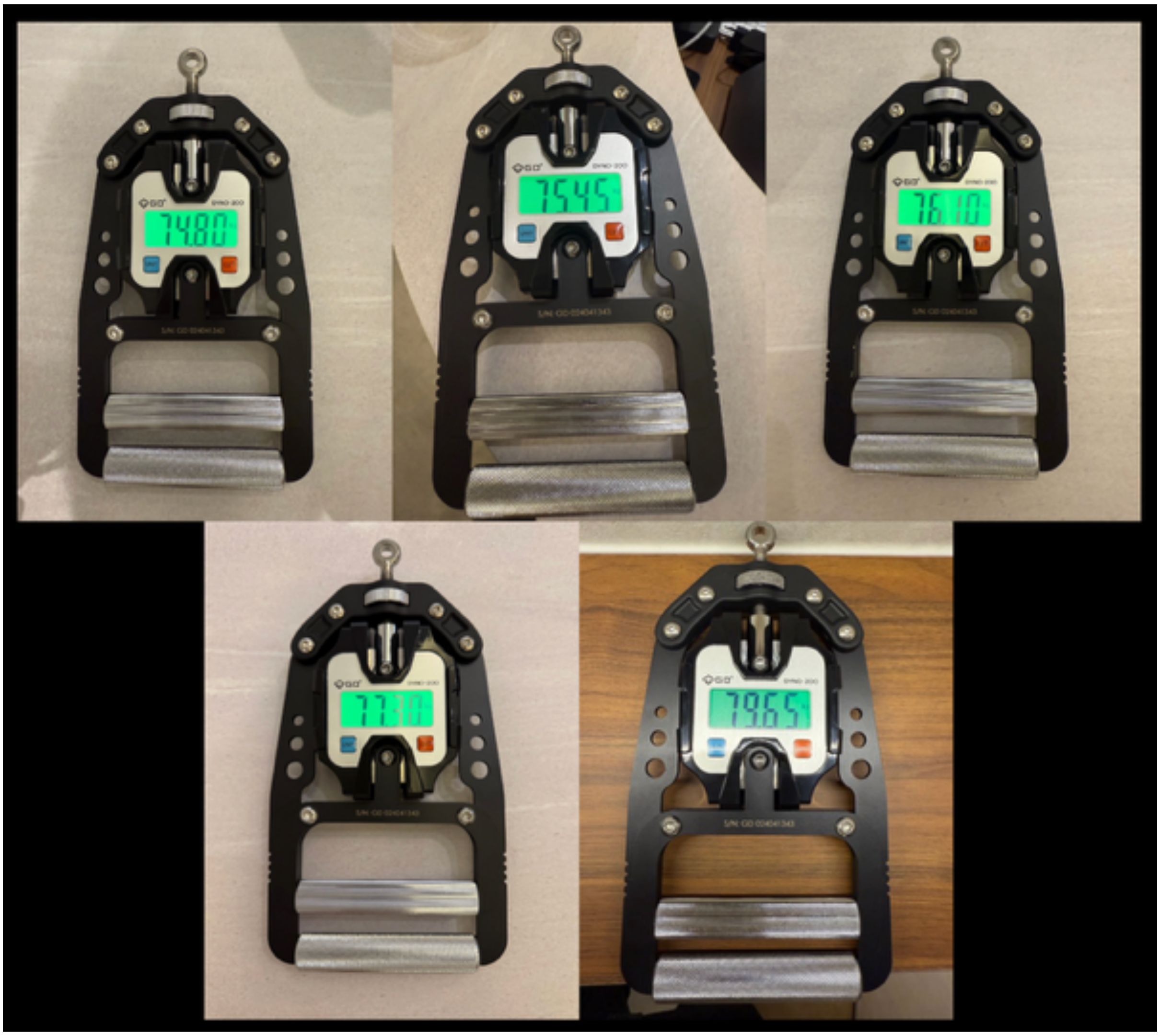

Fig. 15. Grip strength (dominant-hand) measured using an industrial grade grip strength meter (GD DYNO 200) on Feb 17, 18, and 28 2025.

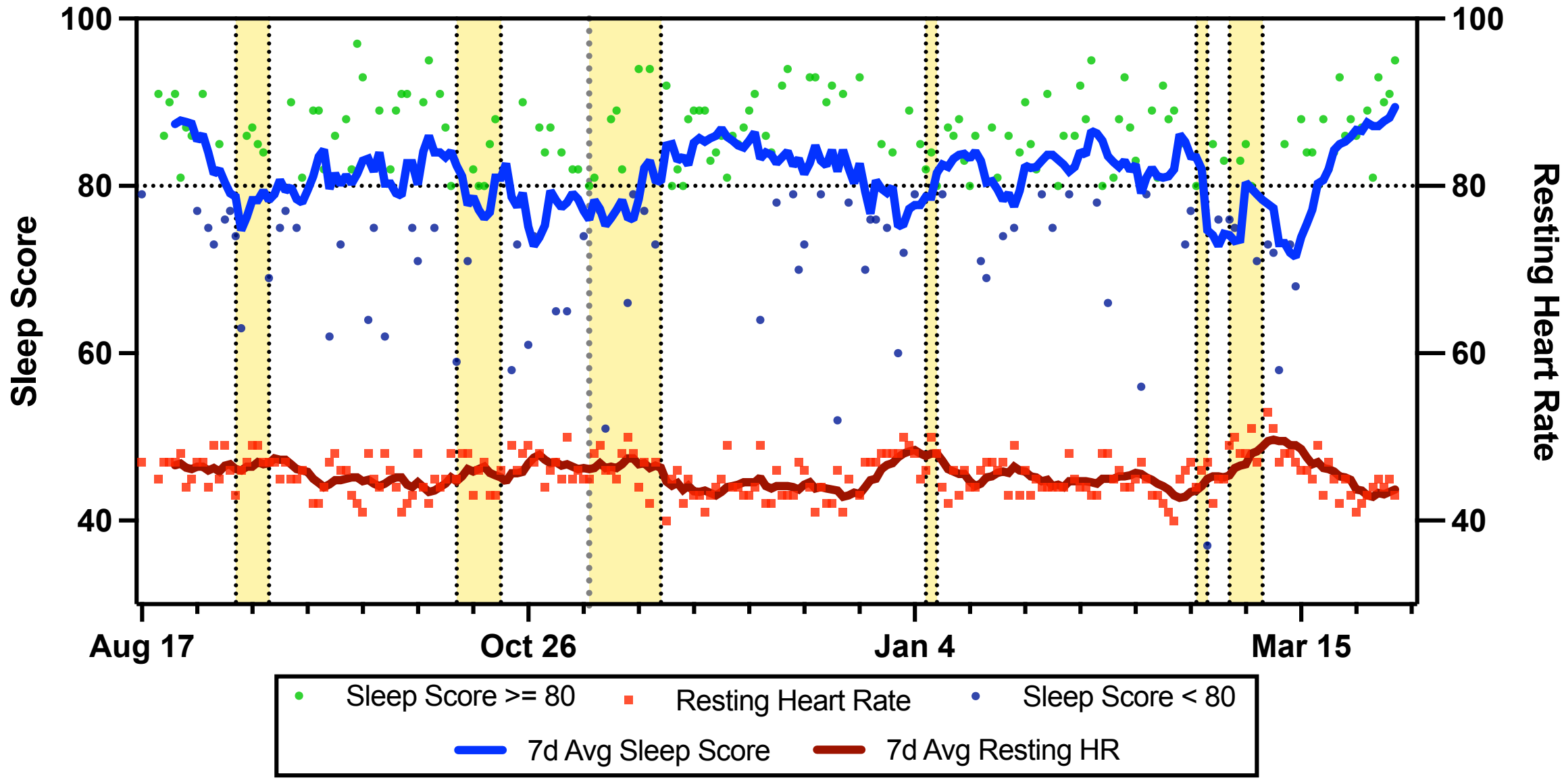

Fig. 16. Longitudinal tracking of Garmin sleep score compared to trends in resting heart rate (RHR). The lines indicate a 7-day rolling average of RHR (red) and sleep score (blue). Yellow shaded regions indicate periods of overseas travel. Source data can be found in Supplementary Data 1.

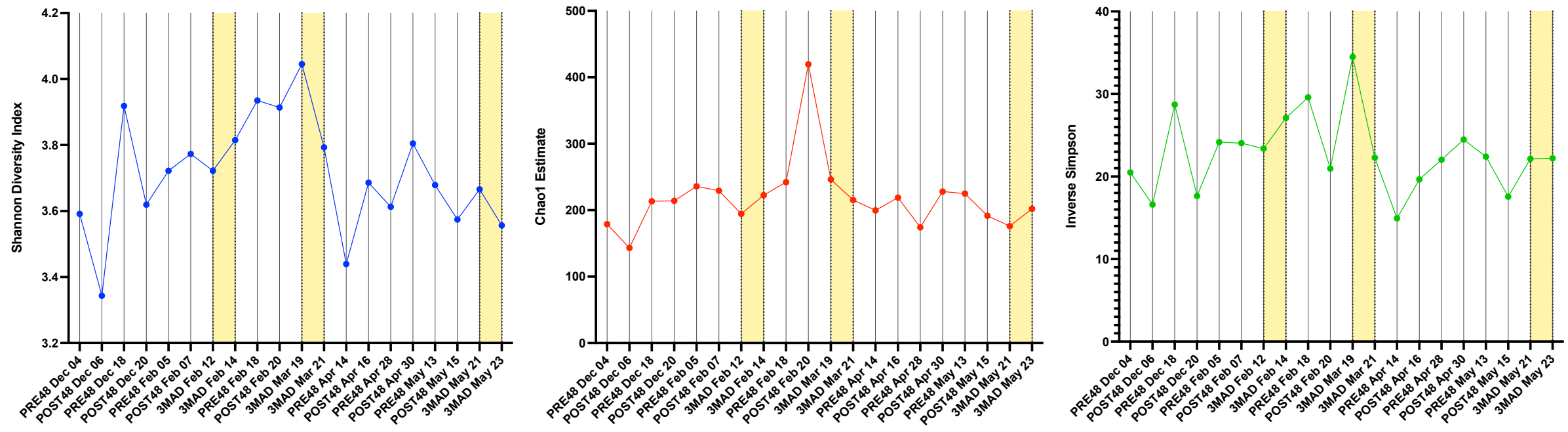

Fig. 17. Microbiome diversity measured using Shannon, Chao1 and Inverse Simpson over the course of the study – with PRE48 indicating samples that were taken before a 48-hour fast, POST48 indicating samples that were taken after a 48-hour fast and 3MAD indicating samples taken during the intervention period of a 3MAD week (highlighted in yellow). Source data can be found in Supplementary Data 2.

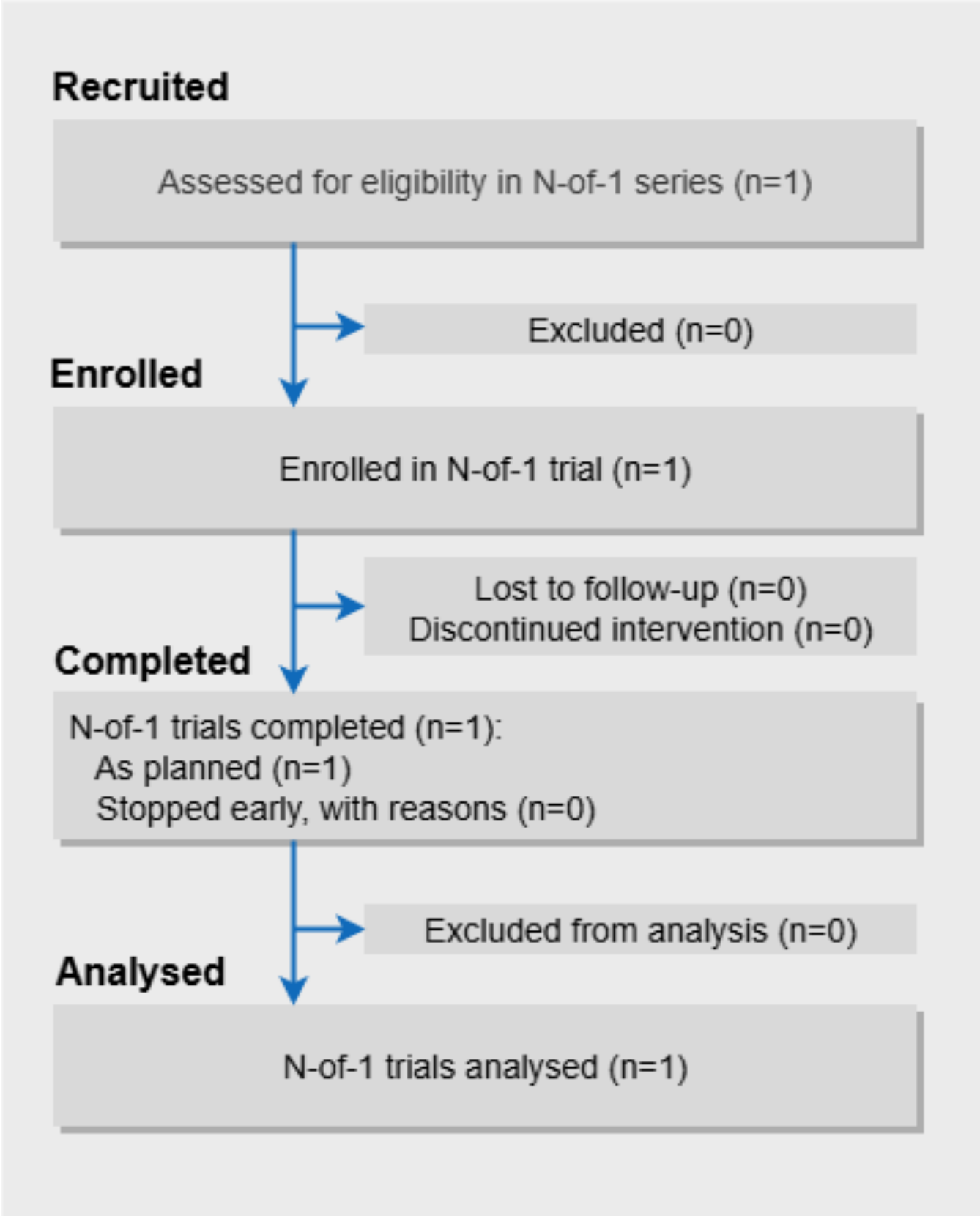

Fig. 18. CONSORT extension for reporting N-of-1 trials (CENT) participant flow diagram illustrating flow of participant in N-of-1 trial. There were no dropouts in this study, and the study's sole participant remained in the trial until its completion.
