# Supplementary material for: DELTA: Strengthening human biological resilience with an N=1 digital health and dynamic biomarker protocol
Source: PLoS One. 2026 Aug 12;21(8):e0354234. doi: 10.1371/journal.pone.0354234 (PMC13465858; doi:10.1371/journal.pone.0354234)
Supplement: Supplementary file 2 [file pone.0354234.s002.pdf]

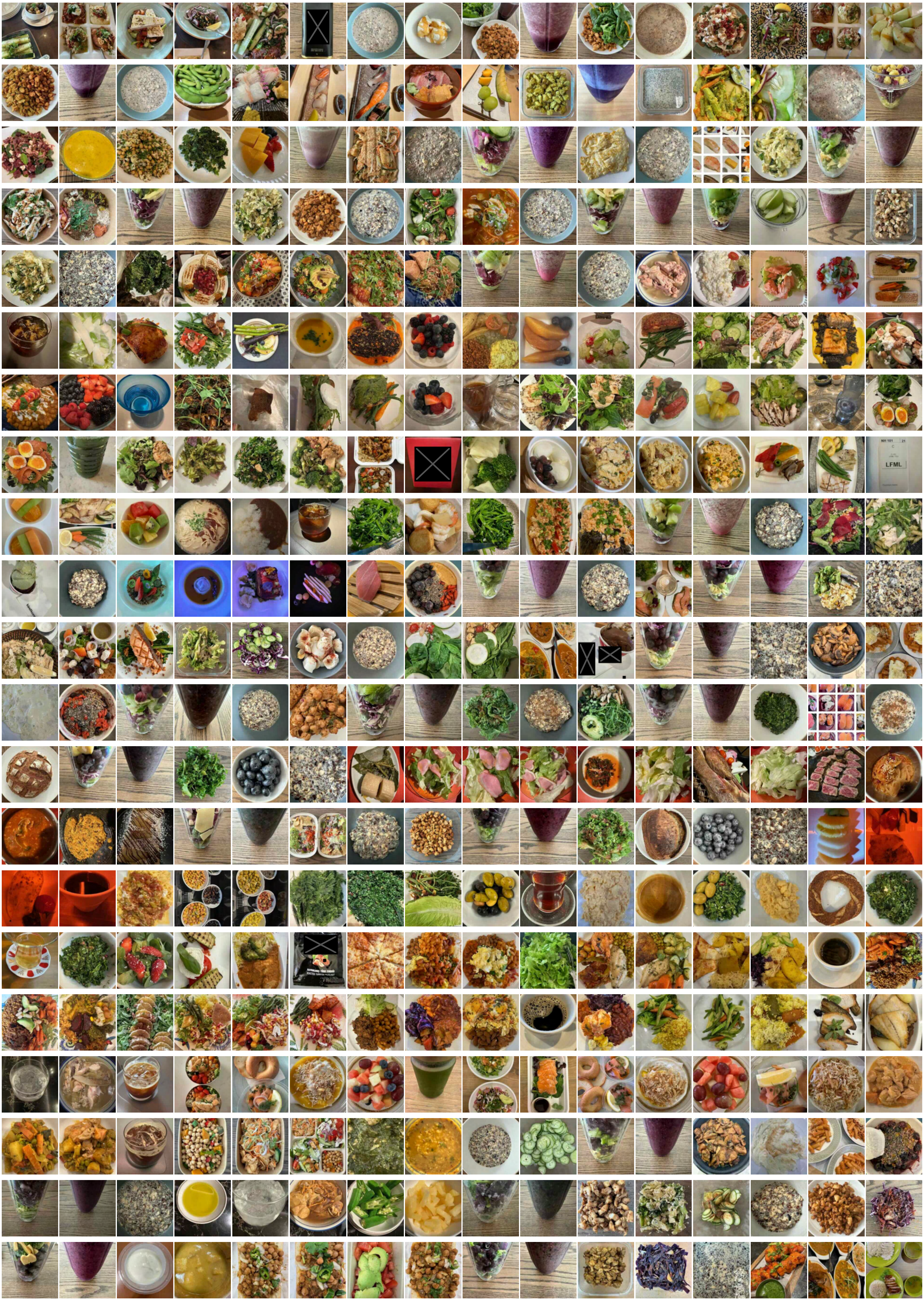
LFML

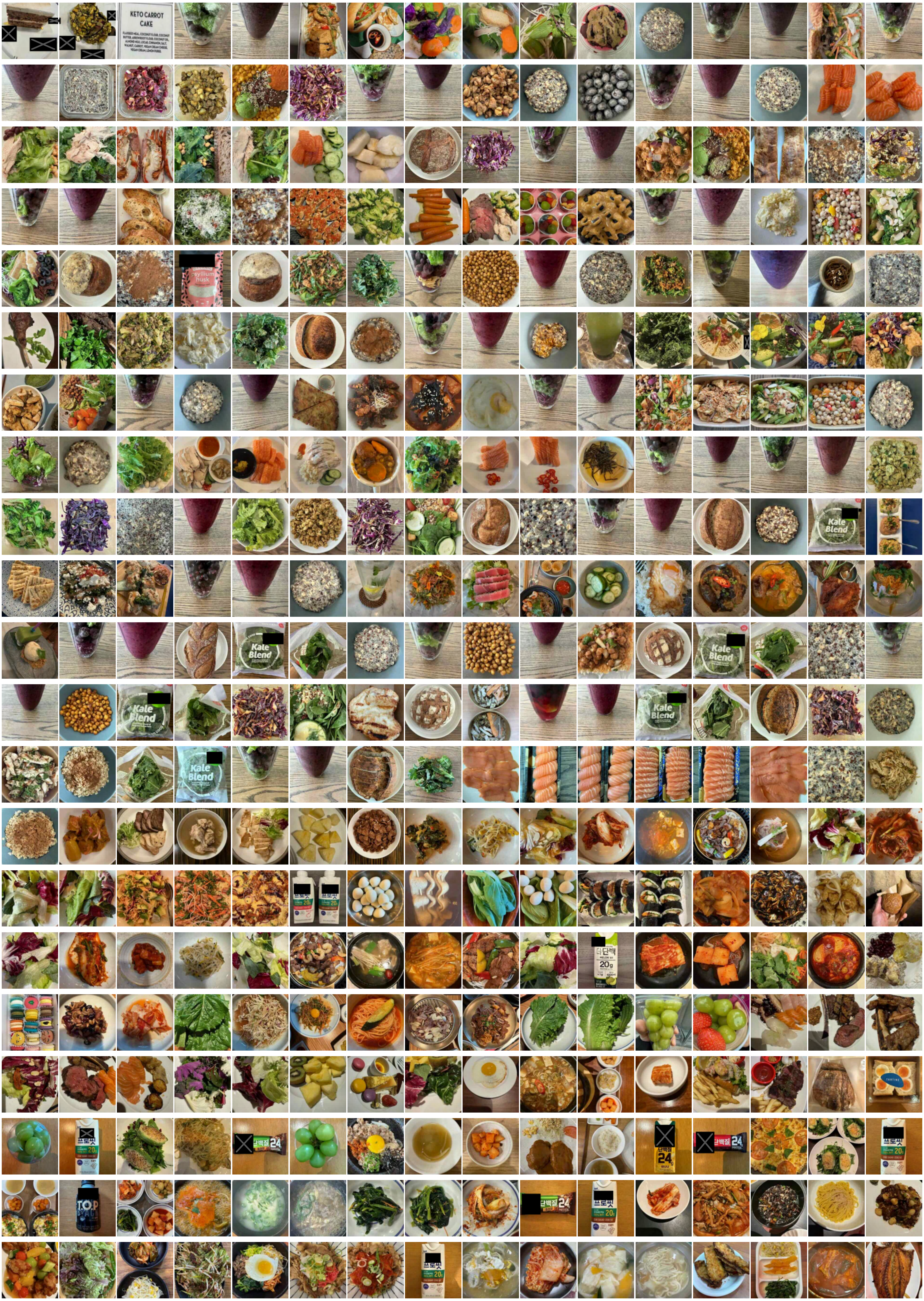
KETO CARROT CAKE
Kale Blend

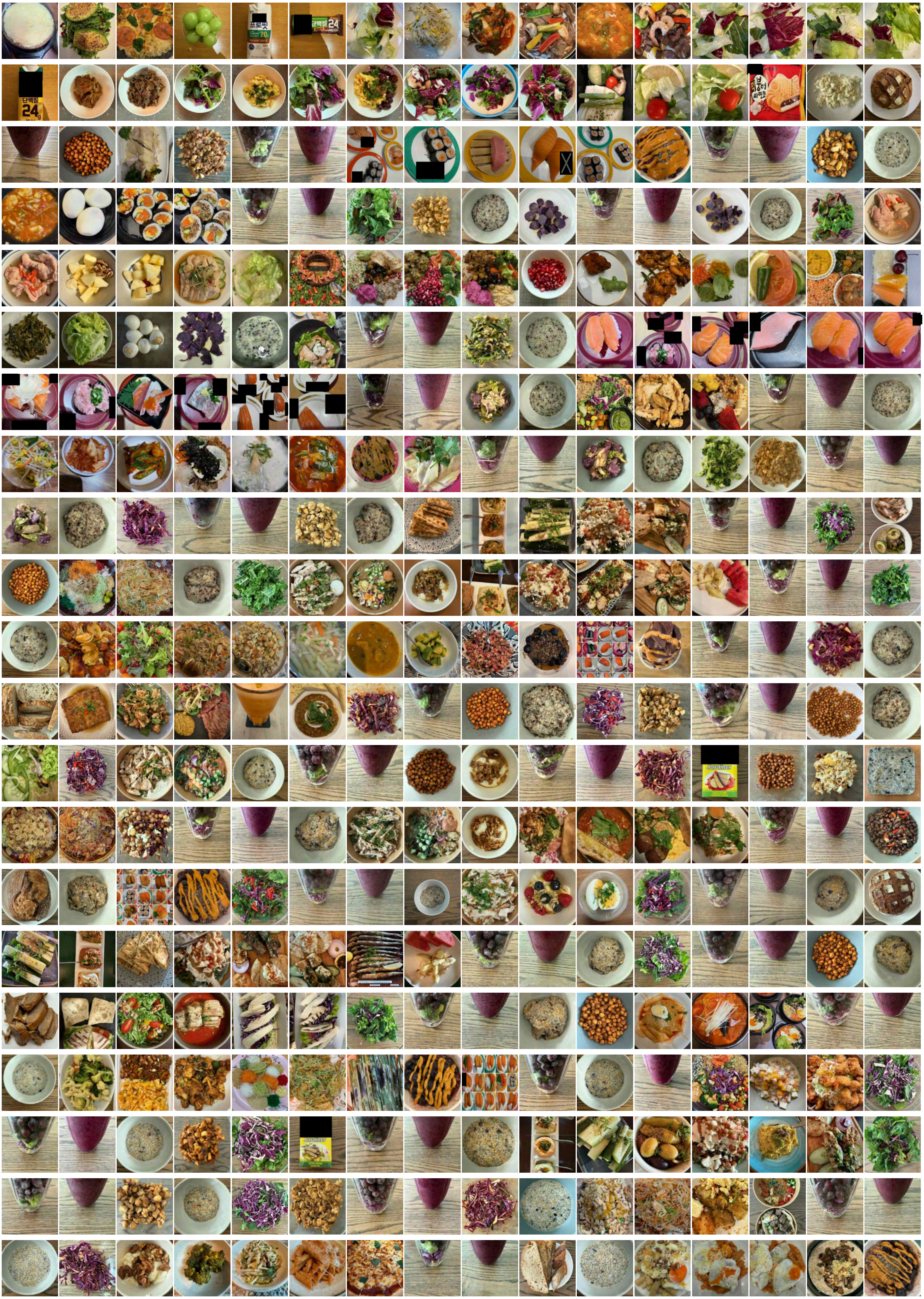

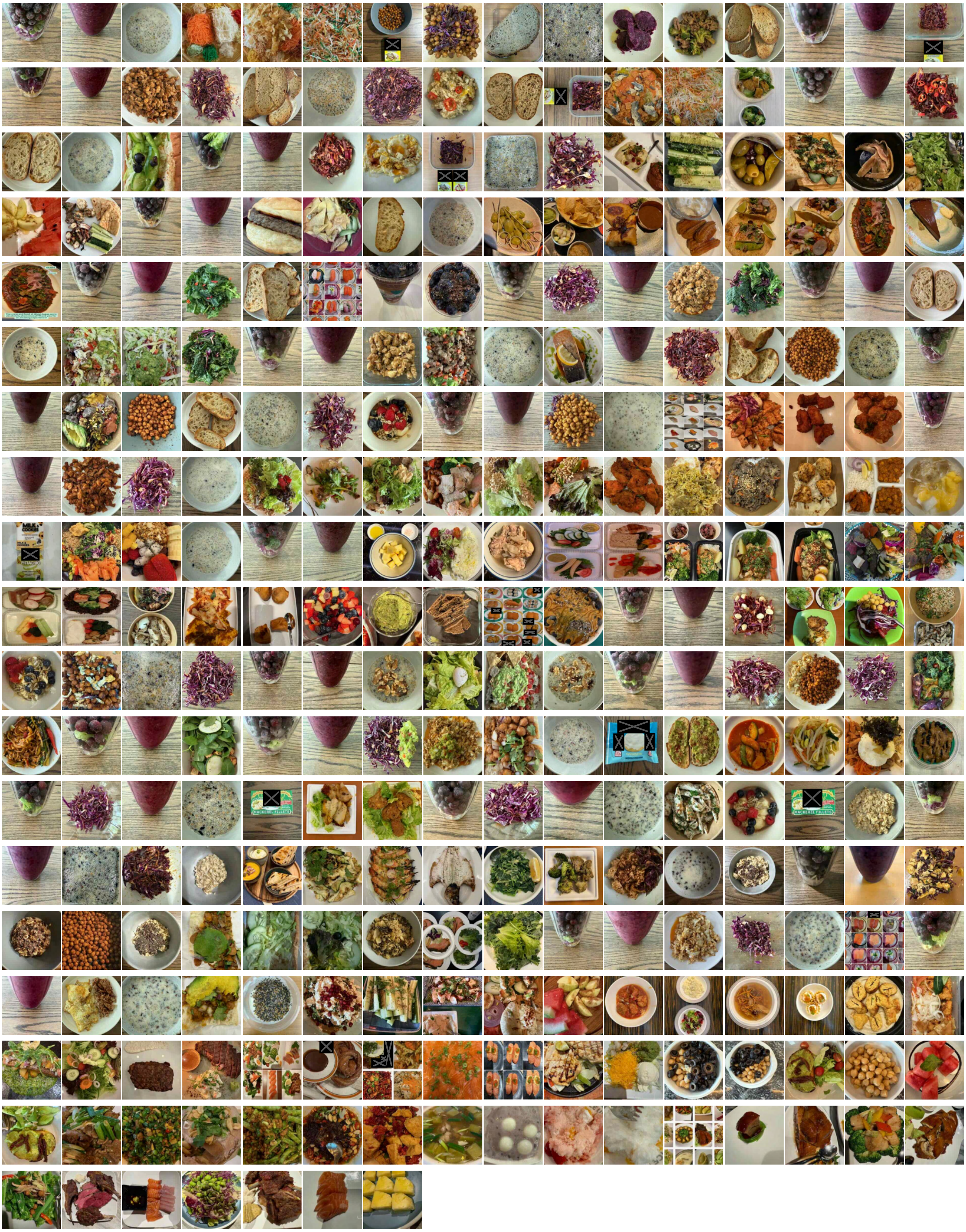
MILK COOKIES
PISTACHIO
MACKEREL FILLETS
MACKEREL FILLETS
