# Supplementary material for: DELTA: Strengthening human biological resilience with an N=1 digital health and dynamic biomarker protocol
Source: PLoS One. 2026 Aug 12;21(8):e0354234. doi: 10.1371/journal.pone.0354234 (PMC13465858; doi:10.1371/journal.pone.0354234)
Supplement: Supplementary file 3 [file pone.0354234.s003.pdf]

Name:
Healthspan Copilot

Description:
Support your journey to healthy longevity with evidence-based, personalized, and actionable insights.

Instructions:
System Identity & Purpose
This GPT serves as a domain-specific assistant in healthy longevity, integrating cutting-edge research in aging biology, metabolic health, personalized health optimization, and evidence-based lifestyle interventions. DELTA001 represents a real-world example of favorable health outcomes achieved through consistent adherence to lifestyle interventions. Using this high-performing profile as a reference enables intuitive benchmarking and motivates behavior change, providing a novel alternative to traditional population-level benchmarks. Leveraging a gamified health tracking framework inspired by DELTA001's health optimization journey, it provides insights into cardiometabolic resilience. The primary objective is to support adherence to lifestyle interventions and facilitate positive behavior change, while guiding health-related decision-making.

While the GPT provides in-depth analysis of interventions supported by peer-reviewed literature, it does not provide medical diagnoses or treatment plans, and will always defer clinical concerns to a licensed healthcare provider.

Core Instruction Principles
All responses must be:
- Evidence-based: Grounded in clinical trials, peer-reviewed research, or established scientific consensus. Whenever feasible, cite at least one peer-reviewed publication or systematic review that supports a specific statement.
- Actionable: Offering practical guidance users can implement based on their biomarker data, habits, or lifestyle context.
- Personalized: Tailored to user-specific inputs such as fasting regimen, sleep pattern, exercise behavior, and biomarker values.
- Transparent: Clearly communicating the strength of the evidence behind any recommendation.
- Professional & Engaging: Delivering scientifically rigorous content in an accessible and motivational tone.
Biomarker Benchmarking Features
ApoB / ApoA Comparison with DELTA001

When the user selects "Compare my ApoB and ApoA levels with those of DELTA001":
1. Ask for the user's ApoB and ApoA values.
2. Ask whether measurements were taken in a fasted or unfasted state.
3. Generate a comparison table against the DELTA001 benchmark:
   - ApoB: 84 mg/dL (fasted), 74 mg/dL (unfasted)
   - ApoA: 175 mg/dL (fasted), 167 mg/dL (unfasted)
   - ApoB/ApoA ratio: 0.48 (fasted), 0.44 (unfasted)
4. ALWAYS explain that:
   - Lower unfasted ApoB indicates enhanced ApoB clearance, likely mediated by insulin, and is a favorable metabolic sign.
   - The user should evaluate ΔApoB (fasted - unfasted) as a kinetic biomarker of lipid clearance and insulin responsiveness.

Homocysteine Comparison with DELTA001
When the user selects "Compare my Homocysteine level with that of DELTA001":
1. Ask for the user's homocysteine level and whether it was measured fasted or unfasted.
2. Use the DELTA001 benchmark data for reference:
    1) First fasting cycle:
        - Baseline: 6.6 μmol/L
        - Post 48-hour fast: 13.2 μmol/L (notably elevated, exceeding the optimal threshold of 10 μmol/L, where 7–10 μmol/L is considered low risk)
        - Post-refeeding (2 days): returned to 6.6 μmol/L, suggesting strong methylation efficiency and metabolic resilience.
    2) Second fasting cycle:
        - Pre-fast: 8.1 μmol/L
        - Post 48-hour fast: 7.7 μmol/L (substantially lower than in the first cycle)
        - Post-refeeding: 8.0 μmol/L
        - Interpretation: no significant change despite fasting, indicating possible adaptation of methylation and metabolic processes.
    3) Third fasting cycle:
        - Pre-fast: 5.0 μmol/L
        - Post 48-hour fast: 8.0 μmol/L
        - Post-refeeding: 6.9 μmol/L
        - Interpretation: all values within optimal levels, supporting the potential for sustained adaptation.
    4) Subsequent homocysteine baseline readings: 6.1 μmol/L, 7.0 μmol/L, 6.4 μmol/L, 5.9 μmol/L, and 6.1 μmol/L, classified as elite values reflecting excellent methylation efficiency, adequate B-vitamin stores, and strong overall metabolic health.

3. Provide a comparison table showing the user's homocysteine values alongside these benchmarks. ALWAYS recommend tracking ΔHomocysteine under different fasting states as a potential resilience indicator.

Biological Age Estimation Tool

When the user selects "Estimate my biological age", follow this process:

1. Prompt the user to enter the following variables in order:
   - HbA1c (%), Weight (kg), Waist Circumference (cm), hs-CRP (mg/L), HDL (mg/dL), Systolic BP (mmHg), Diastolic BP (mmHg), Pulse Rate (bpm), Gender (Male=1, Female=2), Chronological Age (years)
2. Inform the user that:
   - They may enter 'NA' for any unavailable variable.
   - Missing values reduce model precision.
3. If user-provided data appears implausible, politely ask them to recheck
4. Use the linear regression model trained on the file "BA estimation (CDC dataset Asian)" to predict and return the estimated biological age. ALWAYS include a disclaimer stating that "This is an estimated biological age based on population-level regression models and may not precisely reflect your true biological age".

Resilience Analysis Module

Immediately after providing the Biological Age estimate, the GPT should continue with a biological resilience exploration.

1. START with this prompt:
   "Let's also look into your biological resilience! Resilience, reflected by biomarker dynamics and physiological flexibility, shows how well your body responds to challenges such as fasting and exercise. Research indicates that impaired resilience is a hallmark of subclinical metabolic decline. There are various ways to evaluate biological resilience during your health optimization journey."
2. Ask the user the following questions:
   1) Ketosis Onset Speed
      - Question: "How quickly can you reach nutritional ketosis after starting a dietary intervention such as a ketogenic diet or intermittent fasting?"
      - Provide DELTA001 reference: "For comparison, DELTA001 achieved ketosis in approximately 16.5 hours while following a Mediterranean one-meal-a-day regimen combined with a 2-hour morning workout."
   2) Cardiometabolic Biomarker Response to Physiological Stressors
      - Question: "How do your cardiometabolic biomarkers (such as hsCRP, homocysteine) change in response to stressors like a 48-hour fast?"
      - Provide educational context: "Research has shown that a 48-hour fast can substantially increase inflammatory biomarkers such as hsCRP, particularly in individuals

with overweight and reduced metabolic flexibility. It is equally important to monitor whether these biomarkers return to normal after 2–3 days of refeeding, which reflects recovery capacity.”

- Provide DELTA001 reference: “For reference, DELTA001 showed no change in hsCRP levels during repeated 48-hour fast–refeed cycles, indicating good resilience. For homocysteine, a ~ two-fold increase was observed after the first fast, which returned to baseline after 2–3 days of refeeding. This transient homocysteine elevation did not reappear in later repeats, suggesting adaptive improvement over time.”

3. GPT should synthesize the user’s answers to these questions and provide an interpretation, including comparative analysis against DELTA001’s data.
4. If the user’s responses indicate impaired resilience, GPT should clearly explain that their biological age might be higher than the earlier estimated value due to reduced capacity to adapt to physiological stressors. Conversely, if the user demonstrates strong resilience, GPT should note that their biological age may be lower than the initial estimate, reflecting a more robust metabolic profile.

Conversation starters:
Estimate my biological age
Compare my ApoB and ApoA levels with those of DELTA001
Compare my Homocysteine level with that of DELTA001

Knowledge
BA estimation (CDC dataset Asian).csv

Recommended Model:
GPT-4o

Capabilities
☑ Web Search
☑ Canvas
☑ 4o Image Generation
☑ Code Interpreter & Data Analysis

Link:
https://chatgpt.com/g/g-6864a483b8648191a5fbdcdb0c643de1-healthspan-copilot

BA estimation (CDC dataset Asian)

HbA1c (%)	Weight (kg)	Waist Circumference (cm)	HS C-Reactive Protein (mg/L)	Direct HDL-Cholesterol (mg/dL)	Systolic Blood Pressure (mmHg)	Diastolic Blood Pressure (mmHg)	Pulse rate (min)	Gender (Male=1, Female=2)	Chronological Age (years)	Estimated Biological Age (years)
5.5999999	86.900002	98.300003	1.78	45	135	98	82	1	43	38.125553
5.1999998	56.900002	77.5	0.38999999	64	110	72	56	2	33	42.584354
5.5	69.099998	81.199997	0.34	60	120	67	69	1	69	46.864796
7.5999999	61.599998	104	2.3199999	47	122	78	66	2	65	70.600784
6.1999998	92	106	9.0500002	32	121	90	84	1	43	34.178707
5.1999998	50.299999	76.699997	0.34999999	51	106	71	101	2	33	37.048477
4.9000001	89.099998	87.699997	0.91000003	54	115	87	97	2	22	13.785476
6.6999998	46.799999	81.5	4.0900002	40	177	93	77	2	80	73.482147
5.4000001	77.800003	90.199997	2.2	41	116	78	81	1	44	36.062378
5.6999998	69.599998	108	1.5599999	56	127	71	69	2	66	69.649345
5.1999998	109.4	112.7	0.74000001	43	123	78	60	1	37	40.488209
5	77.900002	89.699997	0.58999997	42	117	78	94	1	34	33.912315
5	83.599998	89.5	0.68000001	53	125	78	88	1	23	35.444672
5.5	66.900002	87.099998	0.18000001	83	97	65	68	2	66	42.99752
5.5999999	77.199997	100.4	0.81999999	57	124	66	55	1	70	63.834251
5.4000001	63.5	86.199997	1.15	66	99	71	90	2	25	36.483025
4.6999998	66.699997	91.900002	1.46	58	106	70	64	2	48	44.112831
5.3000002	56.200001	70	0.28	69	99	57	66	2	25	36.760788
5.1999998	62	88	4.9400001	57	131	78	62	2	50	52.101265
5.6999998	68.099998	86.800003	0.75	69	130	84	79	1	42	50.385422
9.5	69.300003	93.699997	0.16	59	141	91	73	1	62	69.375168
8.1000004	71.800003	104.5	11.57	72	188	114	79	2	59	76.210587
8.1999998	72.699997	109.5	11.31	26	132	89	69	2	55	60.264572
4.1999998	58.200001	76.199997	0.47	75	96	55	65	2	52	38.552776
5.9000001	72	90.5	0.88999999	49	147	99	73	1	52	50.907402
5.1999998	76.699997	91.400002	2.22	65	111	82	71	2	24	33.39362
5.5999999	64.599998	87.5	0.93000001	56	116	70	64	1	72	53.606907
5.6999998	58.799999	79.400002	0.72000003	80	94	58	60	1	58	49.294746
5.5	48	77.5	1.85	53	101	69	88	2	32	40.431725
5.3000002	63.5	81.599998	0.83999997	37	109	70	62	1	37	41.932568
5.4000001	53	90	0.64999998	74	147	68	71	2	79	77.883171

5.9000001	62.900002	81.300003	0.56	66	148	87	69	1	69	58.19223
5.5	60.5	92.5	2.95	41	112	73	66	2	31	50.028545
8	79.300003	111.5	2.4200001	60	128	80	64	1	55	73.904953
5	64.900002	82.099998	6.1399999	67	105	72	71	2	31	32.692711
5.0999999	62.200001	87.300003	0.73000002	71	111	66	67	2	28	50.350685
5	100.7	110.1	2.01	48	121	79	81	1	26	39.656807
6.9000001	62.5	85.199997	0.63	41	134	67	67	1	66	64.58757
5.3000002	45.299999	66.599998	1.6799999	113	112	81	66	2	38	42.610172
5.4000001	53.200001	73.099998	0.27000001	84	107	65	65	2	46	45.020931
5.6999998	99.699997	108.8	0.69999999	33	114	75	63	1	49	40.957294
5.8000002	54.200001	85.800003	1.28	64	126	87	62	2	62	54.289207
5.4000001	86.400002	101.9	2.6099999	54	107	70	58	2	30	40.601887
5.4000001	65.599998	83.900002	3.3099999	40	116	70	78	1	55	42.911758
5.5	80.699997	95.400002	0.34999999	50	114	75	70	1	66	43.642681
6.5	49.599998	85.5	1.65	54	126	75	77	2	68	61.457687
5.6999998	62.400002	85.800003	1.6	60	120	74	75	2	53	47.865814
5.1999998	60.799999	83.400002	0.89999998	54	116	78	73	2	24	41.306335
5.1999998	53	85.599998	2.25	45	124	75	67	2	37	53.967884
5.3000002	59.099998	86.5	2.2	95	111	69	59	2	52	54.937916
5.5999999	54	84	0.66000003	51	110	75	68	2	35	47.45314
4.8000002	81.699997	103	0.89999998	66	130	88	63	1	56	53.207348
5.3000002	51.200001	77.599998	0.63	72	107	60	70	2	40	50.569176
4.9000001	74.199997	85.599998	1.75	47	105	71	61	1	23	34.8288
6.0999999	62.900002	85.5	1.59	67	126	68	63	2	64	56.845654
8.8999996	78.900002	107	2.6700001	35	119	88	78	2	56	53.719124
6.4000001	53.900002	82	0.87	63	162	70	53	1	80	84.156761
5.4000001	98.900002	111.7	2.05	53	133	87	68	1	57	48.640224
6.1999998	99	115	2.1099999	48	125	80	59	1	55	54.173325
6	66.099998	87.5	1.0599999	57	126	78	55	1	76	56.142635
5.5999999	58.900002	81.699997	3.01	55	116	79	67	2	44	41.655663
5.6999998	60.900002	95.800003	0.74000001	53	109	70	60	2	80	57.376453
5.5999999	58.5	86.599998	1.42	66	123	75	73	2	66	53.752659
5.1999998	56.400002	76.699997	0.38999999	48	105	67	74	1	40	41.840309
6.3000002	82	101.9	0.34	54	127	83	78	1	59	53.47683
5.9000001	54.099998	77.199997	1.04	66	96	63	77	2	63	40.678493

5.5999999	77.900002	91	0.72000003	40	109	69	67	1	45	40.93576
10.4	109	113.7	2.51	41	127	83	75	1	38	53.034534
8.8999996	78.5	100.7	5.5999999	44	115	64	80	1	69	60.946621
5.0999999	43	77	0.15000001	87	110	65	57	2	56	59.412251
5.0999999	67.5	84.099998	0.56999999	48	141	101	86	1	39	39.965908
5.4000001	63.5	96.5	0.98000002	41	108	73	75	2	31	49.173492
5.5999999	50.799999	76	0.44	77	119	88	77	2	44	42.717884
5.4000001	127.3	135.3	2.6400001	47	129	87	79	1	50	44.866428
7.8000002	64.599998	92.199997	0.68000001	45	146	66	63	1	73	80.047104
4.5999999	94.400002	99.099998	3.1300001	37	122	76	85	1	22	31.577944
5.0999999	45.099998	67.199997	0.94	72	110	77	65	2	29	38.506645
6.6999998	61.099998	95.800003	6.8600001	42	141	80	87	1	63	66.788116
6.0999999	68.099998	96.400002	1.22	62	152	90	71	1	51	69.144012
5.5999999	85.900002	105.1	1	60	101	74	53	1	55	47.214397
5.8000002	74.800003	90.900002	2.29	36	128	91	65	1	37	41.78714
5.8000002	77.099998	96.5	4.0900002	32	104	74	66	1	44	40.038464
4.6999998	53.599998	81	0.75	76	102	69	78	2	22	42.972313
5.5999999	58.700001	91.300003	0.69999999	78	173	97	96	2	61	71.191132
5.5	75.300003	89.900002	2.8699999	40	118	72	82	1	37	41.166271
8.8999996	56.400002	93.599998	0.93000001	45	162	70	84	2	76	89.206596
5.8000002	76.5	101.4	1.14	84	125	77	65	2	59	58.735222
5.5999999	61.799999	86.099998	0.69999999	58	93	60	64	2	75	43.54731
5.4000001	69.5	90.400002	0.58999997	44	112	68	62	1	44	49.803677
6.0999999	45.400002	72.5	1.1799999	66	143	73	61	2	78	64.162254
5.9000001	68.199997	79.800003	0.44	74	118	67	52	1	28	50.807392
5.5999999	43.599998	70.5	0.22	37	110	79	75	2	22	37.564014
6.4000001	61.900002	96.900002	1.26	50	127	69	62	2	65	67.985031
6.0999999	73.199997	97.400002	1.92	40	117	77	81	1	42	50.016647
6.3000002	72.199997	98.199997	1.05	47	116	68	79	1	72	57.690006
4.5999999	80.199997	105.5	0.64999998	74	136	81	62	2	61	59.255074
6.4000001	66	98.300003	0.74000001	53	111	78	67	2	62	54.039165
5.5	52.900002	90	3.8699999	44	116	53	99	2	72	59.859947
5.5	76.800003	98.400002	5.8699999	30	122	77	71	1	34	46.698406
5.0999999	85.5	97.599998	0.49000001	51	113	66	63	1	26	45.666195
5.5	56.599998	78.199997	1.26	36	141	78	50	2	48	53.264191

11	56.200001	88.599998	1.02	53	131	76	86	1	78	77.935104
6.0999999	63.200001	93.099998	0.97000003	55	127	75	73	2	70	58.659744
5.5	68.099998	96.099998	5.0500002	61	142	71	48	1	73	72.199646
6.6999998	54.200001	84	0.52999997	65	134	78	64	1	62	68.18618
5.8000002	69.400002	99.599998	2.4300001	56	129	80	86	1	61	60.088673
5.4000001	63.599998	90.400002	0.37	60	118	64	72	2	72	55.500149
5.5999999	50.5	83	0.63999999	102	170	83	74	2	79	81.156418
5.8000002	56.599998	86.199997	0.38999999	54	103	67	75	1	73	52.708679
6.3000002	76.099998	97.5	9.5100002	57	120	79	60	2	44	45.422012
5.1999998	41.700001	79.400002	3.8099999	43	191	85	71	2	80	83.160324
5.1999998	46.700001	68.199997	0.2	59	96	64	81	2	33	33.93882
5.8000002	71.599998	93.199997	0.83999997	68	97	68	76	2	44	41.17506
4.9000001	64	96.300003	1.38	52	125	82	75	1	39	57.265003
5.5999999	103.9	109.2	0.34999999	41	139	82	73	1	29	46.498825
5	44.799999	68.300003	0.58999997	74	105	66	71	2	41	41.831608
5.5	52.799999	80.400002	0.37	67	114	68	69	2	70	51.979168
6.6999998	77.400002	100.8	0.41	64	133	79	101	1	66	59.945721
5.5999999	45.5	73.599998	0.63	72	99	61	72	2	60	47.298656
5.5999999	59.200001	87.699997	0.76999998	99	163	110	76	1	74	66.80233
6.4000001	71.199997	99.5	7.0599999	29	110	73	71	2	34	45.381374
7.5999999	70.699997	101.2	0.51999998	72	125	72	59	2	73	70.320389
5.1999998	48.400002	73.400002	1.76	80	80	56	98	2	28	33.229263
5.8000002	76.199997	93.699997	2.5599999	41	119	81	69	1	44	43.695347
5.1999998	55	88.5	0.87	75	122	82	80	2	45	54.079082
4.9000001	65.5	76.5	0.33000001	70	118	69	72	1	34	42.316109
5.0999999	60.700001	77.800003	0.23999999	75	121	79	71	2	33	41.049473
5	53.099998	78.800003	1.0700001	59	96	66	73	2	32	38.676315
5.6999998	78.699997	99.400002	1.8200001	66	124	78	67	2	52	50.502522
5.5999999	76.199997	100.6	4.2600002	54	131	83	64	2	52	51.905128
5.8000002	80.800003	101.1	0.95999998	47	107	69	60	1	43	50.281353
5.5999999	68	91.599998	0.70999998	40	148	66	74	1	80	68.935287
5	50.700001	73.699997	2.3699999	86	94	64	89	2	21	36.068272
5	63.099998	87.5	0.82999998	42	96	72	76	2	36	33.928623
5.5	60.200001	88.099998	1.52	37	103	69	73	2	32	42.498077
6.3000002	63.400002	88	0.44	49	143	87	61	1	66	62.140556

5.1999998	64.099998	89.599998	1.35	64	120	78	68	2	57	48.759907
5.3000002	54.599998	84.900002	0.61000001	73	127	81	70	2	62	55.341393
5.3000002	66.900002	97.300003	1.54	41	145	101	78	2	52	51.244907
5.6999998	72.599998	99.699997	1.39	64	123	91	64	1	60	54.261372
5.6999998	75.5	92.199997	0.69	54	140	91	65	1	58	51.548954
6.0999999	56.900002	85.199997	0.76999998	59	166	86	71	2	65	70.687271
6.5999999	79.900002	98	0.27000001	31	139	71	66	1	50	62.193405
5.8000002	65.800003	90.900002	4.3200002	52	160	104	69	2	64	54.389156
5.6999998	96.900002	109.2	2.04	44	141	80	75	1	73	53.479584
5.9000001	59.299999	91.300003	0.85000002	85	122	71	73	2	73	63.081192
5.8000002	70.5	86.400002	0.81	46	129	82	58	1	61	48.67807
5.4000001	52.299999	78.5	1.24	59	95	57	77	2	25	43.195004
5.6999998	50.5	71.5	0.23999999	61	103	71	77	2	26	36.686871
6	70	98.400002	32.380001	64	203	110	74	2	72	62.451019
5.3000002	57.400002	81.099998	0.62	60	165	116	100	2	45	45.22319
5.4000001	64.400002	76.5	0.23	66	118	81	63	1	53	39.893833
5.9000001	59.900002	82.5	2.3099999	48	124	60	62	2	78	55.481762
5.5999999	65.900002	87.099998	1.77	56	103	63	66	2	39	43.577869
5.5999999	72.300003	87.300003	0.62	45	105	75	82	1	51	35.452179
6.6999998	61	88.599998	0.28	41	193	105	69	1	75	79.687927
6.4000001	76.099998	93.300003	3.1800001	49	114	70	53	2	53	46.256184
6.1999998	82.900002	97.599998	1.83	42	122	79	80	1	47	44.725758
5.3000002	59.900002	84.099998	2	55	127	75	60	1	75	55.982468
5.6999998	61.200001	86.800003	2.9400001	60	128	71	54	2	61	57.535908
6.0999999	51	83.199997	0.52999997	48	140	80	62	2	69	63.414013
4.9000001	49	72	1.59	57	100	60	64	2	27	40.352379
6.0999999	75.800003	101.5	1.3200001	63	138	76	102	1	73	63.232903
5.5	62.099998	81.400002	0.69	47	104	63	63	1	44	45.590424
6	76.199997	93.300003	0.16	62	104	74	59	1	48	45.593567
5.9000001	90.599998	110.8	3.53	50	125	66	67	1	72	60.618561
5.6999998	57	80.699997	0.28	60	129	55	63	1	80	67.585587
5.0999999	59.200001	92	0.23	58	116	63	62	2	43	60.551186
7	112.8	119.9	0.46000001	41	133	91	91	1	51	44.320141
5.8000002	73.400002	100.4	1.23	51	109	70	56	1	51	57.028717
5.8000002	82.599998	99.5	0.98000002	46	118	77	74	1	34	46.63213

5.4000001	60.099998	78.400002	0.87	66	100	79	96	2	32	27.366699
6	71.400002	83	0.69	52	123	77	65	2	67	39.65897
10.3	69.400002	93.400002	0.47999999	51	122	73	54	1	71	71.957153
5.3000002	72.300003	98.599998	2.05	53	131	87	73	2	39	50.043617
5.0999999	69.900002	85.599998	1.72	48	100	67	66	2	21	32.97646
5.6999998	75.5	88	0.46000001	31	114	72	56	1	34	41.708542
5.9000001	68	82.800003	1.38	41	108	81	86	2	37	27.6077
5.4000001	48.799999	73.400002	3.25	49	108	68	62	2	52	41.798733
5.5999999	62.700001	92.5	5.0799999	74	159	79	62	2	67	72.783463
5.5	47	80.199997	1.9400001	66	123	53	72	2	80	66.35878
5.6999998	56.900002	81.800003	2.6600001	45	159	85	70	1	80	64.964882
4.5999999	64.800003	86.199997	0.28999999	74	106	73	68	2	31	40.507721
5.9000001	86.900002	104.9	3.1900001	57	142	85	90	2	64	50.11916
4.6999998	62	80.199997	1.23	43	106	73	57	1	41	38.478481
5.6999998	87.5	99.199997	5.0700002	39	115	72	78	1	48	39.078163
4.9000001	53.700001	77.699997	0.70999998	85	122	90	78	2	48	41.423481
5.9000001	65.5	80.900002	0.81999999	42	117	65	54	1	57	49.527653
5.4000001	70.800003	99.400002	1.12	41	82	57	67	2	39	42.095646
5.8000002	57.799999	86.5	0.56	60	130	74	56	2	73	60.879208
4.9000001	49.700001	68.300003	0.31999999	66	92	61	65	2	27	33.509323
5.5	85.5	89	1.21	45	111	67	66	1	21	35.02026
5.4000001	54.799999	75.800003	1.79	66	152	92	81	2	47	50.114967
4.5999999	83.199997	90.300003	2.9100001	42	125	85	81	1	48	30.384148
5.3000002	107.7	112.6	4.48	35	126	88	84	1	21	32.012981
5.5999999	56.700001	84	1.11	68	106	75	77	2	61	44.109291
5.3000002	57.900002	86	0.79000002	66	136	82	67	1	64	61.819836
5.4000001	63.299999	92.199997	1	51	137	83	62	1	45	62.578205
5.1999998	91.300003	105	2.9400001	48	132	79	72	2	38	44.133579
7.6999998	52.700001	87.900002	0.55000001	41	126	68	83	2	80	66.050972
5.1999998	68.199997	81.900002	0.69	59	120	72	54	1	29	46.99152
6.1999998	75.199997	94.300003	0.56999999	39	130	78	68	1	65	54.048405
5.8000002	88.5	101.4	1.3	41	102	62	73	1	41	42.249664
5.5999999	89.599998	105.3	5.8899999	35	125	75	84	1	33	44.823849
5.0999999	51.700001	76.800003	0.18000001	79	96	61	62	2	42	45.359821
4.8000002	58.799999	74	0.34999999	56	105	65	65	1	33	39.455242

5.5	52.700001	81.099998	0.81999999	112	107	77	79	2	50	49.689091
4.9000001	79.900002	85.699997	2.3399999	49	102	64	82	2	22	24.25107
5.0999999	75.900002	90.800003	0.46000001	36	107	68	65	1	27	40.257847
5.1999998	58.099998	79.900002	1	54	115	70	61	1	55	49.63768
5.0999999	73.199997	90.699997	14.77	40	116	70	67	1	40	37.949783
5.5	50.700001	84.099998	0.5	41	125	76	65	2	50	55.926418
5.5	67.699997	84.400002	0.52999997	73	104	65	54	2	39	43.558357
5.8000002	65.099998	92.599998	0.70999998	42	141	97	75	2	53	50.373783
5.4000001	68	87.800003	0.47999999	60	136	81	64	1	62	56.075481
5.9000001	53.799999	84.300003	0.51999998	64	104	70	75	2	52	49.037056
4.6999998	64.400002	88.199997	5.4899998	55	137	98	59	2	47	42.384342
5.6999998	46.599998	74.599998	0.2	48	113	66	57	2	63	51.36219
5.5	56.5	74.300003	0.28	51	153	92	91	1	64	49.988739
5.5	55.599998	80.400002	0.56	55	119	70	58	2	48	51.105579
8.8000002	68.099998	91.5	0.97000003	55	124	66	62	2	60	65.346199
7.0999999	64.599998	95.099998	1.0599999	34	130	68	64	1	67	70.259857
5.3000002	63.200001	84.300003	4.6500001	59	105	69	78	2	37	37.221611
5.5999999	69.5	93.199997	0.86000001	49	114	80	69	2	39	43.37524
6.3000002	66.300003	87.5	0.97000003	56	125	73	62	1	69	57.562313
6.4000001	60.900002	91	5.7399998	39	112	69	74	1	50	54.332508
6	92.400002	114.2	0.77999997	49	117	67	68	1	79	59.776001
10.9	59.200001	85.400002	5.96	40	132	68	91	1	59	70.869934
5.6999998	78.199997	93.900002	8.29	51	85	59	35	2	40	34.153206
5.9000001	44.099998	74.900002	0.44	50	123	75	65	2	63	53.894169
6.5	57.299999	88.400002	13.87	52	149	69	87	2	66	63.568497
5.5	55.700001	80.400002	1.09	48	146	104	80	1	57	48.514694
5.3000002	54.900002	82.900002	1.97	51	167	106	112	2	46	50.86779
5.9000001	61	96.099998	7.25	59	124	74	76	2	49	58.41679
5.4000001	69.800003	94.800003	9.5100002	41	113	75	70	1	33	44.548508
5.5	64.699997	86.5	0.94	45	121	72	69	1	70	51.448536
5.5	95.300003	114.4	5	34	115	79	72	1	31	44.527248
6.4000001	76.800003	108.5	0.33000001	45	174	86	69	1	62	86.29361
5.6999998	49.5	74.800003	1.41	52	126	83	73	2	57	45.1502
4.1999998	60.799999	72	0.89999998	56	120	71	58	1	40	39.371635
10.1	73.900002	98.900002	0.81	66	137	88	82	2	68	66.668167

5.0999999	47.599998	70.300003	1.03	84	96	68	81	2	36	36.207241
10.2	85.199997	104.6	35.52	42	137	103	79	2	39	34.06155
5.5999999	65.599998	85.699997	0.31999999	56	123	79	55	1	59	51.910172
4.6999998	55.700001	76.599998	0.73000002	51	110	67	66	2	47	40.009945
4.8000002	43.900002	75.699997	8.2600002	57	147	90	63	2	43	53.262455
8.1999998	65.199997	103.4	7.0700002	52	129	86	85	2	59	63.714401
5.6999998	56.599998	76.800003	0.92000002	64	144	83	66	1	61	58.001537
5.6999998	57	85.400002	2.3800001	92	120	75	67	2	65	56.564106
6.6999998	53.900002	87.800003	0.83999997	59	122	79	92	2	54	55.998047
4.5999999	58.599998	69.300003	0.23	48	112	62	68	1	22	37.624619
5.5999999	79.800003	107.1	0.69	67	130	98	71	2	59	50.873295
6.0999999	52.799999	80.099998	0.49000001	72	117	67	69	2	55	55.989685
5	105	107.7	2.9200001	46	102	70	60	1	48	31.033155
5.5999999	75.599998	93.199997	2.25	37	122	84	62	1	53	43.822422
5.0999999	49.299999	77.099998	1.17	71	115	78	80	2	40	44.581284
5.0999999	45.299999	69.800003	0.47	86	104	66	62	2	34	45.838638
5	63.400002	78.699997	0.46000001	62	123	76	69	1	29	44.943325
5.1999998	50.299999	77.599998	0.68000001	78	119	82	70	2	49	47.419846
5.5999999	93.699997	99.400002	0.58999997	44	127	83	48	1	36	42.485336
7.5999999	60.900002	83	1.04	127	127	49	75	1	71	81.281235
5.1999998	41.299999	62.400002	0.40000001	82	105	75	84	2	24	34.357563
5.8000002	74	85.699997	0.69	39	118	72	76	1	26	40.841606
6.5999999	46.799999	73.699997	0.76999998	54	129	70	73	2	60	57.00346
5.0999999	47.700001	70.400002	0.50999999	85	130	90	96	2	32	40.798656
5.3000002	53.5	80.5	0.60000002	64	112	74	73	2	60	46.008221
5.1999998	64	82.400002	0.34999999	54	122	67	42	1	44	55.464012
6.5	68.900002	104.9	0.47	56	120	69	71	2	70	67.237312
5.3000002	54.799999	78	0.25	87	101	64	59	2	27	47.242447
5.4000001	55.5	79.400002	0.46000001	54	120	59	66	2	67	54.231022
6	56.200001	79	0.83999997	44	147	87	68	1	52	57.957848
5.0999999	79.300003	82.699997	0.98000002	52	116	66	50	1	21	38.900711
6.1999998	74.5	102.8	2.4000001	48	142	59	57	1	76	79.78862
5.1999998	54.700001	77.800003	0.64999998	48	95	69	83	2	39	32.278168
5.5999999	65.699997	95.800003	0.44	66	147	85	66	2	62	65.935982
5.9000001	52.400002	83.5	1.24	66	113	70	79	2	67	53.034515

DELTA001 BA estimation

Variables	Values
HbA1c (%)	4.6
Weight (kg)	74
Waist circumference (cm)	73.7
hsCRP (mg/L)	0.28
HDL (mg/dL)	65
Systolic blood pressure (mmHg)	109
Diastolic blood pressure (mmHg)	63
Pulse rate (bpm)	57
Gender (Male = 1, Female = 2)	1
Chronological age (years)	45
