# Supplementary material for: DELTA: Strengthening human biological resilience with an N=1 digital health and dynamic biomarker protocol
Source: PLoS One. 2026 Aug 12;21(8):e0354234. doi: 10.1371/journal.pone.0354234 (PMC13465858; doi:10.1371/journal.pone.0354234)
Supplement: Supplementary file 5 [file pone.0354234.s007.pdf]

Review Report

Manuscript Number: PONE-D-25-65417

Title: DELTA: Fortifying Human Biological Resilience with an N=1 Digital Health and Dynamic Biomarker Protocol

Summary

This manuscript outlines a prospective, open-label N=1 case study, designated as the DELTA programme, which investigates a multimodal health optimisation protocol. The study focuses on a single subject, who undertook a regimen comprising Time-Restricted Eating (TRE), a Mediterranean diet, structured exercise, and sleep phase advancement. The authors introduce the concept of ‘dynamic biomarkers’, advocating for the measurement of recovery trajectories (resilience) following controlled stressors, such as a 48-hour fast, in lieu of relying solely on static physiological snapshots. The reported outcomes include improvements in sleep architecture and metabolic flexibility (specifically the glycolysis-ketosis switch), alongside the proposal of a ‘Resilience Index’ for biomarkers such as homocysteine. Additionally, a GPT-based ‘Healthspan Copilot’ is presented as a tool to support adherence.

General Assessment

The manuscript addresses a timely and pertinent subject within the burgeoning fields of digital health and geroscience: the paradigm shift from static ‘sick care’ metrics to the longitudinal, dynamic monitoring of ‘healthspan’. The conceptual framework of assessing biological resilience via controlled stressors is scientifically sound and methodologically intriguing.

However, the manuscript is constrained by significant limitations inherent to its design. Whilst the authors are transparent regarding the N=1 nature of the study and the fact that the subject is the Principal Investigator, the implications of this substantial bias have not been fully mitigated within the analysis. Furthermore, the manuscript currently reads as a concatenation of disparate pilot studies, spanning sleep, microbiome, biomarkers, and AI, rather than a cohesive validation of a unified platform. The section detailed the ‘GPT Healthspan Copilot’, in particular, lacks rigorous validation and appears disjointed from the wet-lab and wearable data. Consequently, I recommend Major Revisions to address these structural and methodological concerns before the manuscript can be considered for publication.

Specific Comments

1. Structure and Organisation

The manuscript attempts to cover a vast array of domains, including metabolism, sleep physiology, microbiome analysis, and artificial intelligence. Whilst ambitious, this breadth has resulted in a fragmented narrative. The transition from biological wet-lab data (e.g., homocysteine and ApoB dynamics) to the digital component is notably abrupt; the GPT section appears as an addendum rather than an integral component of the scientific enquiry. Furthermore, the inclusion of a qualitative interview section with the subject, who is also the author, is methodologically awkward. In a paper focused on objective biological resilience, analysing one's own 'participant experience' introduces a circularity that detracts from the empirical physiological data.

2. Literature Review

The review of the existing literature is generally adequate regarding healthspan and intermittent fasting but lacks necessary depth in two key areas. Firstly, the authors frame 'dynamic biomarkers' as a novel introduction; however, oral glucose tolerance tests (OGTT) and cardiac stress tests are established clinical paradigms of dynamic biomarkers. The literature review should better contextualise how the DELTA protocol's utilisation of fasting as a stressor compares to these existing challenge tests. Secondly, the paper would benefit significantly from a more robust comparison with seminal longitudinal multi-omics studies, such as the iPOP study by Michael Snyder. Differentiating DELTA from these existing deep-phenotyping cohorts is crucial to establishing its unique contribution to the field.

3. Methodology

The methodology presents the most contentious aspect of this work due to the 'participatory' design where the subject and the author are the same individual. Whilst described as participatory, it is, in essence, an unblinded self-experiment. The observed behavioural improvements, such as sleep consistency, are heavily confounded by the subject's intrinsic motivation to generate favourable data for publication. This creates a 'super-Hawthorne effect', making it impossible to disentangle the efficacy of the protocol from the motivation of the researcher.

Furthermore, the microbiome analysis contains a fundamental flaw. The study compares fasting states but admits to lacking a true pre-intervention baseline, as the subject had adopted the lifestyle years prior. The comparison with a single '3-Meals-A-Day' (3MAD) week is insufficient; one week is likely too brief to induce stable shifts in gut flora diversity, rendering the findings of 'non-significant' bacterial changes largely uninformative. Similarly, the 'retrospective' control period for sleep is methodologically weak. Sleep is highly seasonal and context-dependent;

comparing a retrospective period to a prospective intervention period without controlling for seasonality or workload variations weakens the causal claim that the DELTA protocol was the sole driver of improved sleep.

4. Model and Analysis

The authors propose a ‘Resilience Index’ for homocysteine calculated as 15 - AUC. This formula appears arbitrary. It is unclear why the baseline constant is set to 15, and whether this is derived from established clinical thresholds or theoretical principles. Without validation on a larger dataset or a derivation from first principles, this metric appears purely heuristic and lacks generalisability.

Regarding statistical analysis, the manuscript frequently employs t-tests and ANOVA on daily measurements (e.g., sleep stages, HRV). In time-series data, daily measurements are often autocorrelated, today’s value is dependent on yesterday’s. Standard ANOVA assumes independence of observations; thus, the effective sample size is likely lower than the number of days implies, and the reported p-values may be overstated. Finally, the validation of the GPT Copilot is anecdotal. There is no quantitative metric for the AI’s performance, such as accuracy against a ground truth or hallucination rates. As presented, it serves as a feature description rather than a rigorous scientific result.

5. Interpretation of Results

The emphasis on the absence of Fusobacteria as a positive outcome is speculative. Whilst this genus is associated with pathology, its absence in an N=1 study could simply be an individual idiosyncrasy rather than a result of the protocol. Additionally, whilst the quadratic fit for ketone recovery is an interesting observation, the claim of a ‘dose-dependent’ correlation is based on very few data points. Deducing a robust correlation or physiological ‘law’ from such sparse data is premature.

Recommendations

To meet the rigour required for publication, the authors are advised to address the following points:

- The language must be strictly curbed. Phrases suggesting this protocol ‘fortifies healthspan’ should be qualified to reflect that it fortified biomarkers in this specific subject, avoiding broader claims of efficacy.

- The authors must address the autocorrelation in the longitudinal data. Instead of simple t-tests between periods, time-series analysis (e.g., ARIMA or change-point analysis) should be considered to demonstrate that shifts in biomarkers were statistically significant deviations from the baseline trend, rather than random drift.
- A stronger justification for the mathematical formulation of the Resilience Index is required. If it cannot be theoretically justified, it should be presented merely as an 'Area Under the Curve (AUC)' comparison rather than a novel index.
- The discussion must explicitly address the limitations of the short 3MAD 'washout' period, acknowledging that seven days may be insufficient to reshape the microbiome.
- It is strongly recommended that the GPT section be moved to the Supplementary Materials or significantly shortened. It currently lacks the rigour of the physiological sections and distracts from the core biological findings.
- The 'Limitations' section must explicitly state that because the subject was the Principal Investigator, behavioural compliance cannot be generalised to a population lacking this specific motivation.
