# Supplementary material for: DELTA: Strengthening human biological resilience with an N=1 digital health and dynamic biomarker protocol
Source: PLoS One. 2026 Aug 12;21(8):e0354234. doi: 10.1371/journal.pone.0354234 (PMC13465858; doi:10.1371/journal.pone.0354234)
Supplement: Supplementary file 6 [file pone.0354234.s008.pdf]

Reviewer #1 Comments:

Summary

This manuscript outlines a prospective, open-label N=1 case study, designated as the DELTA programme, which investigates a multimodal health optimisation protocol. The study focuses on a single subject, who undertook a regimen comprising Time-Restricted Eating (TRE), a Mediterranean diet, structured exercise, and sleep phase advancement. The authors introduce the concept of 'dynamic biomarkers', advocating for the measurement of recovery trajectories (resilience) following controlled stressors, such as a 48-hour fast, in lieu of relying solely on static physiological snapshots. The reported outcomes include improvements in sleep architecture and metabolic flexibility (specifically the glycolysis-ketosis switch), alongside the proposal of a 'Resilience Index' for biomarkers such as homocysteine. Additionally, a GPT-based 'Healthspan Copilot' is presented as a tool to support adherence.

RESPONSE:

The authors thank the reviewer for supporting the findings reported in this manuscript. We have carefully addressed each of the comments and amended the manuscript for better clarity and transparency.

General Assessment

The manuscript addresses a timely and pertinent subject within the burgeoning fields of digital health and geroscience: the paradigm shift from static 'sick care' metrics to the longitudinal, dynamic monitoring of 'healthspan'. The conceptual framework of assessing biological resilience via controlled stressors is scientifically sound and methodologically intriguing.

However, the manuscript is constrained by significant limitations inherent to its design. Whilst the authors are transparent regarding the N=1 nature of the study and the fact that the subject is the Principal Investigator, the implications of this substantial bias have not been fully mitigated within the analysis. Furthermore, the manuscript currently reads as a concatenation of disparate pilot studies, spanning sleep, microbiome, biomarkers, and AI, rather than a cohesive validation of a unified platform. The section detailed the 'GPT Healthspan Copilot', in particular, lacks rigorous validation and appears disjointed from the wet-lab and wearable data. Consequently, I recommend Major Revisions to address these structural and methodological concerns before the manuscript can be considered for publication.

RESPONSE:

We would like to thank the reviewer for the helpful assessment. We acknowledge the limitations raised and have made point-by-point responses to all limitations mentioned. We have amended the manuscript to address each point raised. More importantly, we have also restructured the qualitative (participant experience) components of the study into a new section that connects to the wet-lab and wearable data sections. We believe the revised manuscript is now significantly improved in terms of overall clarity, transparency, structural flow, and methodological rigor.

Specific Comments

1. Structure and Organisation

The manuscript attempts to cover a vast array of domains, including metabolism, sleep physiology, microbiome analysis, and artificial intelligence. Whilst ambitious, this breadth has resulted in a fragmented narrative. The transition from biological wet-lab data (e.g., homocysteine and ApoB dynamics) to the digital component is notably abrupt; the GPT section appears as an addendum rather than an integral component of the scientific enquiry.

RESPONSE:

We thank the reviewer for pointing out the lack of overall flow and structure for the manuscript. We have amended the structure of the manuscript to organize all qualitative analyses including Healthspan Co-Pilot into a “Defining the Participant Experience” section that reconnects to the wet-lab biological data in the earlier sections of the manuscript.

Participant experience is often overlooked in technical research. However, it is an essential part of the innovation roadmap, and from a reader’s perspective, may be of particular interest, especially when human health research that may impact human behavior is involved. Additionally, while the core results in this paper are technical, an important element of this work was documenting the human element of the study. As such the participant experience section, now comprised of the qualitative assessment and GPT platform served this role. Regarding the Healthspan co-pilot, we have included an amended discussion pertaining to its use in dynamic feedback (not as a diagnostic), which is also an essential aspect of driving adherence.

MANUSCRIPT EDITS:

Lines 378 – 385: Defining the Participant Experience

In previous sections, we comprehensively analyzed biomarker trajectories, providing insight into dynamic physiological responses to lifestyle interventions. Beyond these quantitative measures, capturing the lived experience of the participant throughout the intervention period is essential for informing the scalability of N=1 participatory health frameworks. To complement these findings, we incorporated longitudinal qualitative assessments of participant experience at three specific timepoints over the course of the study, including reflections on behavioral adaptation and development of a proof-of-concept platform for driving behavioral adherence. Collectively, these components provide important insights into feasibility and adaptive strategies to support broader real-world implementation.

Lines 387 – 389: Defining the Participant Experience

Qualitative Assessment

Detailed and semi-structured interviews were conducted with DELTA001 at the study start (baseline), midpoint (after a 3MAD routine), and at the end of the study. Interview questions were not provided to DELTA001 in advance…

Lines 431 – 442: Defining the Participant Experience

Healthspan Copilot GPT

Given the inherent adherence gap in lifestyle habits within the general adult population, sustained engagement is essential to successfully improve healthspan. To address this, and

based on qualitative interviews, which highlighted that accessible and understandable data are crucial for participant adherence, we developed the "Healthspan Copilot." Designed specifically as a feedback mechanism rather than a clinical or diagnostic platform, this proof-of-concept companion system interprets user data and provides feedback to promote continued engagement and drive behavior change. Feedback is recognized as a driver of behavior change and its primary mechanism is described as a dynamic loop regulating behavior with a recurring comparison of current and target states. In the context of the "Healthspan Copilot", this is implemented as feedback on outcomes combined with self-monitoring. Metadata on goal monitoring shows a robust positive effect on goal attainment in a variety of contexts. Strong systematic evidence supports that feedback loops delivered eHealth and mHealth interventions improve lifestyle habits including diet, exercise, and sleep, making the concept particularly relevant for this study…

Lines 859 – 871: Study Limitations

The GPT-based Healthspan Copilot was retrospectively applied to DELTA001's data. As such, it has not yet been prospectively validated. Furthermore, because it serves strictly as a proof-of-concept adherence tool and not a diagnostic system, its performance was evaluated based on interaction consistency rather than quantitative AI metrics like hallucination rates or benchmark accuracy. In addition, the biomarker data used by the GPT for biological age estimation relied on conventional markers based on the available biomarker data in the population sample dataset. While the emerging set of biological resilience markers selected for the DELTA study (e.g., homocysteine, metabolic switch dynamics, etc.) were used to add additional support for potential further reductions in biological age, additional evidence will be needed to definitively support the use of these newer markers toward biological age estimation. Biological age is also not a universally applicable incentive to drive longitudinal biomarker collection or adherence toward healthspan-strengthening regimens. Additional incentives or approaches will likely be needed for future Copilot iterations, and future research should focus on testing the platform across larger, more diverse populations to fully evaluate its efficacy as a behavioral intervention.

Furthermore, the inclusion of a qualitative interview section with the subject, who is also the author, is methodologically awkward. In a paper focused on objective biological resilience, analysing one's own 'participant experience' introduces a circularity that detracts from the empirical physiological data.

RESPONSE:

Furthermore, we thank the reviewer for raising an important point on the inclusion of qualitative analyses as a part of the overall methodology. We acknowledge that conducting qualitative assessment on a participant who is also the principal investigator may introduce bias and should not be interpreted as qualitative evidence derived from this study. In addition to the necessary revisions, would like to emphasize that the questions were not made available to the principal investigator in advance, and the qualitative segment of the work was designed to assess behavioral and user experience insights, which still provide important insights that can inform future workflow design.

In response, we have revised the Defining Participant Experience (Results) and Study Limitations (Discussion) sections to clarify that these observations are presented as contextual, first-person insights on the DELTA framework rather than drawing evidence-based conclusions. The revisions have also emphasized that qualitative assessments may inform feasibility and strategies for broader implementation, without drawing generalized or evidence-based conclusions.

MANUSCRIPT EDITS:

Line 377 – 385: Defining the Participant Experience

In previous sections, we comprehensively analyzed biomarker trajectories, providing insight into dynamic physiological responses to lifestyle interventions. Beyond these quantitative measures, capturing the lived experience of the participant throughout the intervention period is essential for informing the scalability of N=1 participatory health frameworks. To complement these findings, we incorporated longitudinal qualitative assessments of participant experience at three specific timepoints over the course of the study, including reflections on behavioral adaptation and development of a proof-of-concept platform for driving behavioral adherence. Collectively, these components provide important insights into feasibility and adaptive strategies to support broader real-world implementation.

Line 794 – 798: Study Limitations

As the subject is also the principal investigator of the study, the participatory design also limits the generalizability of the findings to population level, that we also do not claim. The design made the intervention salient to DELTA001, therefore, unlike in this study, full adherence to the regimen cannot be expected in a large representative sample.

2. Literature Review

The review of the existing literature is generally adequate regarding healthspan and intermittent fasting but lacks necessary depth in two key areas. Firstly, the authors frame 'dynamic biomarkers' as a novel introduction; however, oral glucose tolerance tests (OGTT) and cardiac stress tests are established clinical paradigms of dynamic biomarkers. The literature review should better contextualise how the DELTA protocol's utilisation of fasting as a stressor compares to these existing challenge tests. Secondly, the paper would benefit significantly from a more robust comparison with seminal longitudinal multi-omics studies, such as the iPOP study by Michael Snyder. Differentiating DELTA from these existing deep-phenotyping cohorts is crucial to establishing its unique contribution to the field.

RESPONSE:

The authors thank the reviewer for this constructive feedback. We agree that our original phrasing overstated the novelty of 'dynamic biomarkers' as a general concept. We have revised the manuscript to acknowledge established clinical paradigms, such as the oral glucose tolerance test (OGTT) and cardiac stress tests. We have also added a new paragraph detailing the similarities and differences of the DELTA protocol with the established clinical paradigms of dynamic biomarkers. Of note, the existing challenge tests focus on only one functional pathway per test, while the DELTA protocol utilizes fasting and exercise as systemic stressors to assess metabolic resilience in multiple functional pathways including fuel switching (glucose and ketone), methylation efficiency (homocysteine), inflammation (hs-CRP), and lipid regulation (apolipoproteins).

Furthermore, we agree that a comparison with seminal longitudinal multi-omics cohorts is essential. We have included a discussion on the iPOP study (Reference below) in the Introduction section. We have clarified that while studies like iPOP captures longitudinal multi-omics trajectories and responses to naturalistic stressors (e.g., viral infections), the DELTA protocol provides a unique contribution by applying a standardized, time-bound physiological stressor (fasting) to actively probe metabolic resilience in a highly controlled manner.

Reference:

- Chen, Rui, et al. "Personal omics profiling reveals dynamic molecular and medical phenotypes." *Cell* 148.6 (2012): 1293-1307.

MANUSCRIPT EDITS:

Line 38 – 50: Introduction

Dynamic biomarkers, measurements taken in response to a physiological challenge, are a well-established clinical paradigm, exemplified by Oral Glucose Tolerance Tests (OGTT) and cardiac stress tests. However, these traditional tests typically probe a single functional pathway. The DELTA protocol utilizes fasting as a systemic stressor to assess metabolic resilience in multiple functional pathways. Unlike the OGTT, which measures the clearance of an exogenous substrate, fasting leads to a systemic shift in endogenous energy utilization. By evaluating multiple biomarker responses to this controlled perturbation, DELTA captures a broad spectrum of dynamic biomarker responses, providing a more comprehensive assessment of systemic resilience than traditional single-function tests. Additionally, the DELTA protocol offers a complementary but distinct approach to seminal longitudinal multi-omics cohort studies, such as the integrative personal omics profile (iPOP) by Chen et al. Studies like iPOP captures longitudinal molecular trajectories in response to unpredictable, naturalistic perturbations (e.g., viral infections). The DELTA protocol provides a unique contribution by applying standardized, time-bound physiological stressors (fasting/exercise) to actively probe metabolic resilience in a highly controlled manner.

3. Methodology

The methodology presents the most contentious aspect of this work due to the 'participatory' design where the subject and the author are the same individual. Whilst described as participatory, it is, in essence, an unblinded self-experiment. The observed behavioural improvements, such as sleep consistency, are heavily confounded by the subject's intrinsic motivation to generate favourable data for publication. This creates a 'super-Hawthorne effect', making it impossible to disentangle the efficacy of the protocol from the motivation of the researcher.

RESPONSE:

The authors thank the reviewer for raising an important point. We agree that the N=1 participatory design, especially when the principal investigator is also the study participant, may inherently introduce bias and may create a "Super-Hawthorne Effect" that intrinsically motivates the participant to adhere to the study for favorable data. We acknowledge that these factors may contribute to behavioral changes that drive the participant to adhere to this study. However, instead of interpreting the presented results as proof of generalizable efficacy, we designed this N=1 participatory framework to seek insights into behavioral-driven physiological responses to a suite of lifestyle interventions (e.g., fitness, sleep). These insights may then inform future larger-scale participatory, preventive health framework both on a population and individual levels.

Aside from the potential of "Super-Hawthorne Effect," this N=1 participatory design enables participant real-time awareness, access, and engagement with his own personal data (e.g., biomarker dynamics). This engagement, or "data-as-intervention" served as one of the components of motivations to adhere to the DELTA framework. Furthermore, qualitative analyses of the participant experience also revealed that continuous, real-time access and engagement – "data-as-intervention" – with his own data supported sustained adherence throughout the entire study. As such, motivational effects including data-as-intervention as

well as Super-Hawthorne Effect should not be treated solely as confounders; instead, collectively they form a part of the DELTA N=1 participatory health framework through which behavioral adherence and physiological responses may be influenced. More importantly, these motivational effects are an integral part of insights derived from this study, and they may potentially inform future design of N=1 participatory and population-scale studies.

Lastly, we would like to note that that even though motivational effects may have played a role in this study, we observed dynamic biomarker responses to DELTA interventions. Furthermore, the actionability of DELTA relies on long-term adherence to the regimens, which may be tailored to each individual, and motivational factors including engagement with own data and "Super-Hawthorne Effect" may help participants adhere to such regimens. In turns, regardless of the motivational factors, it is the long-term adherence of the participant that may result in improved health outcomes. While we cannot draw conclusions from these findings, they do provide insights that motivate further investigations,

In response to this comment, we have amended the manuscript to include potential motivational factors (e.g., Super-Hawthorne Effect) in the Study Limitations (Discussion) section.

MANUSCRIPT EDITS:

Line 787 – 791: Study Limitations

As discussed, data was visible to DELTA001 throughout the study. As highlighted in the qualitative interviews, it is highly probable that the study data directly influenced DELTA001 behavior. While we cannot discount the fact that the Hawthorne effect might have also contributed towards adherence, we establish in this study that long-term adherence may result in improved health outcomes, regardless of the underlying motivational factors.

Furthermore, the microbiome analysis contains a fundamental flaw. The study compares fasting states but admits to lacking a true pre-intervention baseline, as the subject had adopted the lifestyle years prior. The comparison with a single '3-Meals-A-Day' (3MAD) week is insufficient; one week is likely too brief to induce stable shifts in gut flora diversity, rendering the findings of 'non-significant' bacterial changes largely uninformative.

RESPONSE:

We thank the reviewer for noting that the absence of a true pre-intervention baseline is an important limitation in this study. As the participant had already been practicing this lifestyle for a prolonged period prior to their enrollment, it was not possible to obtain a microbiome profile before the initiation of the DELTA intervention. We do agree that this would constrain interpretations of long-term microbiome changes. However, the primary objective in our microbiome analysis in this study was to examine short-term changes associated with acute fasting (i.e., 48-hour fasts), rather than to assess the long-term effects of adopting such a lifestyle. To address this, we compared matched pre- and post-48 hour fasting states within multiple separate cycles, while allowing each pre-fasting sample to serve as an internal control for the corresponding post-fasting sample. Furthermore, the pre-48 hour fasting samples were used as a surrogate baseline for comparison with the 3MAD periods, as these samples most closely reflect the participant's habitual dietary intake and consumption behavior outside of the acute fasting episodes.

We would also like to clarify that the comparison between surrogate baseline and 3MAD states were not based on a single 3MAD week, but rather on multiple independent 3MAD weeks across separate cycles (Line 838). This improves robustness relative to a single-week comparison. While we acknowledge that one week may be insufficient to induce stable long-

term shifts in microbial diversity, the gut microbiome is known to be highly dynamic, with both compositional and functional changes reported to occur rapidly in response to dietary habit changes – ranging from within 24 hours to over several days (Reference: Medicine in Microecology, Nature) . Hence, while we do agree that the absence of significant bacterial changes should be interpreted cautiously, we believe that the repeated short-term comparisons would remain informative in assessing acute microbiome responses to fasting and dietary-state transitions in this participant.

MANUSCRIPT EDITS:

Line 839 – 844: Study Limitations

While we do not observe major changes in gut microbe composition between the PRE48 and POST48 conditions, which imply that the acute fasting regimen does not have apparent negative impacts on DELTA001 gut health, in the absence of a true baseline for the 3MAD intervention condition, it is difficult to conclude if a continuous 3MAD regimen would result in markedly different microbe diversity. It should also be noted that the 3MAD intervention phases were not intended to be a washout period, but a short-term dietary state intervention.

Similarly, the 'retrospective' control period for sleep is methodologically weak. Sleep is highly seasonal and context-dependent; comparing a retrospective period to a prospective intervention period without controlling for seasonality or workload variations weakens the causal claim that the DELTA protocol was the sole driver of improved sleep.

RESPONSE:

We thank the reviewer for highlighting this point and agree that the use of a retrospective control period for sleep introduces limitations. We did not intend to make a causal claim that the DELTA protocol was solely responsible for the observed improvements in sleep, but rather to describe an observed association during the intervention period. As such, we have revised the manuscript to emphasize that the observations were not causal and addressed the limitations that arise from using a retrospective control period in the study limitations.

MANUSCRIPT EDITS:

Line 342 – 346: Sleep Performance

These observations imply that the DELTA sleep intervention as part of the general DELTA protocol may have been effective at improving sleep performance, with statistical significance observed across certain sleep stages. However, it should be noted that contextual factors such as seasonality and workload were not controlled at the retrospective stage. Hence, we do not attribute the observed changes in sleep performance solely to the DELTA sleep intervention.

Line 806 – 813: Study Limitations

For example, sleep patterns can be influenced by a variety of factors (e.g., seasonality, workload, stress, shifts in daily routines), which may result in the differences between retrospective and intervention periods to not be entirely attributable to the DELTA protocol. Furthermore, as the sleep analysis compares mean sleep stage durations across three distinct periods rather than modeling nightly measurements as a continuous time series, nightly autocorrelation within each period is acknowledged as an inherent consideration. Future studies designed to examine continuous sleep dynamics would benefit from time-series appropriate methods such as ARIMA or change-point analysis. Additionally, while effort was made to keep meal composition consistent, variability was implicit.

4. Model and Analysis

The authors propose a 'Resilience Index' for homocysteine calculated as 15 - AUC. This formula appears arbitrary. It is unclear why the baseline constant is set to 15, and whether this is derived from established clinical thresholds or theoretical principles. Without validation on a larger dataset or a derivation from first principles, this metric appears purely heuristic and lacks generalisability.

RESPONSE:

We thank the reviewer for raising this important point and for the opportunity to clarify the rationale behind this metric. We have changed the terminology from "Resilience Index" to "Resilience Indicator" throughout the manuscript to better reflect its current role as a proof-of-concept measure rather than a definitive index. The mathematical formula of the resilience indicator is:

$$RI = D\cdot(15\ \mu mol/L) - AUC$$

Where:
D is the total number of days during a fast and the refeeding period.
15 µmol/L is the upper limit of a normal homocysteine level

The 15 µmol/L is not an arbitrary figure; it is the derived from established clinical thresholds specifically representing the clinical upper limit of normal for homocysteine levels (Reference: Cleveland Clinic). However, we agree with the reviewer that the justification and limitations of this parameter needed to be stated more explicitly. We have added sentences in the manuscript to explicitly state that the baseline of 15 is based on this established average upper limit. We have also noted that this threshold serves as an initial average that may be modified in future studies, and that subsequent research will be required to validate these parameters at a broader population scale.

MANUSCRIPT EDITS:

Line 229 – 235: Cardiometabolic and Pleiotropic Markers

Using the AUC values, a resilience indicator (RI), defined as a quantitative measure of an individual's ability to maintain durable homocysteine response following exposure to a stressor (TRE), was calculated using Equation (1). The baseline constant of 15 µmol/L is derived from the established clinical upper limit of normal for homocysteine levels. It should be noted that this threshold serves as an initial proof-of-concept average that may be modified in future studies, and subsequent research will be essential to validate these parameters at a broader population scale. The RI of the 3 cycles were calculated as 22.1 → 28.5 → 32.1 µmol·day/L (Fig. 3G).

Regarding statistical analysis, the manuscript frequently employs t-tests and ANOVA on daily measurements (e.g., sleep stages, HRV). In time-series data, daily measurements are often autocorrelated, today's value is dependent on yesterday's. Standard ANOVA assumes independence of observations; thus, the effective sample size is likely lower than the number of days implies, and the reported p-values may be overstated.

RESPONSE:

We thank the reviewer for the methodological comment which we have carefully considered. We would like to provide clarification on the design of the sleep analysis where this concern is directly relevant.

The analysis presented in Fig. 6F compares mean sleep stage durations across three temporally distinct and well-separated time-periods – a retrospective period (early 2024), study start (August 2024), and study end (April 2025) – rather than modelling nightly measurements as a continuous time series. Each period mean is derived from a discrete and bounded period, and the question analyzed is whether these period-level means differ, in which one-way ANOVA with Tukey's multiple comparisons is the conventional choice.

However, we do acknowledge that nightly sleep measurements within each period carries inherent autocorrelation, and that this is a meaningful consideration for interpretation of within-period variances. We have added to the Limitation section explicitly acknowledging this and noting that future studies designed to examine continuous dynamics would benefit from time-series appropriate methods. We also acknowledge that periods are not matched for potential confounding variables including seasonal variation and workload, which can affect the observed sleep improvements attributed to the DELTA protocol. Through the addition of these statements, we believe that the transparency of the manuscript has been strengthened, and we are grateful to the reviewer for prompting this clarification.

MANUSCRIPT EDITS:

Line 806 – 808: Study Limitations

For example, sleep patterns can be influenced by a variety of factors (e.g., seasonality, workload, stress, shifts in daily routines), which may result in the differences between retrospective and intervention periods to not be entirely attributable to the DELTA protocol.

Line 808 – 812: Study Limitations

Furthermore, as the sleep analysis compares mean sleep stage durations across three distinct periods rather than modelling nightly measurements as a continuous time series, nightly autocorrelation within each period is acknowledged as an inherent consideration. Future studies designed to examine continuous sleep dynamics would benefit from time-series appropriate methods such as ARIMA or change-point analysis.

Finally, the validation of the GPT Copilot is anecdotal. There is no quantitative metric for the AI's performance, such as accuracy against a ground truth or hallucination rates. As presented, it serves as a feature description rather than a rigorous scientific result.

RESPONSE:

The reviewer rightfully pointed out that the Healthspan Copilot is introduced without metrics such as accuracy against a verifiable benchmark and hallucination rate. We agree that such metrics are critical for platforms intended as diagnostic or clinical use. However, the Healthspan Copilot in this study serves as a proof-of-concept platform that provides feedback to promote adherence. Because adherence is foundational to strengthening healthspan, and continuous feedback is a key driver of that adherence (References Below), this tool operates within a substantially narrower scope than a clinical system requiring an AI validation study. Its role solely covers the following:

- Performing a parametric regression model for biological age estimation.
- Providing a structured biomarker comparison.
- Providing interpretative dynamic feedback where acute physiological changes trigger immediate feedback changes, a mechanism that is central to driving behavior change and fostering sustained adherence.

To make these points clear, we have revised the manuscript and articulated its role explicitly in both the Results and Study Limitations (Discussion) sections. The revision further explains why the tool is an integral part of the study: given the inherent adherence gap in lifestyle habits within the general adult population, deploying a feedback-driven system is essential to bridge that gap and successfully improve healthspan. Additionally, we have shortened the description of the results part to improve focus and conciseness. We believe these revisions improves the overall flow of the manuscript and provide a much clearer presentation of the Copilot's specific scope.

References:

- Bandura, A. (1991). Social cognitive theory of self-regulation. Organizational Behavior and Human Decision Processes, 50(2), 248–287.
- Carver, C. S., & Scheier, M. F. (1982). Control theory: A useful conceptual framework... Psychological Bulletin, 92(1), 111–135.
- Michie S, Richardson M, Johnston M, Abraham C, Francis J, Hardeman W, Eccles MP, Cane J, Wood CE. The behavior change technique taxonomy (v1) of 93 hierarchically clustered techniques: building an international consensus for the reporting of behavior change interventions. Ann Behav Med. 2013 Aug;46(1):81-95.
- Michie S, Abraham C, Whittington C, McAteer J, Gupta S. Effective techniques in healthy eating and physical activity interventions: a meta-regression. Health Psychol. 2009 Nov;28(6):690-701.
- Harkin B, Webb TL, Chang BP, Prestwich A, Conner M, Kellar I, Benn Y, Sheeran P. Does monitoring goal progress promote goal attainment? A meta-analysis of the experimental evidence. Psychol Bull. 2016 Feb;142(2):198-229.
- Singh, B., Ahmed, M., Staiano, A.E. et al. A systematic umbrella review and meta-meta-analysis of eHealth and mHealth interventions for improving lifestyle behaviours. npj Digit. Med. 7, 179 (2024).

MANUSCRIPT EDITS:

Lines 431 – 459: Healthspan Copilot GPT

Given the inherent adherence gap in lifestyle habits within the general adult population, sustained engagement is essential to successfully improve healthspan. To address this, and based on qualitative interviews, which highlighted that accessible and understandable data are crucial for participant adherence, we developed the "Healthspan Copilot." Designed specifically as a feedback mechanism rather than a clinical or diagnostic platform, this proof-of-concept companion system interprets user data and provides feedback to promote continued engagement and drive behavior change. Feedback is recognized as a driver of behavior change and its primary mechanism is described as a dynamic loop regulating behavior with a recurring comparison of current and target states. In the context of the "Healthspan Copilot", this is implemented as feedback on outcomes combined with self-monitoring. Metadata on goal monitoring shows a robust positive effect on goal attainment in a variety of contexts. Strong systematic evidence supports that feedback loops delivered eHealth and mHealth interventions improve lifestyle habits including diet, exercise, and sleep, making the concept particularly relevant for this study.

The "Healthspan Copilot", a custom GPT, was tested through user-chatbot interactions to verify the correct execution of its configured logic (Fig. 10A and Supplementary File 2). The GPT supports biomarker benchmarking against the subject (DELTA001), recommends tracking biomarkers changes across different fasting states (denoted as 'DELTA markers'), and estimates biological age alongside an analysis of biological resilience. It incorporates DELTA001's representative data and DELTA markers that are potential indicators of biological resilience, including ΔApoB, ΔHomocysteine, and Δhs-CRP. When prompted to "Compare my

ApoB and ApoA levels with those of DELTA001", the GPT requested the user's values, identified the fasting state, and generated a comparative analysis with DELTA001's benchmarks (Fig. 10B). For biological age estimation, the GPT consistently requested the 10 health metrics and returned an estimate based on a population sample-trained regression model (Fig. 10C and Supplementary File 2). Using his April 2025 metrics, DELTA001's estimated biological age was consistently 31.8 years, with potential further reductions due to his strong resilience profile. The result matched standard statistical software outputs and is substantially lower than his chronological age of 45. This aligned with favorable biomarker levels, including an HbA1c of 4.6%, weight of 74 kg, waist circumference of 73.7 cm, hs-CRP of 0.28 mg/L, HDL of 65 mg/dL, blood pressure of 109/63 mmHg, and a pulse rate of 57 bpm (Supplementary File 2). Although lacking a quantitative performance evaluation, these tests demonstrated the GPT reliably executed its configured logic and produced consistent, context-aware outputs.

Lines 859 – 871: Study Limitations

The GPT-based Healthspan Copilot was retrospectively applied to DELTA001's data. As such, it has not yet been prospectively validated. Furthermore, because it serves strictly as a proof-of-concept adherence tool and not a diagnostic system, its performance was evaluated based on interaction consistency rather than quantitative AI metrics like hallucination rates or benchmark accuracy. In addition, the biomarker data used by the GPT for biological age estimation relied on conventional markers based on the available biomarker data in the population sample dataset. While the emerging set of biological resilience markers selected for the DELTA study (e.g., homocysteine, metabolic switch dynamics, etc.) were used to add additional support for potential further reductions in biological age, additional evidence will be needed to definitively support the use of these newer markers toward biological age estimation. Biological age is also not a universally applicable incentive to drive longitudinal biomarker collection or adherence toward healthspan-strengthening regimens. Additional incentives or approaches will likely be needed for future Copilot iterations, and future research should focus on testing the platform across larger, more diverse populations to fully evaluate its efficacy as a behavioral intervention.

5. Interpretation of Results

The emphasis on the absence of Fusobacteria as a positive outcome is speculative. Whilst this genus is associated with pathology, its absence in an N=1 study could simply be an individual idiosyncrasy rather than a result of the protocol.

RESPONSE:

We thank the reviewer for noting that emphasizing the absence of *Fusobacteria* as a positive finding may be speculative in an N=1 study, and we agree that direct association cannot be established. As such, we had phrased in the manuscript that the absence of *Fusobacteria* could have potentially arisen from adhering to the protocol instead of making absolute claims that it was due to the protocol. However, we believe that this observation still remains noteworthy in the context of the participant's ethnic background and the longitudinal consistency of the findings. Previous population-level studies have shown that Fusobacterium is relatively more abundance in Southern Chinese and broader Chinese populations (Reference: Gut) compared with many Western cohorts, suggesting that its presence may be more common in individuals of similar ethnic and geographic backgrounds to our participant. Furthermore, recent data from Chinese populations in Singapore support the existence (Reference: medRxiv) of distinct gut microbiome signatures of Fusobacterium presence in this demographic. Hence, the persistent absence of detectable *Fusobacteria* in our participant would be of interest rather than dismissed as idiosyncratic. Importantly, the results were not based on a single sample but across 20 microbiome assessments collected over 6 months.

Fusobacteria was consistently undetected, which strengthens the observation despite the limitations of a single-subject design.

MANUSCRIPT EDITS:

Line 849 – 852: Study Limitations

While we cannot draw a definitive causal relationship between adherence to the DELTA regimen and the consistency of Fusobacteria absence in the participant's microbiome, the persistent and longitudinally validated absence of Fusobacteria serves as a positive indicator of the tolerance of the DELTA regimen.

Additionally, whilst the quadratic fit for ketone recovery is an interesting observation, the claim of a 'dose-dependent' correlation is based on very few data points. Deducing a robust correlation or physiological 'law' from such sparse data is premature.

RESPONSE:

We thank the reviewer for this helpful comment, and we acknowledge that establishing a robust correlation from this proof-of-concept study is premature. In response, we have added "potential" to all mentions of "dose-dependent" correlation in the manuscript to indicate the preliminary correlation observed in this study. In addition, we have expanded the Study Limitations (Discussion) section to emphasize that further validation in larger cohort studies is required to confirm and generalize the potential dose-dependent correlation observed in this study.

MANUSCRIPT EDITS:

Line 142 – 145: Introduction

Cardiometabolic flexibility, sleep, microbiome dynamics, and performance outcomes were assessed, with evidence of potential dose-dependent biomarker responses, exceptional cardiometabolic and pleiotropic biomarker resilience, marked sleep performance improvement, and platforms for longitudinal monitoring adherence.

Line 165 – 167: Metabolic Resilience and Flexibility

Of note, a potential dose-dependent correlation of glycolytic onset rate, ketotic recovery rate, and total substrate switch rate were observed (Fig. 2C).

Line 817 – 819: Study Limitations

More work is needed to determine their population-scale impact, if any, toward driving improved human longevity outcomes. For example, the observed dose-dependent effects in metabolic switching rates should be further validated in larger cohort studies.

Recommendations

To meet the rigour required for publication, the authors are advised to address the following points:

- The language must be strictly curbed. Phrases suggesting this protocol 'fortifies healthspan' should be qualified to reflect that it fortified biomarkers in this specific subject, avoiding broader claims of efficacy.

RESPONSE:

The authors thank the reviewer for this important comment. We have revised the language throughout the manuscript to avoid overgeneralization. Specifically, we replaced statements (including the manuscript title) suggesting "healthspan fortification" with "strengthen" to clarify that DELTA interventions were associated with improvements in selected biomarkers, avoiding broader claims of efficacy. All "fortifies" have been replaced with "strengthens."

MANUSCRIPT EDITS:

Manuscript Title:

DELTA: Strengthening Human Biological Resilience with an N=1 Digital Health and Dynamic Biomarker Protocol

- The authors must address the autocorrelation in the longitudinal data. Instead of simple t-tests between periods, time-series analysis (e.g., ARIMA or change-point analysis) should be considered to demonstrate that shifts in biomarkers were statistically significant deviations from the baseline trend, rather than random drift.

RESPONSE:

We thank the reviewer for this recommendation and have considered its application to the manuscript. We would like to clarify the intent of the sleep analysis in Fig. 6F, as this relates directly to the appropriateness of the methods used.

The sleep analysis was designed to address specific, bounded time periods – if the mean sleep stage durations differed across these distinct time periods. It was not designed to model continuous sleep trajectories, nor to make claims about the rate or shape of change across the study period. Given this intent, one-way ANOVA with Tukey's multiple comparisons is the conventional and appropriate method. Applying ARIMA or change-point analysis would reframe the question towards one of continuous trend modeling and forecasting, generating claims regarding drift and rate of change that go beyond what this analysis was designed to claim.

We have incorporated this recommendation by adding to the Limitation section noting that future studies designed to examine continuous dynamics would benefit from time-series methods such as ARIMA or change-point analysis. We are grateful to the reviewer for strengthening the manuscript's discussion on longitudinal analyses.

MANUSCRIPT EDITS:

Line 808 – 812: Study Limitations

Furthermore, as the sleep analysis compares mean sleep stage durations across three distinct periods rather than modeling nightly measurements as a continuous time series, nightly autocorrelation within each period is acknowledged as an inherent consideration. Future studies designed to examine continuous sleep dynamics would benefit from time-series appropriate methods such as ARIMA or change-point analysis.

- A stronger justification for the mathematical formulation of the Resilience Index is required. If it cannot be theoretically justified, it should be presented merely as an 'Area Under the Curve (AUC)' comparison rather than a novel index.

RESPONSE:

The authors thank the reviewer for this helpful recommendation. We acknowledge that the resilience index is not yet theoretically validated. We have clarified its current role as a proof-of-concept measure rather than a definitive index in the response to Comment #4: Model and Analysis (Page 8). We have also made necessary changes in the manuscript to reflect this point and noted the need to validate this measure in further studies.

- The discussion must explicitly address the limitations of the short 3MAD 'washout' period, acknowledging that seven days may be insufficient to reshape the microbiome.

RESPONSE:

We thank the reviewer for noting that a 7-day 'washout' period would likely be insufficient to fully reshape the gut microbiome if the 3MAD phase were intended as a true "washout" period. However, in this study, the 3MAD phase was not intended to serve as a washout. The participant's habitual dietary behavior is one-meal-a-day (OMAD), which is congruent to that of the pre-48 hour fasting state, and these samples were therefore considered the most suitable surrogate baseline for the 3MAD intervention phase. To clarify, the 3MAD phase was intended to be a short-term dietary state comparison rather than an attempt to washout the effects of the 48-hour fasting cycles. To avoid this misunderstanding, we have revised the manuscript to clarify that the 3MAD period is not designed to be and should not be interpreted as a washout period.

MANUSCRIPT EDITS:

Line 839 – 844: Study Limitations

While we do not observe major changes in gut microbe composition between the PRE48 and POST48 conditions, which imply that the acute fasting regimen does not have apparent negative impacts on DELTA001 gut health, in the absence of a true baseline for the 3MAD intervention condition, it is difficult to conclude if a continuous 3MAD regimen would result in markedly different microbe diversity. It should also be noted that the 3MAD intervention phases were not intended to be a washout period, but a short-term dietary state intervention.

- It is strongly recommended that the GPT section be moved to the Supplementary Materials or significantly shortened. It currently lacks the rigour of the physiological sections and distracts from the core biological findings.

RESPONSE:

We thank the reviewer for this helpful recommendation. We acknowledge the limitations of the Healthspan Copilot GPT and have clarified its role as a proof-of-concept platform that provide feedback for the purpose of promoting behavior change as elaborated in the response to Comment #4: Model and Analysis (Page 8). We have made necessary changes in the manuscript to improve the overall connection to the physiological sections and elaborated on the rationale of having such a platform in a study that advocates participatory health and adherence to lifestyle changes. We have also shortened the results part to improve focus and conciseness. We believe the Healthspan Copilot GPT section is now an integral and relevant part of the study after the amendments.

- The 'Limitations' section must explicitly state that because the subject was the Principal Investigator, behavioural compliance cannot be generalised to a population lacking this specific motivation.

RESPONSE:

We thank the reviewer for suggesting important discussion points that improve transparency of the study. We agree that a higher-level generalization of behavioral compliance may not be appropriate with this N=1 study design, particularly when the Principal Investigator served as the study participant. To emphasize this limitation, we have included a statement in the “Study Limitations” section highlighting this important discussion point.

MANUSCRIPT EDITS:

Line 794 – 798: Study Limitations

As the subject is also the principal investigator of the study, the participatory design also limits the generalizability of the findings to population level, that we also do not claim. The design made the intervention salient to DELTA001, therefore, unlike in this study, full adherence to the regimen cannot be expected in a large representative sample.

Reviewer #2 Comments:

This is a highly innovative and fascinating study. Using an N=1 digital health protocol to measure biological resilience and healthspan is a very forward-thinking approach to precision medicine.

I appreciate the methodological rigor and transparency you applied, especially given that the subject is one of the authors. The integration of AI, wearables, and lifestyle interventions (like time-restricted eating and strength training) provides a fantastic proof-of-concept for how we can track dynamic biomarkers in real-time. This will serve as an excellent foundation for larger population-level trials.

I have no changes to request. Congratulations on a unique and well-executed paper.

RESPONSE:

The authors thank the reviewer for supporting the findings reported in this manuscript. We have additionally amended the manuscript for better clarity and transparency.
