# Supplementary material for: DELTA: Strengthening human biological resilience with an N=1 digital health and dynamic biomarker protocol
Source: PLoS One. 2026 Aug 12;21(8):e0354234. doi: 10.1371/journal.pone.0354234 (PMC13465858; doi:10.1371/journal.pone.0354234)
Supplement: Supplementary file 7 [file pone.0354234.s010.pdf]

Academic Editor Comments:

While the majority of the reviewer and editorial concerns have been responded to, I would like to note a few remaining points from the latest round of review for your attention and action below

This is a interesting but quite unusal study where the investigator, the subject, and the patent holder are the same person, the interpretive choices made throughout the manuscript, about which findings to emphasize, how to frame trajectories, what language to use around causation, cannot be fully disentangled from personal investment. While the authors acknowledge this, one would wonder for this means for the conclusions generated

RESPONSE:

The authors thank the editor for this observation. We acknowledge that the overlap of investigator, participant, and patent holder naturally invites scrutiny of interpretive objectivity. However, this convergence is intrinsic to the N=1 participatory framework, a design not intended to establish generalizable efficacy, but to yield mechanistic and behavioral insights into within-individual responses to lifestyle interventions. It is precisely this overlap that enabled the longitudinal depth and sustained adherence that characterized this work.

We would also like to emphasize that the investigator serving as the study participant is an approach with established precedent, and one attracting growing interest within the quantified self, participatory health, and related communities. Importantly, however, the present work extends beyond passive self-tracking or observational self-measurement. This is an interventional study involving pre-defined physiological stressors, serial biomarker measurements, and longitudinal assessment of responses over time. These features enabled iterative hypothesis generation and testing within a real-world setting, while allowing dynamic physiological changes to be examined at the N=1 level with a granularity that is difficult to achieve with conventional group-level designs.

Furthermore, this study design also enables real-time, continuous engagement with one's own biomarker data, which served as a meaningful driver of sustained adherence to the DELTA framework, which we term "data-as-intervention." Our qualitative analyses also corroborated that this mode of engagement would be difficult to achieve in a conventional externally administered trial. We have made amendments in the manuscript to frame our conclusions accordingly as hypothesis-generating findings to inform future larger-scale participatory, preventive health frameworks at both the population and individual levels.

MANUSCRIPT EDITS:

Lines 791 – 800 (Study Limitations):

There are a number of limitations to this study. Firstly, this study was conducted on a single subject. Therefore, population-level conclusions should not be derived from this work. We would also like to emphasize that the investigator serving as the study participant is an approach with established precedent, and one attracting growing interest within the quantified self, participatory health, and related communities. Importantly, however, the present work extends beyond passive self-tracking or observational self-measurement. This is an interventional study involving pre-defined physiological stressors, serial biomarker measurements, and longitudinal assessment of responses over time. These features enabled iterative hypothesis generation and testing within a real-world setting, while allowing dynamic physiological changes to be examined at the N=1 level with a granularity that is difficult to achieve with conventional group-level designs.

Lines 921 – 924 (Conclusions):

In sum, the DELTA trial has illuminated the potential of leveraging participatory approaches to drive and sustain behaviors that can strengthen the healthspan, generating hypothesis-driven insights that may inform the design of future larger-scale participatory, preventive health frameworks at both the population and individual levels.

Closely related to this is the GPT Copilot evaluation, which as I noted is essentially the authors confirming that a tool they built and hold a patent on works as they designed it to. That is not validation in any meaningful scientific sense and the paper should say so more clearly. There is no external validation cohort, no benchmark comparison, and no pre-registered performance criteria.

RESPONSE:

We thank the editor for this insightful comment. We agree that the Healthspan Copilot GPT evaluation does not constitute scientific validation, and we acknowledge the absence of external validation cohort, benchmark comparison and pre-registered performance criteria. We would like to emphasize that the Healthspan Copilot GPT work was presented strictly as a proof-of-concept feedback mechanism for promoting engagement and driving behavior change, a consideration that is critical to the broader goal of health optimization. Its evaluation in the study therefore reflects feasibility assessment only, and rigorous validation remains an important direction for future work. We have amended the manuscript to make this point more clearly.

MANUSCRIPT EDITS:

Lines 437 – 441 (Healthspan Copilot GPT):

Presented strictly as a proof-of-concept feedback mechanism, without external validation cohorts or pre-specified performance criteria, this companion system interprets user data and provides feedback to promote continued engagement and drive behavior change. Its evaluation here therefore reflects feasibility assessment only, and rigorous validation remains an important direction for future work.

The biological age result is the kind of finding that will attract popular attention and it rests on a relatively thin methodological foundation. A regression model trained on a general population sample and applied to an individual who sits at the extreme healthy end of nearly every input variable is going to produce a dramatic estimate almost by construction. The authors should put this into consideration when making inferences.

RESPONSE:

We agree with the editor's point regarding the interpretive scope of population-trained regression models when applied at the individual level. We would note, however, that the direction of the result, a healthier biomarker profile yielding a lower biological age estimate, reflects the intended mechanism of these methods rather than a limitation in their application. While biological age estimates can vary across methods (e.g., DNA methylation clocks, telomere length, statistical-based method), this directional relationship between favorable biomarkers and younger estimated age is a consistent feature across approaches. There is currently no gold standard for biological age estimation, and the OLS regression-based approach used in our work is among the established methods in the field [1,2].

Within the DELTA framework, biological age was leveraged as a gamification strategy to support adherence and behavior change rather than a definitive clinical measure. For those

already maintaining healthy habits and having favorable health, tracking biological age provides reinforcement to continuously sustain those behaviors. In contrast, for those whose biological age exceeds their chronological age, the metric may serve as a tangible, accessible prompt to reflect on current habits and consider lifestyle changes. This gamification strategy is what makes biological age a meaningful tool within a participatory health framework. We have made amendments in the manuscript to ensure this finding is framed within the hypothesis-generating scope of this N=1 design, and not as a standalone efficacy claim.

References:
1. Bafei, S.E.C., Shen, C. Biomarkers selection and mathematical modeling in biological age estimation. npj Aging 9, 13 (2023).
2. Skinner, C.M., Conboy, M.J. & Conboy, I.M. DNA methylation clocks struggle to distinguish inflammaging from healthy aging, but feature rectification improves coherence and enhances detection of inflammaging. GeroScience 47, 3043–3060 (2025).

MANUSCRIPT EDITS:

Lines 462 – 465 (Healthspan Copilot GPT):

This estimate, computed from an OLS regression model derived from a general population sample, should be interpreted within the hypothesis-generating scope of this N=1 design rather than as a definitive measure of biological rejuvenation.

The sleep findings are genuinely interesting but the control condition is weak. The retrospective data comes from before the study began, under different monitoring conditions, and without any of the other protocol elements in place. You cannot isolate the sleep timing intervention from everything else that changed simultaneously.

RESPONSE:

We thank the editor for this observation. We agree that the use of a retrospective control has inherent limitations, and we would like to clarify that we are not intending to make causal claims attributing sleep improvements to any of the interventions introduced by the DELTA protocol in isolation.

We acknowledge that the DELTA protocol is combinatorial by design and have been explicit that the observed sleep improvements reflect an association during the intervention period rather than a causal relationship with any specific element of the protocol.

In response to this concern about the inability to isolate the sleep timing intervention from other changes from the protocol, we have emphasized this limitation in the manuscript, specifically within “Sleep Performance” in the Discussion section. We believe that these changes more prominently position the sleep findings as observations rather than as evidence of a specific effect from an isolated intervention. We have additionally amended the key takeaway for Sleep Performance in Figure 1 to reflect this change.

MANUSCRIPT EDITS:

Lines 570 – 574 (Discussion – Sleep Performance):

We estimate that moving sleep time earlier, alongside other elements of the DELTA sleep regimen including improved sleep hygiene, morning fitness, TRE regimen, and possible gamification driven by wearables use, may have collectively contributed to the improvements

in DELTA001's sleep performance. Given this the relative contribution of each element of the protocol cannot be determined from this study design.

Lines 585 – 587 (Discussion – Sleep Performance):

In this study, we estimate that the combined elements of the DELTA sleep regimen, including moving sleep time earlier, may have helped DELTA001 achieve consistent sleep patterns that is known to be protective against metabolic disease, cognitive decline, and improve cardiovascular health.

FIGURE 1 – Key Takeaways (Sleep Performance):

"Earlier sleep timing, as a part of a combined protocol, may lead to improved sleep performance."

Reviewer #3 Comments:

This paper highlights an exciting case report study that evaluates a health optimization protocol through fasting, physical activity, dietary changes, wearables, and biomarker measurements. The idea of exploring biological resilience via changes in dynamic biomarkers is a fresh one and might prove very useful in precision health. Nevertheless, several important issues need to be addressed before the publication of this research.

RESPONSE:

We would like to thank the reviewer for the helpful assessment. We acknowledge the issues raised and have amended the manuscript to address each point raised. We believe the revised manuscript is now significantly improved.

First of all, there is a problem with the overinterpretation of results. The manuscript often makes conclusions on metabolic resilience, adaption, and priming in relation to changes in biomarkers. This is not entirely correct because this is a N=1 longitudinal design with no control groups used. Therefore, it would be better to consider this information just as exploratory.

RESPONSE:

We thank the reviewer for this comment and would like to take the opportunity to clarify the methodological framework of the study, as we recognize that N=1 trials are distinct in their design.

In this N=1 trial, the participant acts as their own control across systematically administered interventions, rather than requiring a separate untreated control group. The structure of within-subject repeated measures makes up the control architecture of the design. The comparisons made in this trial are reflective of this design logic. This is a recognized approach to within-subject inference, supported by reporting guidelines in the form of the CONSORT extension for reporting N-of-1 trials (CENT) and the SPIRIT extension for N-of-1 trials (SPENT) – in our trial, SPENT was used to inform study design, CENT guidelines were used to guide the writing of the manuscript, and both checklists can be found in S1 File.

MANUSCRIPT EDITS:

Lines 129 – 131 (Introduction):

This structure of repeated within-subject measures, in which DELTA001 served as their own control across systematically administered conditions, constitutes the control architecture of the study.

Lines 478 – 481 (Discussion – DELTA Protocol):

Therefore, in the absence of a separate untreated control, with the exception of the sleep performance assessment, no claims are made regarding the efficacy of the DELTA protocol in mediating substantial improvements to the health and performance profile of DELTA001, beyond what is supported by the within-subject comparisons described throughout this study.

Another significant issue that needs to be discussed is the combination of several interventions: time-restricted eating, fasting, resistance and high-intensity interval training, diet adjustment, and supplementation. The fact is that this study did not evaluate any particular intervention separately. Hence, it becomes impossible to find out what interventions have led to metabolic, athletic performance, or biomarker changes.

RESPONSE:

We thank the reviewer for this important comment. We agree that when multiple interventions from the DELTA protocol are implemented concurrently, the individual contribution of each intervention to the participant's physiological response (e.g., biomarker profile) may not be determined. Furthermore, we would like to clarify that this study was designed to assess DELTA001's longitudinal health trajectories following these habitual lifestyle changes altogether, instead of evaluating the effects of individual interventions. Nevertheless, we agree that evaluating the contribution of each intervention separately may provide important insights into their respective effects on human biology/physiology and health. Future larger-scale N=1 and population studies designed to assess individual lifestyle interventions (e.g., fasting) would be important. In response to this comment, we have acknowledged this limitation and revised "Study Limitations" in the Discussion section.

MANUSCRIPT EDITS:

Lines 829 – 833 (Discussion – Study Limitations):

In the DELTA protocol, multiple interventions were implemented concurrently and therefore, the individual contribution of each intervention to DELTA001's physiological responses could not be determined. Future larger-scale N=1 and population-based studies designed to evaluate individual lifestyle interventions, such as TRE, may provide valuable insights into the effectiveness of specific lifestyle modifications.

The suggested Resilience Indicator as well as biomarkers are very promising concepts. However, there is no data on reproducibility, reliability, prediction, and clinical validity of the measures, and they have to be treated as initial ones that require confirmation in larger and more diversified samples.

RESPONSE:

We thank the reviewer for raising this important point. We acknowledge that the resilience indicator is not yet validated in terms of reproducibility, reliability, predictive value, and clinical relevance. We have amended the manuscript to clarify its current role as a proof-of-concept measure rather than a definitive biomarker, and noted the need to further validate this measure in future studies.

MANUSCRIPT EDITS:

Lines 234 – 237 (Results – Cardiometabolic and Pleiotropic Markers):

The RI is presented here as a proof-of-concept measure rather than a validated biomarker. Its reproducibility, reliability, predictive value, and clinical validity have not yet been established, and the threshold of 15 µmol/L should be treated as an initial construct that may be refined as confirmation in larger, more diversified samples is sought in future studies.

Furthermore, it would also be useful to improve statistical analysis since several conclusions seem to be driven mostly by visual data analysis even in the absence of significant effects. It is necessary to distinguish more clearly between results of data analysis and hypotheses.

RESPONSE:

We thank the reviewer for this comment. We wish to clarify that formal statistical comparisons were applied where the study design supported a question's analysis (e.g., biomarker comparisons (PRE48 vs. POST48), microbiome diversity between feeding conditions, or sleep

duration across study periods). For other outcomes, visual presentation was a deliberate choice, as statistical tests would go beyond what the study was designed to support and would generate claims that we do not intend to make.

In addition, another possible threat to validity is that the participant who took part in the research is an author. While it is noted in limitations, it should be discussed more thoroughly.

RESPONSE:

We appreciate the reviewer pointing this out. We have addressed this concern in detail within our response to the Academic Editor's first comment (see pages 1–2).

Some minor points include specifying laboratory procedures of sample standardization, providing information about intra-individual variation in the study participants, and including effect sizes and confidence intervals in analysis.

RESPONSE:
We thank the reviewer for raising this important point. We would like to clarify that all clinical laboratory procedures performed in this study, including homocysteine and hsCRP measurements, were conducted by a clinical-grade laboratory certified by the Singapore Ministry of Health. Microbiome analyses were performed by AMILI Pte. Ltd., a precision gut microbiome company with ISO 9001 and ISO 27001 accreditation and established collaborations with government research initiatives in Singapore. These procedures were therefore conducted in accordance with recognized laboratory quality standards, and the results are therefore considered to be of clinical-grade quality.

We also acknowledge the reviewer's point regarding the interpretation of findings. As this study was exploratory in nature, we agree that the results should be interpreted cautiously and that definitive conclusions should be avoided. In line with this approach, we have chosen not to include effect sizes and confidence intervals, as these could imply a level of interpretation beyond the intended scope of the study.

MANUSCRIPT EDITS:

Lines 954 – 956 (Materials and Methods – Biomarker Measurement):

Clinical biomarkers were taken by a Ministry of Health-certified medical clinic via orders placed by a licensed medical professional following assessment for participant health.

Lines 958 – 959 (Materials and Methods – Biomarker Measurement):

Clinical biomarkers were evaluated by a commercial laboratory certified by the Ministry of Health (Singapore).

Lines 1046 – 1048 (Gut Microbiome Dynamics):

Microbiome analyses were conducted by AMILI Pte. Ltd., a precision gut microbiome company with ISO 9001 and ISO 27001 accreditation and established collaborations with government research initiatives in Singapore.

Overall, it is a very innovative study that can be potentially relevant for the field of precision health. However, the results should be interpreted much more cautiously as it is a pilot N=1 research that requires external confirmation.

RESPONSE:

We thank the reviewer for the positive assessment of our manuscript and for pointing out the potential clinical relevance in the context of precision health. Additionally, we also appreciate the reviewer's emphasis on cautious interpretation of results in this pilot N=1 participatory study. In response, we have amended the manuscript to explicitly emphasize that external validation in larger cohorts is required to generalize findings from this study.
