# Supplementary material for: DELTA: Strengthening human biological resilience with an N=1 digital health and dynamic biomarker protocol
Source: PLoS One. 2026 Aug 12;21(8):e0354234. doi: 10.1371/journal.pone.0354234 (PMC13465858; doi:10.1371/journal.pone.0354234)
Supplement: Supplementary file 8 [file pone.0354234.s009.docx]

**DELTA: Strengthening Human Biological Resilience with an N=1 Digital Health and Dynamic Biomarker Protocol**

**Overall Recommendation: Major Revision**

**Summary**

The current manuscript outlines a longitudinal N=1 study that tests a multi-faceted protocol for achieving maximum health status by combining the elements of fasting, physical exercise, dietary changes, wearables and biological marker evaluation. The idea of assessing biological robustness via changes in biomarkers is rather novel and promising, yet the methodology is severely flawed.

**Major Comments**

1. **Overinterpretation of Results**

The paper often refers to biomarker modifications in relation to "metabolic resilience," "adaptation," and "physiological priming." It must be noted that such interpretations are derived from results of a single case study. The research provides valuable information about feasibility and leads for future research, but not about biological processes.

**2. Simultaneous Interventions Prevent Attribution**

The subject was exposed to various interventions at once, which include:

•time-restricted feeding,

•fasting for 48 hours,

•resistance training,

•HIIT training, and

•the Mediterranean diet.

It is, therefore, not possible to ascertain what intervention caused a change in any of the ketone profiles, homocysteine, or ApoB.

**3. Novel Resilience Metrics Require Validation**

The ideas of the proposed Resilience Indicator (RI) and the biomarker system seem intriguing, but there is no information provided about:

•Reproducibility

•Reliability

•Validity

•Predictive capability

It should be clearly stated that these indicators need to be validated independently.

**4. Statistical Inference Is Limited**

There are several conclusions based on visible trends even when there are no statistically significant results. It is necessary that the discussion does not go beyond the results and does not include any assumptions of physiological mechanisms involved.

**5. Potential Participant Bias**

The subject is also the author of the experiment and is highly committed to the intervention. There are high chances of the following bias:

•Mismeasurement

•Misperformance

•Misreporting

These problems must be highlighted in the limitations section.

**Minor Comments**

1. Determine if there were identical conditions when the laboratory tests were carried out in the	experiment.

2. Elaborate on intra-individual biological variability.

3. It may be more helpful to include effect sizes and confidence intervals along with the p- values.

4. Some parts of the discussion have unnecessarily long sections.

**Final Assessment**

This research shows great innovation and could make contributions to the budding science of health monitoring. But conclusions must be greatly qualified due to the exploratory nature of an N=1 experiment.
