# Supplementary material for: DELTA: Strengthening human biological resilience with an N=1 digital health and dynamic biomarker protocol
Source: PLoS One. 2026 Aug 12;21(8):e0354234. doi: 10.1371/journal.pone.0354234 (PMC13465858; doi:10.1371/journal.pone.0354234)
Supplement: Peer review history

# Peer review: Decision Letter 0

**DOI:** 10.1371/journal.pone.0354234.r001

**Authors:** Gbolahan Deji Olatunji

10 Apr 2026

PONE-D-25-65417

DELTA: Fortifying Human Biological Resilience with an N=1 Digital Health and Dynamic Biomarker Protocol

PLOS One

Dear Dr. Ho,

Thank you for submitting your manuscript to PLOS ONE. After careful consideration, we feel that it has merit but does not fully meet PLOS ONE’s publication criteria as it currently stands. Therefore, we invite you to submit a revised version of the manuscript that addresses the points raised during the review process.

Please submit your revised manuscript by May 25 2026 11:59PM. If you will need more time than this to complete your revisions, please reply to this message or contact the journal office at plosone@plos.org. When you're ready to submit your revision, log on to https://www.editorialmanager.com/pone/ and select the 'Submissions Needing Revision' folder to locate your manuscript file.

Please include the following items when submitting your revised manuscript:

- A letter that responds to each point raised by the academic editor and reviewer(s). You should upload this letter as a separate file labeled 'Response to Reviewers'.
- A marked-up copy of your manuscript that highlights changes made to the original version. You should upload this as a separate file labeled 'Revised Manuscript with Track Changes'.
- An unmarked version of your revised paper without tracked changes. You should upload this as a separate file labeled 'Manuscript'.

If you would like to make changes to your financial disclosure, please include your updated statement in your cover letter. Guidelines for resubmitting your figure files are available below the reviewer comments at the end of this letter.

If applicable, we recommend that you deposit your laboratory protocols in protocols.io to enhance the reproducibility of your results. Protocols.io assigns your protocol its own identifier (DOI) so that it can be cited independently in the future. For instructions see: https://journals.plos.org/plosone/s/submission-guidelines#loc-laboratory-protocols. Additionally, PLOS ONE offers an option for publishing peer-reviewed Lab Protocol articles, which describe protocols hosted on protocols.io. Read more information on sharing protocols at https://plos.org/protocols?utm_medium=editorial-email&utm_source=authorletters&utm_campaign=protocols.

As the corresponding author, your ORCID iD is verified in the submission system and will appear in the published article. PLOS supports the use of ORCID, and we encourage all coauthors to register for an ORCID iD and use it as well. Please encourage your coauthors to verify their ORCID iD within the submission system before final acceptance, as unverified ORCID iDs will not appear in the published article. *Only*  the individual author can complete the verification step; PLOS staff *cannot*  verify ORCID iDs on behalf of authors.

We look forward to receiving your revised manuscript.

Kind regards,

Gbolahan Deji Olatunji, M.D, MPH

Academic Editor

PLOS One

Journal Requirements:

When submitting your revision, we need you to address these additional requirements.

1. Please ensure that your manuscript meets PLOS ONE's style requirements, including those for file naming. The PLOS ONE style templates can be found at

https://journals.plos.org/plosone/s/file?id=wjVg/PLOSOne_formatting_sample_main_body.pdf and

https://journals.plos.org/plosone/s/file?id=ba62/PLOSOne_formatting_sample_title_authors_affiliations.pdf.

2. Thank you for stating in your Funding Statement:

" The DELTA study team gratefully acknowledges support from the Institute for Digital Medicine (WisDM) Translational Research Programme (grant no. A-0001319-00-00) at NUS Yong Loo Lin School of Medicine, NUS-NUHS HPHSR Enabling Grant Call (grant no. A-8002819-00-00), and NUS Start-Up Grant (funding no. A-0009368-05-00)."

Please provide an amended statement that declares *all* the funding or sources of support (whether external or internal to your organization) received during this study, as detailed online in our guide for authors at http://journals.plos.org/plosone/s/submit-now.  Please also include the statement “There was no additional external funding received for this study.” in your updated Funding Statement.

Please include your amended Funding Statement within your cover letter. We will change the online submission form on your behalf.

3. Thank you for stating the following financial disclosure:

" The DELTA study team gratefully acknowledges support from the Institute for Digital Medicine (WisDM) Translational Research Programme (grant no. A-0001319-00-00) at NUS Yong Loo Lin School of Medicine, NUS-NUHS HPHSR Enabling Grant Call (grant no. A-8002819-00-00), and NUS Start-Up Grant (funding no. A-0009368-05-00)."

Please state what role the funders took in the study.  If the funders had no role, please state: "The funders had no role in study design, data collection and analysis, decision to publish, or preparation of the manuscript."

If this statement is not correct you must amend it as needed.

Please include this amended Role of Funder statement in your cover letter; we will change the online submission form on your behalf.

4. Thank you for stating the following in the Competing Interests section:

"P.W., N.F., C.S., N.Y.T.L., S.W.S., G.S., Y.S., L.H., Y.H.O., X.T., and D.H. are co-inventors of a digital resilience biomarker and digital twin-based health optimization platform, and are also co-inventors of a pending patent pertaining to GPT-based Healthspan Copilot. P.W., N.Y.T.L, Y.S., Y.H.O., H.S.J.C., X.T., and D.H. are co-inventors of a platform for data-driven adherence gamification. D.H. is a scientific co-founder of KYAN Technologies, which is developing AI-based oncology clinical decision support. D.H. is co-founder and chair of the scientific advisory board of Eternami Global Pte. Ltd., which is a longevity medicine technology platform. D.H. is advisor to Prelude Health Pte. Ltd. and Medicia. J.W.J.L. is a co-Founder of Amili Pte. Ltd. Amili Pte. Ltd. did not play a role in the funding of this work."

We note that one or more of the authors are employed by a commercial company

a.       Please provide an amended Funding Statement declaring this commercial affiliation, as well as a statement regarding the Role of Funders in your study. If the funding organization did not play a role in the study design, data collection and analysis, decision to publish, or preparation of the manuscript and only provided financial support in the form of authors' salaries and/or research materials, please review your statements relating to the author contributions, and ensure you have specifically and accurately indicated the role(s) that these authors had in your study. You can update author roles in the Author Contributions section of the online submission form.

Please also include the following statement within your amended Funding Statement.

“The funder provided support in the form of salaries for authors [insert relevant initials], but did not have any additional role in the study design, data collection and analysis, decision to publish, or preparation of the manuscript. The specific roles of these authors are articulated in the ‘author contributions’ section.”

If your commercial affiliation did play a role in your study, please state and explain this role within your updated Funding Statement.

b. Please also provide an updated Competing Interests Statement declaring this commercial affiliation along with any other relevant declarations relating to employment, consultancy, patents, products in development, or marketed products, etc.

Within your Competing Interests Statement, please confirm that this commercial affiliation does not alter your adherence to all PLOS ONE policies on sharing data and materials by including the following statement: "This does not alter our adherence to  PLOS ONE policies on sharing data and materials.” (as detailed online in our guide for authors http://journals.plos.org/plosone/s/competing-interests) . If this adherence statement is not accurate and  there are restrictions on sharing of data and/or materials, please state these. Please note that we cannot proceed with consideration of your article until this information has been declared.

Please include both an updated Funding Statement and Competing Interests Statement in your cover letter. We will change the online submission form on your behalf.

5. We note that you have a patent relating to material pertinent to this article. Please provide an amended statement of Competing Interests to declare this patent (with details including name and number), along with any other relevant declarations relating to employment, consultancy, patents, products in development or modified products etc. Please confirm that this does not alter your adherence to all PLOS ONE policies on sharing data and materials, as detailed online in our guide for authors http://journals.plos.org/plosone/s/competing-interests by including the following statement: "This does not alter our adherence to  PLOS ONE policies on sharing data and materials.” If there are restrictions on sharing of data and/or materials, please state these. Please note that we cannot proceed with consideration of your article until this information has been declared.

This information should be included in your cover letter; we will change the online submission form on your behalf.

6. Please note that funding information should not appear in the Acknowledgments section or other areas of your manuscript. We will only publish funding information present in the Funding Statement section of the online submission form. Please remove any funding-related text from the manuscript and let us know how you would like to update your Funding Statement.

7. Please include captions for your Supporting Information files at the end of your manuscript, and update any in-text citations to match accordingly. Please see our Supporting Information guidelines for more information: http://journals.plos.org/plosone/s/supporting-information.

8. Please update your submission to use the PLOS LaTeX template. The template and more information on our requirements for LaTeX submissions can be found at http://journals.plos.org/plosone/s/latex.

9. We note that there is identifying data in the Supporting Information file <Supplementary Data 1>. Due to the inclusion of these potentially identifying data, we have removed this file from your file inventory. Prior to sharing human research participant data, authors should consult with an ethics committee to ensure data are shared in accordance with participant consent and all applicable local laws.

Data sharing should never compromise participant privacy. It is therefore not appropriate to publicly share personally identifiable data on human research participants. The following are examples of data that should not be shared:

-Name, initials, physical address

-Ages more specific than whole numbers

-Internet protocol (IP) address

-Specific dates (birth dates, death dates, examination dates, etc.)

-Contact information such as phone number or email address

-Location data

-ID numbers that seem specific (long numbers, include initials, titled “Hospital ID”) rather than random (small numbers in numerical order)

Data that are not directly identifying may also be inappropriate to share, as in combination they can become identifying. For example, data collected from a small group of participants, vulnerable populations, or private groups should not be shared if they involve indirect identifiers (such as sex, ethnicity, location, etc.) that may risk the identification of study participants.

Additional guidance on preparing raw data for publication can be found in our Data Policy (https://journals.plos.org/plosone/s/data-availability#loc-human-research-participant-data-and-other-sensitive-data) and in the following article: http://www.bmj.com/content/340/bmj.c181.long.

Please remove or anonymize all personal information (<specific identifying information in file to be removed>), ensure that the data shared are in accordance with participant consent, and re-upload a fully anonymized data set. Please note that spreadsheet columns with personal information must be removed and not hidden as all hidden columns will appear in the published file.

10. If the reviewer comments include a recommendation to cite specific previously published works, please review and evaluate these publications to determine whether they are relevant and should be cited. There is no requirement to cite these works unless the editor has indicated otherwise.

Additional Editor Comments (if provided):

This is a interesting but quite unusal study where the investigator, the subject, and the patent holder are the same person, the interpretive choices made throughout the manuscript, about which findings to emphasize, how to frame trajectories, what language to use around causation, cannot be fully disentangled from personal investment. While the authors acknowledge this, one would wonder for this means for the conclusions generated

Closely related to this is the GPT Copilot evaluation, which as I noted is essentially the authors confirming that a tool they built and hold a patent on works as they designed it to. That is not validation in any meaningful scientific sense and the paper should say so more clearly. There is no external validation cohort, no benchmark comparison, and no pre-registered performance criteria.

The biological age result is the kind of finding that will attract popular attention and it rests on a relatively thin methodological foundation. A regression model trained on a general population sample and applied to an individual who sits at the extreme healthy end of nearly every input variable is going to produce a dramatic estimate almost by construction. The authors should put this into consideration when making inferences.

The sleep findings are genuinely interesting but the control condition is weak. The retrospective data comes from before the study began, under different monitoring conditions, and without any of the other protocol elements in place. You cannot isolate the sleep timing intervention from everything else that changed simultaneously.

[Note: HTML markup is below. Please do not edit.]

Reviewers' comments:

Reviewer's Responses to Questions

**Comments to the Author**

1. Is the manuscript technically sound, and do the data support the conclusions?

The manuscript must describe a technically sound piece of scientific research with data that supports the conclusions. Experiments must have been conducted rigorously, with appropriate controls, replication, and sample sizes. The conclusions must be drawn appropriately based on the data presented.

Reviewer #1: Yes

Reviewer #2: Yes

**********

2. Has the statistical analysis been performed appropriately and rigorously?

Reviewer #1: Yes

Reviewer #2: Yes

**********

3. Have the authors made all data underlying the findings in their manuscript fully available?

The PLOS Data policy requires authors to make all data underlying the findings described in their manuscript fully available without restriction, with rare exception (please refer to the Data Availability Statement in the manuscript PDF file). The data should be provided as part of the manuscript or its supporting information, or deposited to a public repository. For example, in addition to summary statistics, the data points behind means, medians and variance measures should be available. If there are restrictions on publicly sharing data—e.g. participant privacy or use of data from a third party—those must be specified.

Reviewer #1: Yes

Reviewer #2: Yes

**********

4. Is the manuscript presented in an intelligible fashion and written in standard English?

PLOS ONE does not copyedit accepted manuscripts, so the language in submitted articles must be clear, correct, and unambiguous. Any typographical or grammatical errors should be corrected at revision, so please note any specific errors here.

Reviewer #1: Yes

Reviewer #2: Yes

**********

5. Review Comments to the Author

Please use the space provided to explain your answers to the questions above. You may also include additional comments for the author, including concerns about dual publication, research ethics, or publication ethics. (Please upload your review as an attachment if it exceeds 20,000 characters)

Reviewer #1: Summary

This manuscript outlines a prospective, open-label N=1 case study, designated as the DELTA programme, which investigates a multimodal health optimisation protocol. The study focuses on a single subject, who undertook a regimen comprising Time-Restricted Eating (TRE), a Mediterranean diet, structured exercise, and sleep phase advancement. The authors introduce the concept of ‘dynamic biomarkers’, advocating for the measurement of recovery trajectories (resilience) following controlled stressors, such as a 48-hour fast, in lieu of relying solely on static physiological snapshots. The reported outcomes include improvements in sleep architecture and metabolic flexibility (specifically the glycolysis-ketosis switch), alongside the proposal of a ‘Resilience Index’ for biomarkers such as homocysteine. Additionally, a GPT-based ‘Healthspan Copilot’ is presented as a tool to support adherence.

General Assessment

The manuscript addresses a timely and pertinent subject within the burgeoning fields of digital health and geroscience: the paradigm shift from static ‘sick care’ metrics to the longitudinal, dynamic monitoring of ‘healthspan’. The conceptual framework of assessing biological resilience via controlled stressors is scientifically sound and methodologically intriguing.

However, the manuscript is constrained by significant limitations inherent to its design. Whilst the authors are transparent regarding the N=1 nature of the study and the fact that the subject is the Principal Investigator, the implications of this substantial bias have not been fully mitigated within the analysis. Furthermore, the manuscript currently reads as a concatenation of disparate pilot studies, spanning sleep, microbiome, biomarkers, and AI, rather than a cohesive validation of a unified platform. The section detailed the ‘GPT Healthspan Copilot’, in particular, lacks rigorous validation and appears disjointed from the wet-lab and wearable data. Consequently, I recommend Major Revisions to address these structural and methodological concerns before the manuscript can be considered for publication.

Specific Comments

1. Structure and Organisation

The manuscript attempts to cover a vast array of domains, including metabolism, sleep physiology, microbiome analysis, and artificial intelligence. Whilst ambitious, this breadth has resulted in a fragmented narrative. The transition from biological wet-lab data (e.g., homocysteine and ApoB dynamics) to the digital component is notably abrupt; the GPT section appears as an addendum rather than an integral component of the scientific enquiry. Furthermore, the inclusion of a qualitative interview section with the subject, who is also the author, is methodologically awkward. In a paper focused on objective biological resilience, analysing one’s own ‘participant experience’ introduces a circularity that detracts from the empirical physiological data.

2. Literature Review

The review of the existing literature is generally adequate regarding healthspan and intermittent fasting but lacks necessary depth in two key areas. Firstly, the authors frame ‘dynamic biomarkers’ as a novel introduction; however, oral glucose tolerance tests (OGTT) and cardiac stress tests are established clinical paradigms of dynamic biomarkers. The literature review should better contextualise how the DELTA protocol’s utilisation of fasting as a stressor compares to these existing challenge tests. Secondly, the paper would benefit significantly from a more robust comparison with seminal longitudinal multi-omics studies, such as the iPOP study by Michael Snyder. Differentiating DELTA from these existing deep-phenotyping cohorts is crucial to establishing its unique contribution to the field.

3. Methodology

The methodology presents the most contentious aspect of this work due to the ‘participatory’ design where the subject and the author are the same individual. Whilst described as participatory, it is, in essence, an unblinded self-experiment. The observed behavioural improvements, such as sleep consistency, are heavily confounded by the subject’s intrinsic motivation to generate favourable data for publication. This creates a ‘super-Hawthorne effect’, making it impossible to disentangle the efficacy of the protocol from the motivation of the researcher.

Furthermore, the microbiome analysis contains a fundamental flaw. The study compares fasting states but admits to lacking a true pre-intervention baseline, as the subject had adopted the lifestyle years prior. The comparison with a single ‘3-Meals-A-Day’ (3MAD) week is insufficient; one week is likely too brief to induce stable shifts in gut flora diversity, rendering the findings of ‘non-significant’ bacterial changes largely uninformative. Similarly, the ‘retrospective’ control period for sleep is methodologically weak. Sleep is highly seasonal and context-dependent; comparing a retrospective period to a prospective intervention period without controlling for seasonality or workload variations weakens the causal claim that the DELTA protocol was the sole driver of improved sleep.

4. Model and Analysis

The authors propose a ‘Resilience Index’ for homocysteine calculated as 15 - AUC. This formula appears arbitrary. It is unclear why the baseline constant is set to 15, and whether this is derived from established clinical thresholds or theoretical principles. Without validation on a larger dataset or a derivation from first principles, this metric appears purely heuristic and lacks generalisability.

Regarding statistical analysis, the manuscript frequently employs t-tests and ANOVA on daily measurements (e.g., sleep stages, HRV). In time-series data, daily measurements are often autocorrelated, today’s value is dependent on yesterday’s. Standard ANOVA assumes independence of observations; thus, the effective sample size is likely lower than the number of days implies, and the reported p-values may be overstated. Finally, the validation of the GPT Copilot is anecdotal. There is no quantitative metric for the AI’s performance, such as accuracy against a ground truth or hallucination rates. As presented, it serves as a feature description rather than a rigorous scientific result.

5. Interpretation of Results

The emphasis on the absence of Fusobacteria as a positive outcome is speculative. Whilst this genus is associated with pathology, its absence in an N=1 study could simply be an individual idiosyncrasy rather than a result of the protocol. Additionally, whilst the quadratic fit for ketone recovery is an interesting observation, the claim of a ‘dose-dependent’ correlation is based on very few data points. Deducing a robust correlation or physiological ‘law’ from such sparse data is premature.

Recommendations

To meet the rigour required for publication, the authors are advised to address the following points:

• The language must be strictly curbed. Phrases suggesting this protocol ‘fortifies healthspan’ should be qualified to reflect that it fortified biomarkers in this specific subject, avoiding broader claims of efficacy.

• The authors must address the autocorrelation in the longitudinal data. Instead of simple t-tests between periods, time-series analysis (e.g., ARIMA or change-point analysis) should be considered to demonstrate that shifts in biomarkers were statistically significant deviations from the baseline trend, rather than random drift.

• A stronger justification for the mathematical formulation of the Resilience Index is required. If it cannot be theoretically justified, it should be presented merely as an ‘Area Under the Curve (AUC)’ comparison rather than a novel index.

• The discussion must explicitly address the limitations of the short 3MAD ‘washout’ period, acknowledging that seven days may be insufficient to reshape the microbiome.

• It is strongly recommended that the GPT section be moved to the Supplementary Materials or significantly shortened. It currently lacks the rigour of the physiological sections and distracts from the core biological findings.

• The ‘Limitations’ section must explicitly state that because the subject was the Principal Investigator, behavioural compliance cannot be generalised to a population lacking this specific motivation.

Reviewer #2: This is a highly innovative and fascinating study. Using an N=1 digital health protocol to measure biological resilience and healthspan is a very forward-thinking approach to precision medicine.

I appreciate the methodological rigor and transparency you applied, especially given that the subject is one of the authors. The integration of AI, wearables, and lifestyle interventions (like time-restricted eating and strength training) provides a fantastic proof-of-concept for how we can track dynamic biomarkers in real-time. This will serve as an excellent foundation for larger population-level trials.

I have no changes to request. Congratulations on a unique and well-executed paper.

**********

6. PLOS authors have the option to publish the peer review history of their article (what does this mean?). If published, this will include your full peer review and any attached files.

If you choose “no”, your identity will remain anonymous but your review may still be made public.

**Do you want your identity to be public for this peer review?**  For information about this choice, including consent withdrawal, please see our Privacy Policy.

Reviewer #1: No

Reviewer #2: **Yes:**  Ganesh Praneeth Roy Avula

**********

[NOTE: If reviewer comments were submitted as an attachment file, they will be attached to this email and accessible via the submission site. Please log into your account, locate the manuscript record, and check for the action link "View Attachments". If this link does not appear, there are no attachment files.]

To ensure your figures meet our technical requirements, please review our figure guidelines: https://journals.plos.org/plosone/s/figures

You may also use PLOS’s free figure tool, NAAS, to help you prepare publication quality figures: https://journals.plos.org/plosone/s/figures#loc-tools-for-figure-preparation.

NAAS will assess whether your figures meet our technical requirements by comparing each figure against our figure specifications.

Attachment Submitted filename: Review Report-PONE-D-25-65417.pdf

# Author comment: Author response to Decision Letter 1

**DOI:** 10.1371/journal.pone.0354234.r002

7 May 2026

We have revised the manuscript to meet the journal requirement as outlined by the editorial team. In response to the reviewer comments, we have provided our detailed responses and revisions in the "DELTA Reviewer Responses" document.

Attachment Submitted filename: DELTA Reviewer Responses.pdf

# Peer review: Decision Letter 1

**DOI:** 10.1371/journal.pone.0354234.r003

**Authors:** Gbolahan Deji Olatunji

22 Jun 2026

PONE-D-25-65417R1

DELTA: Strengthening Human Biological Resilience with an N=1 Digital Health and Dynamic Biomarker Protocol

PLOS One

Dear Dr. Ho,

Thank you for submitting your manuscript to PLOS ONE. After careful consideration, we feel that it has merit but does not fully meet PLOS ONE’s publication criteria as it currently stands. Therefore, we invite you to submit a revised version of the manuscript that addresses the points raised during the review process.

==============================

**ACADEMIC EDITOR:**

While the majority of the reviewer and editorial concerns have been responded to, I would like to note a few remaining points from the latest round of review for your attention and action below

* This is a interesting but quite unusal study where the investigator, the subject, and the patent holder are the same person, the interpretive choices made throughout the manuscript, about which findings to emphasize, how to frame trajectories, what language to use around causation, cannot be fully disentangled from personal investment. While the authors acknowledge this, one would wonder for this means for the conclusions generated

* Closely related to this is the GPT Copilot evaluation, which as I noted is essentially the authors confirming that a tool they built and hold a patent on works as they designed it to. That is not validation in any meaningful scientific sense and the paper should say so more clearly. There is no external validation cohort, no benchmark comparison, and no pre-registered performance criteria.

* The biological age result is the kind of finding that will attract popular attention and it rests on a relatively thin methodological foundation. A regression model trained on a general population sample and applied to an individual who sits at the extreme healthy end of nearly every input variable is going to produce a dramatic estimate almost by construction. The authors should put this into consideration when making inferences.

* The sleep findings are genuinely interesting but the control condition is weak. The retrospective data comes from before the study began, under different monitoring conditions, and without any of the other protocol elements in place. You cannot isolate the sleep timing intervention from everything else that changed simultaneously.

Please submit your revised manuscript by Aug 06 2026 11:59PM. If you will need more time than this to complete your revisions, please reply to this message or contact the journal office at plosone@plos.org. When you're ready to submit your revision, log on to https://www.editorialmanager.com/pone/ and select the 'Submissions Needing Revision' folder to locate your manuscript file.

Please include the following items when submitting your revised manuscript:

- A letter that responds to each point raised by the academic editor and reviewer(s). You should upload this letter as a separate file labeled 'Response to Reviewers'.
- A marked-up copy of your manuscript that highlights changes made to the original version. You should upload this as a separate file labeled 'Revised Manuscript with Track Changes'.
- An unmarked version of your revised paper without tracked changes. You should upload this as a separate file labeled 'Manuscript'.

If you would like to make changes to your financial disclosure, please include your updated statement in your cover letter. Guidelines for resubmitting your figure files are available below the reviewer comments at the end of this letter.

If applicable, we recommend that you deposit your laboratory protocols in protocols.io to enhance the reproducibility of your results. Protocols.io assigns your protocol its own identifier (DOI) so that it can be cited independently in the future. For instructions see: https://journals.plos.org/plosone/s/submission-guidelines#loc-laboratory-protocols. Additionally, PLOS ONE offers an option for publishing peer-reviewed Lab Protocol articles, which describe protocols hosted on protocols.io. Read more information on sharing protocols at https://plos.org/protocols?utm_medium=editorial-email&utm_source=authorletters&utm_campaign=protocols.

As the corresponding author, your ORCID iD is verified in the submission system and will appear in the published article. PLOS supports the use of ORCID, and we encourage all coauthors to register for an ORCID iD and use it as well. Please encourage your coauthors to verify their ORCID iD within the submission system before final acceptance, as unverified ORCID iDs will not appear in the published article. *Only*  the individual author can complete the verification step; PLOS staff *cannot*  verify ORCID iDs on behalf of authors.

We look forward to receiving your revised manuscript.

Kind regards,

Gbolahan Deji Olatunji, M.D, MPH

Academic Editor

PLOS One

Journal Requirements:

If the reviewer comments include a recommendation to cite specific previously published works, please review and evaluate these publications to determine whether they are relevant and should be cited. There is no requirement to cite these works unless the editor has indicated otherwise.

Please review your reference list to ensure that it is complete and correct. If you have cited papers that have been retracted, please include the rationale for doing so in the manuscript text, or remove these references and replace them with relevant current references. Any changes to the reference list should be mentioned in the rebuttal letter that accompanies your revised manuscript. If you need to cite a retracted article, indicate the article’s retracted status in the References list and also include a citation and full reference for the retraction notice.

[Note: HTML markup is below. Please do not edit.]

Reviewers' comments:

Reviewer's Responses to Questions

**Comments to the Author**

1. If the authors have adequately addressed your comments raised in a previous round of review and you feel that this manuscript is now acceptable for publication, you may indicate that here to bypass the “Comments to the Author” section, enter your conflict of interest statement in the “Confidential to Editor” section, and submit your "Accept" recommendation.

Reviewer #1: (No Response)

Reviewer #3: (No Response)

**********

2. Is the manuscript technically sound, and do the data support the conclusions?

The manuscript must describe a technically sound piece of scientific research with data that supports the conclusions. Experiments must have been conducted rigorously, with appropriate controls, replication, and sample sizes. The conclusions must be drawn appropriately based on the data presented.

Reviewer #1: Yes

Reviewer #3: Yes

**********

3. Has the statistical analysis been performed appropriately and rigorously?

Reviewer #1: Yes

Reviewer #3: Yes

**********

4. Have the authors made all data underlying the findings in their manuscript fully available?

The PLOS Data policy requires authors to make all data underlying the findings described in their manuscript fully available without restriction, with rare exception (please refer to the Data Availability Statement in the manuscript PDF file). The data should be provided as part of the manuscript or its supporting information, or deposited to a public repository. For example, in addition to summary statistics, the data points behind means, medians and variance measures should be available. If there are restrictions on publicly sharing data—e.g. participant privacy or use of data from a third party—those must be specified.

Reviewer #1: Yes

Reviewer #3: Yes

**********

5. Is the manuscript presented in an intelligible fashion and written in standard English?

PLOS ONE does not copyedit accepted manuscripts, so the language in submitted articles must be clear, correct, and unambiguous. Any typographical or grammatical errors should be corrected at revision, so please note any specific errors here.

Reviewer #1: Yes

Reviewer #3: Yes

**********

6. Review Comments to the Author

Please use the space provided to explain your answers to the questions above. You may also include additional comments for the author, including concerns about dual publication, research ethics, or publication ethics. (Please upload your review as an attachment if it exceeds 20,000 characters)

Reviewer #1: The authors have responded to my comments and concerns. I recommend accepting the paper for publication.

Reviewer #3: This paper highlights an exciting case report study that evaluates a health optimization protocol through fasting, physical activity, dietary changes, wearables, and biomarker measurements. The idea of exploring biological resilience via changes in dynamic biomarkers is a fresh one and might prove very useful in precision health. Nevertheless, several important issues need to be addressed before the publication of this research.

First of all, there is a problem with the overinterpretation of results. The manuscript often makes conclusions on metabolic resilience, adaption, and priming in relation to changes in biomarkers. This is not entirely correct because this is a N=1 longitudinal design with no control groups used. Therefore, it would be better to consider this information just as exploratory.

Another significant issue that needs to be discussed is the combination of several interventions: time-restricted eating, fasting, resistance and high-intensity interval training, diet adjustment, and supplementation. The fact is that this study did not evaluate any particular intervention separately. Hence, it becomes impossible to find out what interventions have led to metabolic, athletic performance, or biomarker changes.

The suggested Resilience Indicator as well as biomarkers are very promising concepts. However, there is no data on reproducibility, reliability, prediction, and clinical validity of the measures, and they have to be treated as initial ones that require confirmation in larger and more diversified samples.

Furthermore, it would also be useful to improve statistical analysis since several conclusions seem to be driven mostly by visual data analysis even in the absence of significant effects. It is necessary to distinguish more clearly between results of data analysis and hypotheses.

In addition, another possible threat to validity is that the participant who took part in the research is an author. While it is noted in limitations, it should be discussed more thoroughly.

Some minor points include specifying laboratory procedures of sample standardization, providing information about intra-individual variation in the study participants, and including effect sizes and confidence intervals in analysis.

Overall, it is a very innovative study that can be potentially relevant for the field of precision health. However, the results should be interpreted much more cautiously as it is a pilot N=1 research that requires external confirmation.

**********

7. PLOS authors have the option to publish the peer review history of their article (what does this mean?). If published, this will include your full peer review and any attached files.

If you choose “no”, your identity will remain anonymous but your review may still be made public.

**Do you want your identity to be public for this peer review?**  For information about this choice, including consent withdrawal, please see our Privacy Policy.

Reviewer #1: No

Reviewer #3: No

**********

[NOTE: If reviewer comments were submitted as an attachment file, they will be attached to this email and accessible via the submission site. Please log into your account, locate the manuscript record, and check for the action link "View Attachments". If this link does not appear, there are no attachment files.]

To ensure your figures meet our technical requirements, please review our figure guidelines: https://journals.plos.org/plosone/s/figures

You may also use PLOS’s free figure tool, NAAS, to help you prepare publication quality figures: https://journals.plos.org/plosone/s/figures#loc-tools-for-figure-preparation.

NAAS will assess whether your figures meet our technical requirements by comparing each figure against our figure specifications.

Attachment Submitted filename: manuscript review 1 for plos.docx

# Author comment: Author response to Decision Letter 2

**DOI:** 10.1371/journal.pone.0354234.r004

30 Jun 2026

We thank the Editor and Reviewers for their continued evaluation of our manuscript and for the additional perspectives provided during the review process. Please see our responses in the "Response to Reviewers" pdf document.

Attachment Submitted filename: Response to Reviewers.pdf

# Editor report: Decision Letter 2

**DOI:** 10.1371/journal.pone.0354234.r005

**Authors:** Gbolahan Deji Olatunji

6 Jul 2026

DELTA: Strengthening Human Biological Resilience with an N=1 Digital Health and Dynamic Biomarker Protocol

PONE-D-25-65417R2

Dear Dr. Ho,

We’re pleased to inform you that your manuscript has been judged scientifically suitable for publication and will be formally accepted for publication once it meets all outstanding technical requirements.

Within one week, you’ll receive an e-mail detailing the required amendments. When these have been addressed, you’ll receive a formal acceptance letter and your manuscript will be scheduled for publication.

An invoice will be generated when your article is formally accepted. Please note, if your institution has a publishing partnership with PLOS and your article meets the relevant criteria, all or part of your publication costs will be covered. Please make sure your user information is up-to-date by logging into Editorial Manager at Editorial Manager® and clicking the ‘Update My Information' link at the top of the page. For questions related to billing, please contact billing support.

If your institution or institutions have a press office, please notify them about your upcoming paper to help maximize its impact. If they’ll be preparing press materials, please inform our press team as soon as possible -- no later than 48 hours after receiving the formal acceptance. Your manuscript will remain under strict press embargo until 2 pm Eastern Time on the date of publication. For more information, please contact onepress@plos.org.

Kind regards,

Gbolahan Deji Olatunji, M.D, MPH

Academic Editor

PLOS One

Additional Editor Comments (optional):

Reviewers' comments:

# Editor report: Acceptance letter

**DOI:** 10.1371/journal.pone.0354234.r006

**Authors:** Gbolahan Deji Olatunji

PONE-D-25-65417R2

PLOS One

Dear Dr. Ho,

I'm pleased to inform you that your manuscript has been deemed suitable for publication in PLOS One. Congratulations! Your manuscript is now being handed over to our production team.

At this stage, our production department will prepare your paper for publication. This includes ensuring the following:

* All references, tables, and figures are properly cited

* All relevant supporting information is included in the manuscript submission,

* There are no issues that prevent the paper from being properly typeset

You will receive further instructions from the production team, including instructions on how to review your proof when it is ready. Please keep in mind that we are working through a large volume of accepted articles, so please give us a few days to review your paper and let you know the next and final steps.

Lastly, if your institution or institutions have a press office, please let them know about your upcoming paper now to help maximize its impact. If they'll be preparing press materials, please inform our press team within the next 48 hours. Your manuscript will remain under strict press embargo until 2 pm Eastern Time on the date of publication. For more information, please contact onepress@plos.org.

You will receive an invoice from PLOS for your publication fee after your manuscript has reached the completed accept phase. If you receive an email requesting payment before acceptance or for any other service, this may be a phishing scheme. Learn how to identify phishing emails and protect your accounts at https://explore.plos.org/phishing.

If we can help with anything else, please email us at customercare@plos.org.

Thank you for submitting your work to PLOS ONE and supporting open access.

Kind regards,

PLOS ONE Editorial Office Staff

on behalf of

Dr. Gbolahan Deji Olatunji

Academic Editor

PLOS One
